# Supplementary material for: Vegan diet in young children remodels metabolism and challenges the statuses of essential nutrients
Source: EMBO Mol Med. 2021 Jan 20;13(2):e13492. doi: 10.15252/emmm.202013492 (PMC7863396; doi:10.15252/emmm.202013492)
Supplement: Supplementary file 1 — Appendix [file EMMM-13-e13492-s001.pdf]

Vegan diet in young children remodels metabolism and challenges the statuses of essential nutrients

Appendix

Contents

Appendix Supplementary Methods **2**
Supplement 1: Analysis of dietary data 2
Online questionnaires 2
Food record 2
Proportion of retinoids in vitamin A intake 3
Supplement 2: Extraction protocols for untargeted metabolomics 4
Supplement 3: Extended description of statistical methods 5
Biomarkers for vitamin A status 5
Permutation tests for assessing statistical significance 5
Supplement 3c: GSEA-based pathway analysis 7
Supplement 4: Links to R code used in analysis 9

Appendix Figures **10**
Appendix Figure S1: Participant flow chart 10
Appendix Figure S2: Free fatty acids and molecules with one fatty acid in untargeted metabolomics 11
Appendix Figure S3: Phospholipids in untargeted metabolomics 12
Appendix Figure S4: Different triglyceride molecules in untargeted metabolomics 13
Appendix Figure S5: Sphingolipids in untargeted metabolomics 14
Appendix Figure S6: Correlation between different fatty acid carrying molecules and serum cholesterol 15

Appendix Tables **16**
Appendix Table S1: List of measured targeted metabolites 16
Appendix Table S2: Pathway analysis results 17
Appendix Table S3: Serum bile acid level sums 19
Appendix Table S4: Amino acids and fatty acids in untargeted metabolome 20
Appendix Table S5: Correlation of essential amino acids with MUAC and transthyretin 21
Appendix Table S6: List of metabolites recognized by untargeted metabolomics 22
Appendix Table S7: Nutrient intakes in proportion to energy intake 58
Appendix Table S8: Exact p-values of Figure 4 59

References **60**

Appendix Supplementary Methods

Supplement 1: Analysis of dietary data

Online questionnaires

We emailed the parents links to four short online questionnaires inquiring background information of the parents and the participating child as well as the food consumption of the responding parent(s) and the participating child. These included questions on the type of fat and salt usually used in cooking and whether salt is usually added when boiling porridge, rice, pasta and potatoes. A question whether the child has been given dietary supplements during the past month, and if yes, what is the product name, dosage and frequency of use, was also included. Information on the nutrient values of dietary supplements was gathered into an Excel table from producers' internet pages and package labels.

Food record

We sent a four-day food record to parents of each participant and assigned the dates (three weekdays and one day on the weekend) for filling in the record. The instruction page of the food records advised parents to record all foods and beverages that their child consumed during the recording days, except for what they consumed at preschool. An example page was also included. We provided the families with a validated[S1] Children's Food Picture Book[S2], designed to assist in portion size estimation. The parents were instructed to list all the ingredients of composite dishes and to estimate portion sizes using the picture book, weighing, household measures such as teaspoons or tablespoons, or package labels. For packed food products, the exact brand and product name was required.

We gave the day care personnel a separate pre-coded food record for recording food consumption at the day care centre on the dates matching the home food record. We instructed the personnel orally, and the food record included written instructions. Breakfast, lunch, afternoon snack and possible additional snacks each had predefined sections. Different food groups, such as main courses, side dishes (potatoes, pasta, rice), and salad at lunch each had predetermined rows. We also gave the personnel the Children's Food Picture Book and they could also estimate the amounts in household measures.

The food data were recorded using AivoDiet dietary software, which included the Fineli Food Composition Database Release 16 (2013) of the National Institute for Health and Welfare. We had previously updated this as described elsewhere[S3]. We also added new food items, especially vegan products, to the database when necessary. We entered the nutrient values of fortified food products as per information on package labels. The database includes recipes for typical Finnish mixed dishes. For each individual meal, the research assistant used a suitable recipe from the database, modified an existing recipe, or created a new recipe according to the parents' reports. The salt content of home dishes was based on the recipes in the database and we also made use of the information on the type of fat and the type and use of salt in the household gained form the online questionnaires. For food eaten at day care, we used recipes provided by the city of Helsinki's early childhood education and care food services. The food services also provided information on the products (e.g. milk, non-dairy milk substitutes, bread, fat spread, yoghurts) used at each day care centre.

After data entry, we checked for outlying values of food consumption in grams and outlying energy and nutrient intakes. After extracting the data from the software, each food code (food item or mixed dish) appearing in the data set was assigned to a food group and nutrient retention factors[S4] were applied using a single factor per nutrient per food group. The food records were kept between May and October 2017. Two omnivores and three vegetarians had missing or incomplete recordings for one to three days and we omitted these days from the analysis. We included all four days of data in the analysis for the rest of the participants. To calculate total intake from food and supplements, we combined the intake data from dietary supplements with the food record data in R.

Proportion of retinoids in vitamin A intake

Values for retinol and carotenoids were not available in our food composition database and thus the intake of different forms of vitamin A could not be directly calculated. Therefore, we calculated the proportion of retinoids in vitamin A manually by disaggregating the food consumption data into ingredients, multiplying the weight of each food containing retinoid naturally or by fortification with its retinoid content per gram. To find the retinoid values, we used product details available online and food composition databases. The proportion of retinoids in the total RAE intake was calculated as follows:

$$\text{retinoid-}\% = \frac{\sum \text{retinoids in uncooked ingredients } (\mu\text{g}) \text{ for the diet group}}{\sum \text{RAE in uncooked ingredients } (\mu\text{g}) \text{ for the diet group}} * 100\%$$

Supplement 2: Extraction protocols for untargeted metabolomics

The following protocol was used for extraction of metabolome and lipidome for untargeted metabolomics: Frozen serum samples were thawed on ice, 20ml of serum was mixed to a microtube with 180ml 80% methanol for metabolomics or 10ml of serum with 190ml 100% isopropanol for lipidomics. Solutions were vortexed for 15 seconds, incubated at 4°C for 1 hour and centrifuged at 14000g for 15 minutes to achieve supernatant that was transferred to another microtube and frozen to -20°C until analysis.

Supplement 3: Extended description of statistical methods

Biomarkers for vitamin A status

We used two approaches for measuring vitamin A status in the participants.

Retinol-binding protein (RBP) has been used as a biomarker for vitamin A deficiency in large epidemiological studies. We used similar equipment and limits for insufficiency (<1·17mmol/l) and deficiency (<0·83mmol/l) as Engle-Stone et al.[S5] who reported sensitivity and specificity of 94·7% and 88·9% for RBP in predicting vitamin A deficiency in children.

In addition, we used a regression model of RBP, transthyretin and CRP described by Talsma et al.[S6] for vitamin A status assessment. For convenience, we made slight adjustments to the model that do not affect its specificity and sensitivity (Talsma et al. reported AUC of 0·98): We adjusted the constant of the model by -0·496 so that the decision limit of the model was 0 instead of the original 0·496. We also multiplied the estimated regression coefficient by -1 so that the deficiency was denoted by a negative value of the model instead of a positive value.

Permutation tests for assessing statistical significance

In our study, several aspects contradict the use of Student's t-test. We had a relatively small sample size, making it impossible to evaluate predict if the concentration of each measured metabolite is normally distributed in reference population. Furthermore, some measurements include multiple outlier suspects. With small sample size it is problematic to exclude these values from analyses as there is no credible reason to believe the outliers would result from measurement-related rather than physiological reasons. However, these outliers have a strong effect on estimates of variance with small sample size. This decreases the power of e.g. Student's t-test. In addition, there may be age- and sex-dependent variance in physiological concentrations of different metabolites, particularly in children. This effect should be considered while conducting statistical tests.

Therefore, we tailored a following age- and sex-adjusted non-parametric permutation test for our study, which is robust against outliers and does not rely on the unverifiable assumption of normality or equivariance. Recently, the use of such tests and probability index models have become more popular[S7,S8]

Stratification and the measure of difference Let us assume two datasets, X and Y, representing observed concentrations of a metabolite in two groups, say omnivore and vegan groups. Sample sizes may be unequal. For each data point, we have an ID label, age and sex of the corresponding participant and measured metabolite concentration.

Age and sex information is used in *stratification* (dividing the participants to age- and sex-standardized groups). With small sample size we encounter a risk of empty age-sex strata. Thus, it is necessary to divide strata by age somewhat sparsely. In our study, as there was only 6 vegans, we were forced to use only two age classes: (1) under 4-year-olds and (2) at least 4-year-olds. This yields four strata.

To avoid unnecessary outlier removal, we will use a non-parametric *probability index* as a measure of difference between these datasets. Probability index $P(X_i > Y_j)$ is defined as the probability that randomly selected individual values from each dataset, X_i and Y_j, satisfy the inequality $X_i > Y_j$. With a small sample size and decent computing power it is possible to compute these inequalities, a *brute force* method of checking this inequality over all possible datapairs of observations ($n_X \cdot n_X$ operations) in the sample is eligible.

Forming null distribution by permutation First, we estimate a statistical null distribution of the probability index based on our data.[S9] For k rounds:

1. Step 1: Randomly permute the group ID labels *inside* each stratum. This basically divides the participants randomly to two groups in an age- and sex-standardized manner while also maintaining the group sizes.
2. Step 2: Calculate $P(X_i > Y_j)$ with the permuted group labels and calculate quadratic deviation from the null $P(X_i > Y_j) = 0.5$ to be the test statistic. Save test statistics to a vector $T = (T_1, T_2, \ldots, T_k)$ that will eventually form the null distribution.

In our simulations, we used k = 47500[1] that took approximately 330 seconds to run with the datasets of 24 controls and 6 vegans and 6-year-old 1·90GHz processor.

Statistical test To use proper two-sided statistical test, we will use a squared test statistic. As probability index $P(X_i > Y_j) \in [0, 1]$ and comparing two identical datasets yields $P(X_i > Y_j) = 0.5$, we used a test statistic

$$T = (P(X_i > Y_j) - 0.5)^2.$$

After forming the null distribution or probability indexes, we transform it to test statistics and compare the test statistic from the original data, t_{real}, to the null distribution to achieve approximated p-value:

$$p \approx P(t_{\text{real}} > T).$$

Finally, the value of the test statistic from the original data (with the real group labels), t_{real} is used with its the null distribution. After forming the null distribution or probability indexes, we transform it to test statistics and compare the test statistic from the original data, t_{real}, to the null distribution to estimate the approximated p-value:

$$\hat{p} \approx \hat{P}(T > t_{\text{real}} > T) = \frac{\sum_{t \in T}[T > t_{\text{real}}]}{k}.$$

[1]The null distribution is generated randomly every time the test is run. Thus, p-value will be a random variable. Choosing high k will yield more accurate null distribution (smaller variance of p-value) while requiring more computing power or time. We selected k by requiring a standard error of 0·001 at the significance level ($\alpha = 0.05$). Standard error of a p-value in a permutation test is $\sqrt{\frac{p(1-p)}{k}}$ (see e.g. Efron & Tibshirani, *An Introduction to the Bootstrap*, Chapman & Hall, 1993). From this, we have a $k = \frac{0.05*0.95}{0.001^2} = 47500$.

Supplement 3c: GSEA-based pathway analysis

Gene-Set Enrichment Analysis (GSEA) was originally introduced by Subramanian et al.[S10] for obtaining pathway-level knowledge from complex gene expression datasets. We used a GSEA-based approach with HMDB 3.0 pathway database as a framework for metabolome-level pathway analysis. The algorithm was carried out in R and the principles are discussed in the following.

Data preprocessing and statistical significance of metabolite-level change Original data from untargeted metabolomics can be seen in Table S3. For each ion found, there are usually multiple alternative metabolites that correspond to same mass per charge -ratio, thus indistinguishable by mass spectrometer. In total, our data had 10155 alternative metabolites contributing to data signal. We matched each of these metabolite ID's to HMDB 3.0 pathway database to discover the amount of metabolites for each pathway that was recognized at all in our data. Metabolites in one pathway have very similar mass per charge -ratios as many modifications include deleting or adding single ions. This poses an obvious problem of metabolites of a certain pathway appearing under the same mass per charge -peak in mass spectrometry. To avoid overestimation of amount of "found metabolites", we allowed counting each ion for each pathway only once. For example, out of 49 metabolites in bile acid biosynthesis pathway, total of 23 metabolites were found in the list of alternative metabolites. 10 of these were not included in assessing statistical significance, as they appeared under the same mass per charge -peak as others, yielding 13 found metabolites. However, these 10 discarded metabolites may contribute to statistical significance of another pathway, assuming they were the only metabolite with same mass per charge in that particular pathway. List of pathways in the database and corresponding data can be seen in Table S2.

These found metabolites were further divided to significantly changed metabolites. Significance criteria in our pathway analysis was that fold change for the metabolite had to be > 0.25 and uncorrected p-value had to be < 0.05. As the goal of pathway analysis is to recognize changes that correlate in one pathway, it is not necessary to use as tight significance levels as when analyzing single metabolites. Currently, there is no golden standard for pathway analysis significance levels, partly because there is no golden standard for pathway analysis method altogether.

Statistical significance of pathway-level change with Fisher's exact test We analyzed each pathway separately for positive changes, negative changes and all changes regardless of direction. In contrast to GSEA, we did not use permutation tests based on Enrichment Score for assessing pathway-level statistical significance. To save computation time, we preferred Fisher's exact test with null hypothesis that the proportion of significant changes to certain direction in a set of found metabolites in pathway i, is equal to the proportion of significantly changed ions to that direction in the set of all found ions[2]:

$$H_0 : \frac{n_{i,sig.}}{n_i} = \frac{n_{ions,sig.}}{n_{ions}}$$
$$H_1 : \frac{n_{i,sig.}}{n_i} \neq \frac{n_{ions,sig.}}{n_{ions}}.$$

P-values from Fisher's exact test were corrected for multiple hypothesis testing with Benjamini-Hochberg method.

GSEA-based Enrichment Score as a measure of pathway impact Enrichment Score (ES) describes how well changes in metabolite levels of a certain pathway correlate with each other. If a pathway has multiple significant changes to same direction, this pathway will have high ES. If significant changes are randomly scattered in terms of direction and magnitude, the pathway will have low ES. Thus, ES is affected by (1) the magnitude and (2) amount of changes in metabolite levels of a pathway as well as (3) consistency

[2]Comparison is with all found ions because each ion is allowed to be counted only once per pathway

of changes. We calculated ES for each pathway as described by Subramanian et al.[S10] with the exception that we used fold change instead of correlation to sort metabolites according to their magnitude of change. In addition, only the significant changes, i.e. the changes satisfying the conditions defined above, were counted for the Enrichment Score cumulative function.

Disadvantages of using Enrichment Score as a measure of pathway impact include overestimating the impact on pathways with high amount of metabolites.

Supplement 4: Links to R code used in analysis

All R codes used in statistical analysis and building figures for this article are open access in GitHub:

`https://github.com/topihovinen/mirahelsinki`

As the small sample size could risk the anonymity of our participants, we chose not to publish the raw data file online.

Appendix Figures

Appendix Figure S1: Participant flow chart

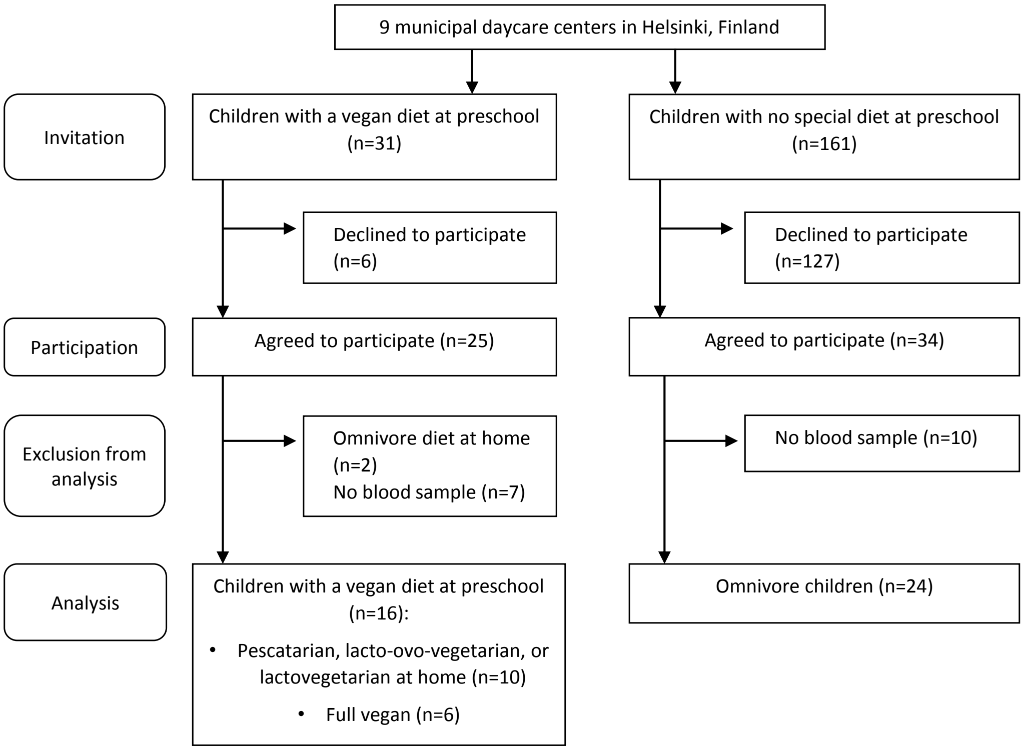

Figure S1: Participant flow chart

Appendix Figure S2: Free fatty acids and molecules with one fatty acid in untargeted metabolomics

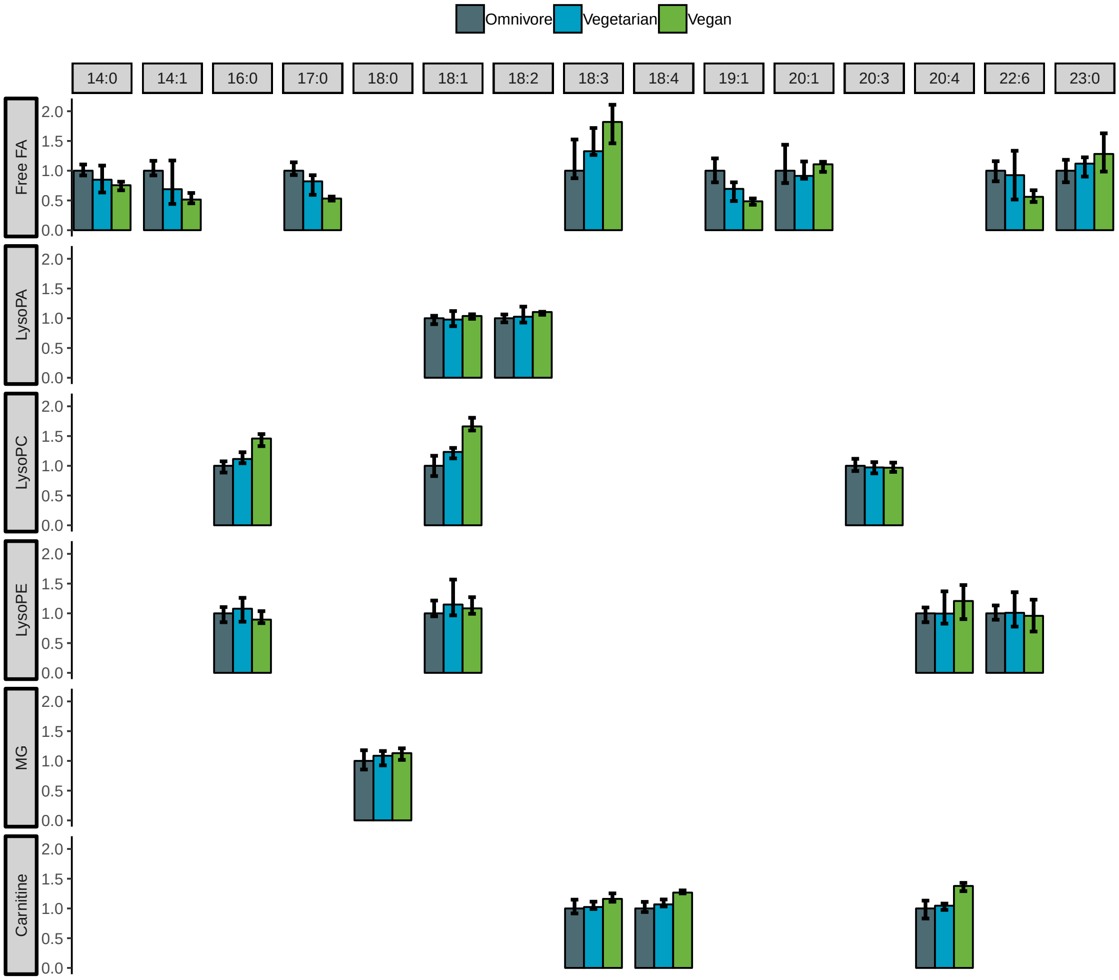

Figure S2: Molecules including a single fatty acid detected by untargeted metabolomics. The figure is grouped by fatty acid length and molecule type. Notation of fatty acid is *"number of carbon atoms : number of double bonds"*. Each bar represents group median and error bar is interquartile range. FA = fatty acid, LysoPA = lysophosphatidic acid, LysoPC = lysophosphatidylcholine, LysoPE = lysophosphatidylethanolamine, MG = monoglyceride.

Appendix Figure S3: Phospholipids in untargeted metabolomics

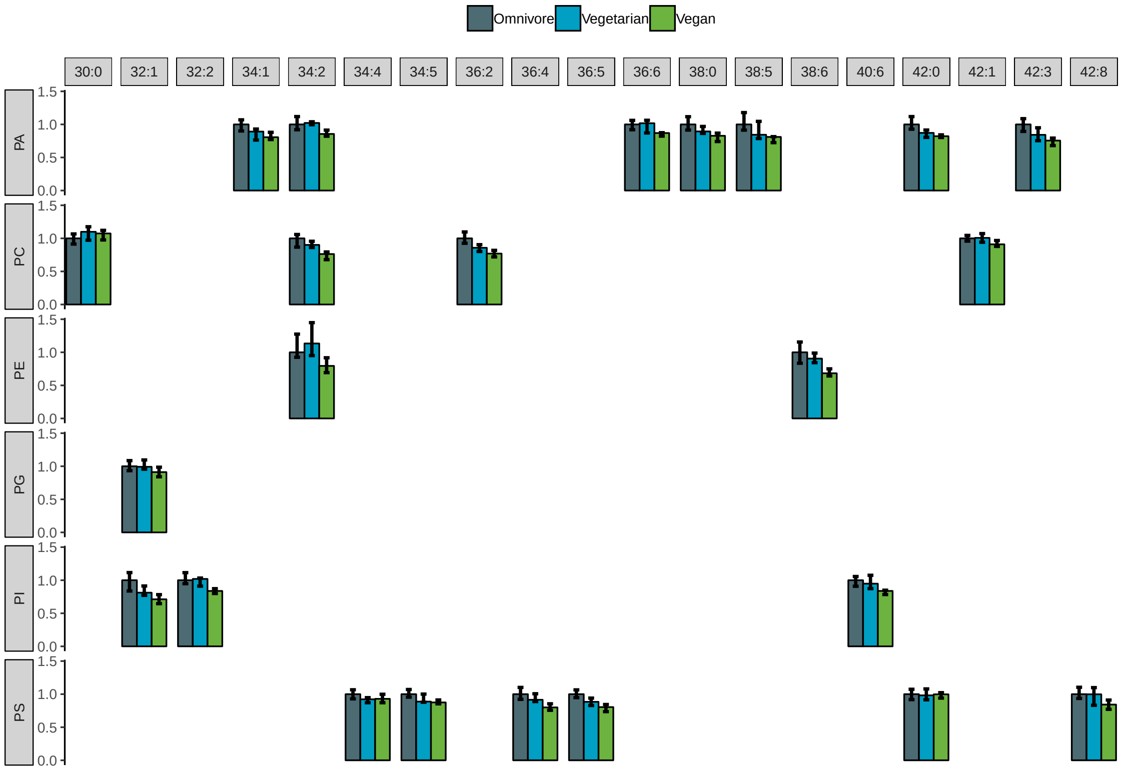

Figure S3: Phospholipids detected by untargeted metabolomics. Phospholipids include two fatty acid tails, and the figure is grouped by sum of fatty acid length/double bonds, and phospholipid type. Notation of fatty acid is *"sum of carbon atoms in the two fatty acids : sum of double bonds in the two fatty acids"*. Each bar represents group median and error bar is interquartile range. Note that almost all detected phospholipids are found in smaller amounts in vegan serum than in omnivores and vegetarians. However, this is most probably related to most serum phospholipids being located in lipoprotein particles, and thus correlating well with low cholesterol in vegans (see figure S6). PA = phosphatidic acid, PC = phosphatidylcholine, PE = phosphatidylethanolamine, PG = phosphatidylglycerol, PI = phosphatidylinositol, PS = phosphatidylserine.

Appendix Figure S4: Different triglyceride molecules in untargeted metabolomics

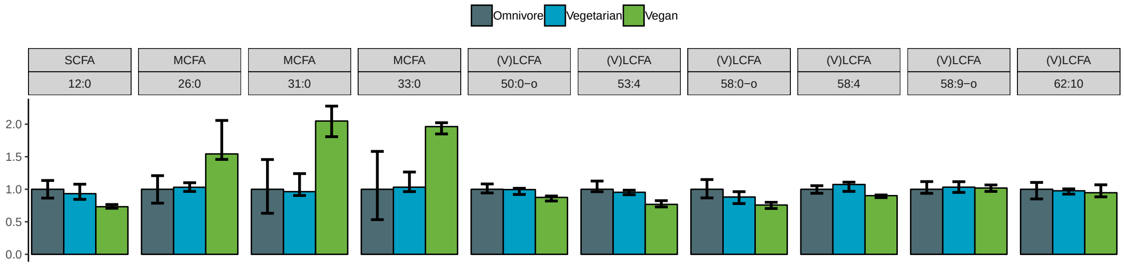

Figure S4: Triglycerides detected by untargeted metabolomics. The figure is grouped by fatty acid length and molecule type. Notation of fatty acid is *"sum of carbon atoms in the two fatty acids : sum of double bonds in the two fatty acids"*. Each bar represents group median and error bar is interquartile range. MCFA = medium-chain fatty acid (the triglyceride includes most probably three fatty acids with carbon atoms between 8-13 in each), SCFA = short-chain fatty acid (the triglyceride includes most probably three fatty acids with carbon atoms between 3-7 in each), (V)LCFA = (very) long-chain fatty acid (the triglyceride includes most probably three fatty acids with at least 14 carbon atoms in each).

Appendix Figure S5: Sphingolipids in untargeted metabolomics

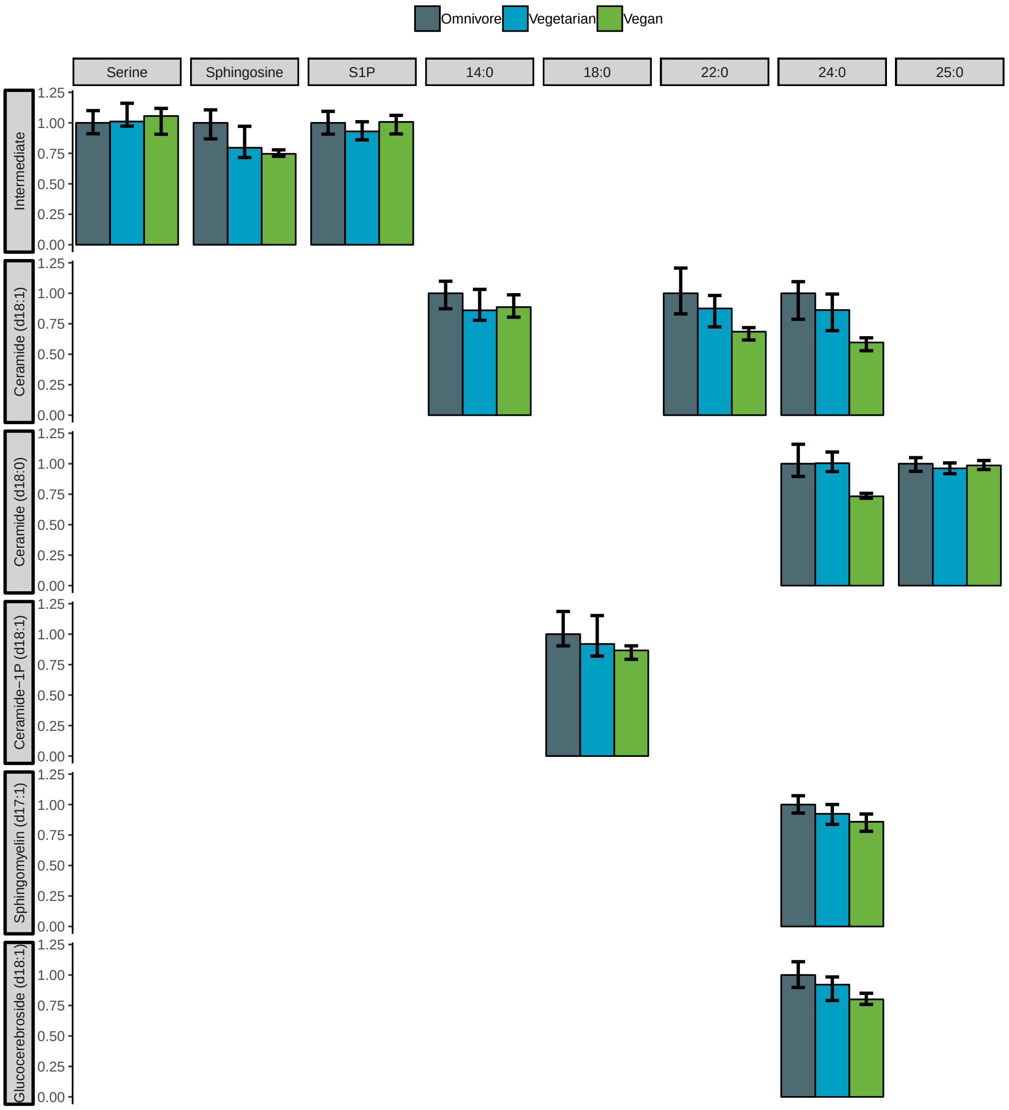

Figure S5: Sphingolipids and their metabolites detected by untargeted metabolomics. The figure is grouped by fatty acid length and molecule type. Notation of fatty acid is *"number of carbon atoms : number of double bonds"*. Each bar represents group median and error bar is interquartile range. S1P = sphingosine-1-phosphate.

Appendix Figure S6: Correlation between different fatty acid carrying molecules and serum cholesterol

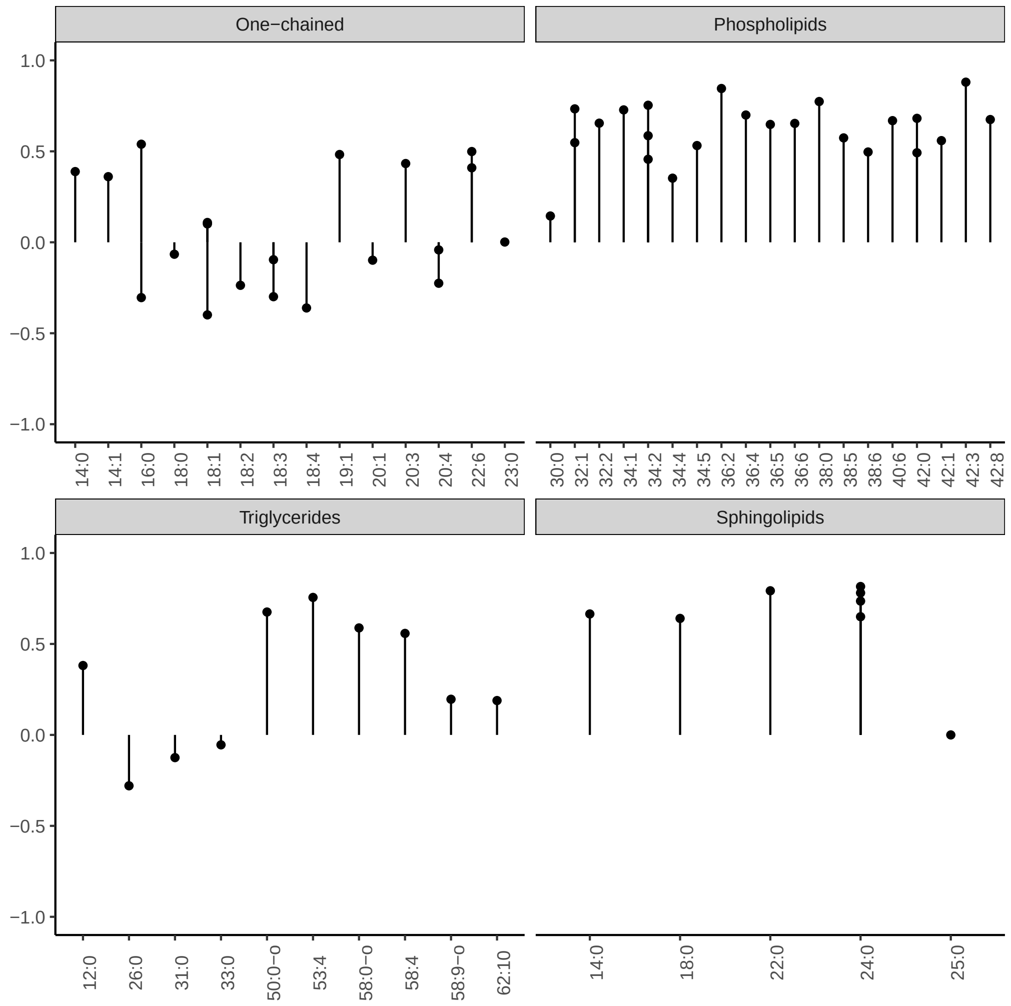

Figure S6: Correlation between cholesterol and detected molecules with one, two or three fatty acids. Nearly all phospholipids, sphingolipids and (V)LCFA triglycerides show great correlation with serum cholesterol, while MCFA triglycerides and most lysophospholipids and free fatty acids ("one-chained" fatty acid molecules) vary more independently. Notation of fatty acid is *"sum of carbon atoms in the two fatty acids : sum of double bonds in the two fatty acids"*. Some lollipop bars show multiple candy due to e.g. one lysophospholipid and one free fatty acid were detected with identical fatty acid type.

Appendix Tables

Appendix Table S1: List of measured targeted metabolites

Metabolite	Sample	Method	CTR*	Ref
7-alpha-hydroxy-4-cholesten-3-one	Serum	HPLC-MS/MS	-	[S11]
Alpha 1-acid glycoprotein (AGP)	Serum	ELISA	-	[S12]
Avenasterol	Serum	GLC	-	[S13,S14]
Campesterol	Serum	GLC	-	[S13,S14]
Chenodeoxycholic acid	Serum	HPLC-MS/MS	-	[S11]
Cholestanol	Serum	GLC	-	[S13,S14]
Cholestenol	Serum	GLC	-	[S14]
Cholesterol, total	Plasma	-	$\leq$ 5.0 mmol/l	[S12], 4515
Cholesterol, HDL	Plasma	-	-	[S12], 4516
Cholesterol, LDL	Plasma	-	$\leq$ 3.0 mmol/l	[S12], 4599
Cholic acid	Serum	HPLC-MS/MS	-	[S11]
C-reactive protein (CRP)	Serum	ELISA	$\leq$ 5 mg/l	[S12]
Creatinine	Urine	-	-	[S12], 2145
Deoxycholic acid	Serum	HPLC-MS/MS	-	[S11]
Desmosterol	Serum	GLC	-	[S14]
Erythrocytes (Red blood cells)	Whole blood	-	-	[S12], 2475
Ferritin	Serum	ELISA	$\geq$ 15 ug/l	[S12]
Glucose (fasting)	Plasma	-	-	[S12], 1468
Glycochenodeoxycholic acid	Serum	HPLC-MS/MS	-	[S11]
Glycocholic acid	Serum	HPLC-MS/MS	-	[S11]
Glycodeoxycholic acid	Serum	HPLC-MS/MS	-	[S11]
Glycolithocholic acid	Serum	HPLC-MS/MS	-	[S11]
Glycoursodeoxycholic acid	Serum	HPLC-MS/MS	-	[S11]
Hematocrit	Whole blood	-	-	[S12], 2475
Hemoglobin	Whole blood	-	$\geq$ 112 mg/l	[S12], 2475
Hyodeoxycholic acid	Serum	HPLC-MS/MS	-	[S11]
Iodine	Urine	IPC-MS	-	[S17], 1984
Lathosterol	Serum	GLC	-	[S14]
Leukocytes (white blood cells)	Whole blood	-	-	[S16], 2475
Lithocholic acid	Serum	HPLC-MS/MS	-	[S11]
Mean corpuscular hemoglobin (MCH)	Whole blood	-	-	[S12], 2475
Mean corpuscular hemoglobin concentration (MCHC)	Whole blood	-	-	[S16], 2475
Mean corpuscular volume (MCV)	Whole blood	-	-	[S16], 2475
Retinol-Binding Protein (RBP)	Serum	ELISA	$\geq$ 1.17 umol/l	[S12]
Red blood cell Distribution Width (RDW)	Whole blood	-	-	[S16], 2475
Sitosterol	Serum	GLC	-	[S13,S14]
Squalene	Serum	GLC	-	[S14]
Taurochenodeoxycholic acid	Serum	HPLC-MS/MS	-	[S11]
Taurocholic acid	Serum	HPLC-MS/MS	-	[S11]
Taurodeoxycholic acid	Serum	HPLC-MS/MS	-	[S11]
Taurolithocholic acid	Serum	HPLC-MS/MS	-	[S11]
Tauroursodeoxycholic acid	Serum	HPLC-MS/MS	-	[S11]
Transferrin Receptor (TfR)	Serum	ELISA	$\geq$ 8.3 mg/l	[S12]
Transthyretin	Serum	-	-	[S16], 2494
Trombocytes	Whole Blood	-	-	[S16], 2475
Triglycerides	Plasma	-	-	[S16], 4568
Ursodeoxycholic acid	Serum	HPLC-MS/MS	-	[S11]
Vitamin B9 (folate)	Erythrocytes	ICMA	-	[S16], 1414
Vitamin B12 (bound to TC2)	Serum	ICMA	$\geq$ 70 pmol/l	[S16], 1142
Vitamin D2-25-OH	Serum	HPLC	-	[S16], 9476
Vitamin D3-25-OH	Serum	HPLC	$\geq$ 50 nmol/l	[S16], 9476
Zinc	Serum	AAS	-	[S16], 2639

*CTR = Clinical target range for children, if assessed, based on the laboratory handbook of the method. Age-adjusted reference range can be found from reference of each method

Appendix Table S2: Pathway analysis results

Appendix Table S2: Results of the pathway analysis showing a list of pathways included in the analysis, total amount of metabolites in each pathway, amount of found metabolites by the untargeted metabolomics method and significant changes to positive (concentration in vegans > concentration in omnivores) or negative direction with corresponding p-values and Enrichment Score (defined in Supplement 3c).

	Metabolites		*Sig. changes**			*p-value***		
**Pathway name**	**Total**	**Found**	**Pos.**	**Neg.**	**Pos.**	**Neg.**	**Any**	**Enrichment Score**
Alanine Metabolism	7	5	1	0	0·56	1·00	0·78	0·2596
Alpha Linolenic Acid and Linoleic Acid Metabolism	17	5	2	1	0·17	0·45	0·12	0·8576
Amino Sugar Metabolism	29	8	0	1	1·00	0·62	0·91	0·2778
Ammonia Recycling	24	10	1	1	0·81	0·70	0·78	0·1697
Androgen and Estrogen Metabolism	17	2	0	0	1·00	1·00	1·00	0·3735
Arachidonic Acid Metabolism	59	4	1	0	0·48	1·00	0·71	0·4927
Arginine and Proline Metabolism	26	10	2	1	0·46	0·70	0·52	0·6791
Aspartate Metabolism	12	5	0	1	1·00	0·45	0·78	0·2064
Beta Oxidation of Very Long Chain Fatty Acids	14	4	0	2	1·00	0·067	0·29	1·6513
Beta-Alanine Metabolism	13	6	0	1	1·00	0·51	0·84	0·3931
Betaine Metabolism	10	3	0	1	1·00	0·30	0·60	0·3495
Bile Acid Biosynthesis	49	13	7	1	0·0015	0·79	0·0085	3·0464
Biotin Metabolism	4	2	0	0	1·00	1·00	1·00	0·3194
Butyrate Metabolism	9	1	0	0	1·00	1·00	1·00	0·1451
Caffeine Metabolism	12	2	0	1	1·00	0·21	0·46	0·6517
Carnitine Synthesis	17	3	1	0	0·39	1·00	0·60	0·2555
Catecholamine Biosynthesis	15	3	0	0	1·00	1·00	1·00	0·2036
Citric Acid Cycle	23	6	0	0	1·00	1·00	1·00	0·1766
Cysteine Metabolism	8	3	0	0	1·00	1·00	1·00	0·0994
D-Arginine and D-Ornithine Metabolism	9	3	1	0	0·39	1·00	0·60	0·3181
DNA Replication Fork	4	1	0	0	1·00	1·00	1·00	0·0471
Degradation of Superoxides	5	0	0	0	1·00	1·00	1·00	0·0000
Ethanol Degradation	15	2	0	1	1·00	0·21	0·46	0·2177
Fatty Acid Biosynthesis	32	21	2	7	0·85	0·0073	0·079	4·5971
Fatty Acid Elongation In Mitochondria	26	1	0	0	1·00	1·00	1·00	0·0604
Fatty acid Metabolism	29	1	0	0	1·00	1·00	1·00	0·1451
Folate Metabolism	22	2	0	0	1·00	1·00	1·00	0·0924
Fructose and Mannose Degradation	18	2	0	0	1·00	1·00	1·00	0·1449
Galactose Metabolism	25	4	0	0	1·00	1·00	1·00	0·1054
Gluconeogenesis	27	6	0	0	1·00	1·00	1·00	0·1456
Glucose-Alanine Cycle	12	5	0	0	1·00	1·00	1·00	0·1342
Glutamate Metabolism	18	5	0	0	1·00	1·00	1·00	0·1473
Glutathione Metabolism	10	5	1	0	0·56	1·00	0·78	0·3815
Glycerol Phosphate Shuttle	8	0	0	0	1·00	1·00	1·00	0·0000
Glycerolipid Metabolism	19	4	0	0	1·00	1·00	1·00	0·1277
Glycine and Serine Metabolism	51	18	2	2	0·78	0·62	0·74	0·4285
Glycolysis	21	3	0	0	1·00	1·00	1·00	0·0706
Histidine Metabolism	24	7	0	1	1·00	0·57	0·89	1·3290
Homocysteine Degradation	6	1	0	0	1·00	1·00	1·00	0·0132
Inositol Metabolism	19	1	0	0	1·00	1·00	1·00	0·0270
Inositol Phosphate Metabolism	20	2	0	0	1·00	1·00	1·00	0·0486
Ketone Body Metabolism	10	3	0	2	1·00	0·036	0·17	1·0003
Lactose Degradation	8	3	0	0	1·00	1·00	1·00	0·0711
Lactose Synthesis	11	3	0	0	1·00	1·00	1·00	0·0711
Lysine Degradation	13	6	1	0	0·63	1·00	0·84	0·3596
Malate-Aspartate Shuttle	8	4	0	1	1·00	0·38	0·71	0·3121
Methionine Metabolism	24	8	1	1	0·73	0·62	0·67	0·2746
Mitochondrial Beta-Oxidation of Long Chain Saturated Fatty Acids	24	1	0	0	1·00	1·00	1·00	0·0471
Mitochondrial Beta-Oxidation of Medium Chain Saturated Fatty Acids	22	2	0	0	1·00	1·00	1·00	0·1133
Mitochondrial Beta-Oxidation of Short Chain Saturated Fatty Acids	24	5	1	1	0·56	0·45	0·40	1·1089

Pathway name	*Metabolites* Total	Found	*Sig. changes** Pos.	Neg.	*p-value*** Pos.	Neg.	Any	Enrichment Score
Mitochondrial Electron Transport Chain	15	2	0	0	1·00	1·00	1·00	0·1747
Nicotinate and Nicotinamide Metabolism	13	1	0	0	1·00	1·00	1·00	0·2114
Nucleotide Sugars Metabolism	9	1	0	0	1·00	1·00	1·00	0·0270
Oxidation of Branched Chain Fatty Acids	14	0	0	0	1·00	1·00	1·00	0·0000
Pantothenate and CoA Biosynthesis	10	2	0	0	1·00	1·00	1·00	0·0245
Pentose Phosphate Pathway	19	1	0	0	1·00	1·00	1·00	0·1295
Phenylacetate Metabolism	4	2	0	0	1·00	1·00	1·00	0·2400
Phenylalanine and Tyrosine Metabolism	13	3	0	0	1·00	1·00	1·00	0·1272
Phosphatidylinositol Phosphate Metabolism	11	2	0	0	1·00	1·00	1·00	0·0486
Phospholipid Biosynthesis	19	2	0	0	1·00	1·00	1·00	0·9672
Phytanic Acid Peroxisomal Oxidation	15	2	0	0	1·00	1·00	1·00	0·0859
Plasmalogen Synthesis	22	2	0	0	1·00	1·00	1·00	1·1284
Porphyrin Metabolism	22	3	2	0	0·062	1·00	0·17	1·1454
Propanoate Metabolism	18	6	0	1	1·00	0·51	0·84	0·5561
Pterine Biosynthesis	25	0	0	0	1·00	1·00	1·00	0·0000
Purine Metabolism	45	8	0	1	1·00	0·62	0·91	0·4741
Pyrimidine Metabolism	36	8	0	0	1·00	1·00	1·00	0·5831
Pyruvaldehyde Degradation	7	4	0	0	1·00	1·00	1·00	0·1213
Pyruvate Metabolism	20	8	0	1	1·00	0·62	0·91	0·6152
Retinol Metabolism	18	2	0	0	1·00	1·00	1·00	0·0469
Riboflavin Metabolism	9	0	0	0	1·00	1·00	1·00	0·0000
Selenoamino Acid Metabolism	15	2	0	0	1·00	1·00	1·00	1·0958
Spermidine and Spermine Biosynthesis	11	3	0	0	1·00	1·00	1·00	0·1704
Sphingolipid Metabolism	15	5	0	1	1·00	0·45	0·78	1·4865
Starch and Sucrose Metabolism	14	2	0	0	1·00	1·00	1·00	0·0438
Steroid Biosynthesis	31	2	1	0	0·28	1·00	0·46	0·1854
Steroidogenesis	33	1	1	0	0·15	1·00	0·26	0·4889
Sulfate/Sulfite Metabolism	7	1	0	0	1·00	1·00	1·00	0·0471
Taurine and Hypotaurine Metabolism	7	2	0	1	1·00	0·21	0·46	0·4234
Thiamine Metabolism	4	1	0	0	1·00	1·00	1·00	0·4144
Threonine and 2-Oxobutanoate Degradation	15	2	0	0	1·00	1·00	1·00	0·0520
Transcription/Translation	28	19	1	3	0·95	0·37	0·78	0·8382
Transfer of Acetyl Groups into Mitochondria	16	5	0	0	1·00	1·00	1·00	0·1161
Trehalose Degradation	9	3	0	0	1·00	1·00	1·00	0·0711
Tryptophan Metabolism	34	6	0	0	1·00	1·00	1·00	0·5240
Tyrosine Metabolism	38	8	0	0	1·00	1·00	1·00	0·2912
Ubiquinone Biosynthesis	10	1	1	0	0·15	1·00	0·26	0·2332
Urea Cycle	20	11	1	1	0·83	0·73	0·83	0·4941
Valine, Leucine and Isoleucine degradation	37	6	0	4	1·00	0·0022	0·046	1·6367
Vitamin B6 Metabolism	10	2	1	1	0·28	0·21	0·070	1·2234
Vitamin K Metabolism	7	0	0	0	1·00	1·00	1·00	0·0000

*Significant change means that there is a fold change of > 0.25 and an uncorrected p-value < 0.05
**p-value is calculated based on Fisher exact test using the amount of significant changes compared to found metabolites

Appendix Table S3: Serum bile acid level sums

Measurement	Omnivore (N = 24)	Vegetarian (N = 10)	Vegan (N = 6)	p-value*
Total cholic acids—μmol/l	0·149 [0·0242–2·26]	0·293 [0·075–2·02]	0·456 [0·185–1·87]	0·089
Total chenodeoxycholic acids—μmol/l	0·55 [0·158–6·18]	1·19 [0·0946–9·94]	1·63 [0·702–5·41]	0·089
Primary bile acids—μmol/l	0·75 [0·182–8·43]	1·58 [0·17–12]	2·08 [0·887–7·27]	0·098
Primary bile acid balance (CDCA per CA)	2·34 [0·553–13·4]	2·14 [0·505–12]	6·32 [1·82–8·2]	0·46
Conjugated primary bile acids—μmol/l	0·568 [0·156–7·89]	0·972 [0·15–11·1]	0·979 [0·432–7·04]	0·42
Taurine-conjugated primary bile acids—μmol/l	0·0974 [0·0246–0·667]	0·0779 [0·0142–0·945]	0·0637 [0·0135–0·316]	0·72
Glycine-conjugated primary bile acids—μmol/l	0·517 [0·107–7·22]	0·894 [0·122–10·7]	0·918 [0·39–6·72]	0·36
Unconjugated primary bile acids—μmol/l	0·15 [0·0266–0·646]	0·244 [0·0195–0·892]	0·498 [0·237–4·05]	0·047
Primary bile acid conjugation ratio—% of all primary bile acids	0·835 [0·469–0·942]	0·883 [0·423–0·989]	0·569 [0·137–0·967]	0·31
PBA conjugation balance (tauro- per glyco-)	0·166 [0·0642–0·448]	0·0954 [0·0386–0·225]	0·0699 [0·0213–0·16]	0·047
Total deoxycholic acids—μmol/l	0·204 [0–1·25]	0·459 [0–2·14]	0·0243 [0–1·62]	0·52
Total lithocholic acids—μmol/l	0·0201 [0–0·0934]	0·0254 [0–0·0609]	0·00076 [0–0·0805]	0·36
Total ursodeoxycholic acids—μmol/l	0·14 [0·00628–4·61]	0·0881 [0·00765–0·648]	0·312 [0·101–1·82]	0·36
Secondary bile acids—μmol/l	0·447 [0·0287–4·62]	0·652 [0·148–2·23]	0·778 [0·101–2·23]	0·57
Conjugated secondary bile acids—μmol/l	0·186 [0·0116–3·87]	0·29 [0·0698–1·91]	0·363 [0·0245–1·64]	0·72
Taurine-conjugated secondary bile acids—μmol/l	0·0171 [0–0·124]	0·0253 [0–0·263]	0·0138 [0–0·0825]	0·79
Glycine-conjugated secondary bile acids—μmol/l	0·17 [0·0116–3·77]	0·271 [0·0698–1·65]	0·323 [0·0245–1·56]	0·68
Unconjugated secondary bile acids—μmol/l	0·191 [0·00245–0·839]	0·269 [0·0783–0·454]	0·415 [0·0581–0·722]	0·60
Secondary bile acid conjugation ratio—% of all secondary bile acids	0·497 [0·167–0·973]	0·499 [0·214–0·871]	0·524 [0·242–0·736]	1·0
SBA conjugation balance (tauro- per glyco-)	0·132 [0–0·447]	0·126 [0–0·28]	0·0301 [0–0·175]	0·089
Total bile acids—μmol/l	1·18 [0·394–13·1]	2·38 [0·796–12·3]	3·23 [1·01–9·62]	0·10
Taurine-conjugated bile acids—μmol/l	0·11 [0·0366–0·765]	0·103 [0·0142–1·07]	0·0775 [0·0135–0·398]	0·68
Glycine-conjugated bile acids—μmol/l	0·674 [0·159–11]	1·24 [0·219–11]	1·5 [0·456–8·28]	0·36
Bile acid conjugation balance (tauro- per glyco-)	0·163 [0·0549–0·394]	0·108 [0·0381–0·249]	0·0517 [0·0205–0·163]	0·047
Bile acid conjugation ratio—% of all bile acids	0·695 [0·266–0·944]	0·631 [0·366–0·956]	0·56 [0·14–0·913]	0·46
Primary bile acids / secondary bile acids	1·96 [0·375–17·9]	2·55 [0·294–36·9]	3·71 [0·846–46·4]	0·42

*p-value is calculated for the comparison between omnivores and vegans using age- and sex-adjusted permutation test.
All values shown as median [min–max].

Appendix Table S4: Amino acids and fatty acids in untargeted metabolome

Metabolite	m/z	Alternative metabolite identities	Difference of means m_{vegan} / $m_{omnivore}$ - 1*	Notes
Glycine	74·02498759	HMDB00123, HMDB14691, HMDB31239	0·256 ± 0·181	
Alanine	88·04039785	HMDB00056, HMDB00161, HMDB00271, HMDB01310, HMDB31219	0·100 ± 0·142	
Serine	104·0353768	HMDB00187, HMDB03406	0·018 ± 0·137	
Proline	114·0562031	HMDB00162, HMDB03411, HMDB12880, HMDB30409, HMDB34208	0·134 ± 0·355	
Valine	116·0715802	HMDB00043, HMDB00883, HMDB01382, HMDB02141, HMDB03355, HMDB13716, HMDB15550	-0·221 ± 0·110	Betaine is another major component under the same peak
Threonine	118·0507756	HMDB00167, HMDB00719, HMDB04041, HMDB61148	-0·034 ± 0·169	
Cysteine	120·0116443	HMDB00574, HMDB03417	0·018 ± 0·152	
Leucine, Isoleucine	130·0871687	HMDB00172, HMDB00557, HMDB00687, HMDB01645, HMDB01901, HMDB03640, HMDB13773, HMDB29168, HMDB33923, HMDB36382, HMDB60650	-0·235 ± 0·121	
Asparagine	131·0459213	HMDB00026, HMDB00168, HMDB11733, HMDB12265, HMDB33780	-0·001 ± 0·162	
Aspartate	132·0298522	HMDB00191, HMDB06483, HMDB11753	-0·174 ± 0·165	
Glutamine	145·061322	HMDB00641, HMDB02031, HMDB03423, HMDB06899, HMDB28687	0·099 ± 0·219	
Lysine	145·0979856	HMDB00182, HMDB03405, HMDB12114, HMDB12115	-0·076 ± 0·202	
Glutamate	146·0456443	HMDB00148, HMDB02393, HMDB02931, HMDB03011, HMDB03339, HMDB06556, HMDB33550, HMDB60475	-0·091 ± 0·211	
Methionine	148·043429	HMDB00696, HMDB14997, HMDB33951	0·054 ± 0·239	
Histidine	154·0615445	HMDB00177, HMDB34267	-0·037 ± 0·091	
Phenylalanine	164·0711161	HMDB00159, HMDB01007, HMDB04992, HMDB06044, HMDB33485, HMDB33589, HMDB34169, HMDB59934	-0·118 ± 0·121	
Arginine	173·1037355	HMDB00517, HMDB03416	0·283 ± 0·224	
Tyrosine	180·0657557	HMDB00158, HMDB01119, HMDB02184, HMDB03831, HMDB06050, HMDB59720, HMDB60280	0·014 ± 0·102	
Tryptophan	203·0818546	HMDB00929, HMDB13609, HMDB13840, HMDB14892, HMDB30396, HMDB60533, HMDB60878, HMDB60967	-0·064 ± 0·124	
Alpha-linolenic acid (ALA, FA18:3)	277·2164727	HMDB0001388, HMDB0003073, HMDB0030962, HMDB0030963, HMDB0030964, HMDB01388, HMDB03073, HMDB30962, HMDB30963, HMDB30964	0·458 ± 0·404	Also gamma-linolenic acid is found under the same peak
Docosahexaenoic acid (DHA, FA22:6)	327·2323316	HMDB0002183, HMDB0030053, HMDB0030446, HMDB0035185, HMDB0038908, HMDB02183, HMDB30053, HMDB30446, HMDB35185, HMDB38908	-0·475 ± 0·197	

*Confidence interval length is calculated using Behrens-Fisher approach

Appendix Table S5: Correlation of essential amino acids with MUAC and transthyretin

**Essential amino acid**	**MUAC z-score**		**Transthyretin**	
	Corr*	p-value**	Corr*	p-value**
Histidine	0.06	0.72	-0.01	0.95
(Iso)Leucine	-0.16	0.33	**0.38**	**0.015**
Lysine	**-0.44**	**0.0048**	0.21	0.20
Methionine	0.06	0.72	0.01	0.95
Phenylalanine	-0.11	0.52	-0.08	0.61
Threonine	-0.08	0.64	0.16	0.31
Tryptophan	0.20	0.23	0.05	0.77
Valine (and betaine)	-0.21	0.21	**0.44**	**0.0048**

*Pearson's correlation coefficient
**by t-test for Pearson's correlation coefficient, not adjusted for multiple comparisons

Appendix Table S6: List of metabolites recognized by untargeted metabolomics

			Comparison [vegan] - [omnivore]		
**m/z**	**HMDB ID alternatives**	**Suggested annotation**	**Fold Change (log2)**	**p-value**	**p-value (BH adj.)**
57,03438372	HMDB0001659, HMDB0003366, HMDB0031558, HMDB0031652, HMDB01659, HMDB03366, HMDB31558, HMDB31652	Acetone	-0,261970493	0,041479977	0,164049886
59,01374188	HMDB0000042, HMDB0003344, HMDB00042, HMDB02165, HMDB03344	Acetatic acid	-0,260207921	0,19476729	0,435479683
67,0185461	HMDB0006853, HMDB0013785, HMDB06853, HMDB06945, HMDB13785	Butynal	-0,191757331	0,913285547	0,962980649
69,03480724	HMDB0034233, HMDB0061873, HMDB0061874, HMDB34233, HMDB61873, HMDB61874	Butynol	-0,014075792	0,060327892	0,202905172
71,01391932	HMDB0001167, HMDB0006112, HMDB0031647, HMDB01167, HMDB06112, HMDB31647	Propenoic acid C3:1	0,039010144	0,243633966	0,498706146
73,02977706	HMDB0000237, HMDB0003052, HMDB0003453, HMDB0006458, HMDB0006961, HMDB00237, HMDB0031229, HMDB0031523, HMDB03052, HMDB03453, HMDB06458, HMDB06949, HMDB06961, HMDB31229, HMDB31523	C3:0 (Propionic acid)	-0,144203463	0,025122616	0,116156048
74,02498759	HMDB0000123, HMDB00123, HMDB0014691, HMDB0031239, HMDB14691, HMDB31239	Glycine	0,328571656	0,049944148	0,179616493
75,00907533	HMDB0000115, HMDB00115, HMDB0031608, HMDB03035, HMDB31608	Glycolate	0,128822003	0,99015596	0,99357422
77,0396704	HMDB0001505, HMDB01505	Benzene	-0,169162951	0,561101246	0,759751998
83,01324813	HMDB0032330, HMDB0094691, HMDB32330, HMDB94691	Hydroxybutynal	0,005317975	0,008881361	0,055517278
83,04853138	HMDB0001184, HMDB0012157, HMDB0031407, HMDB0031512, HMDB0031530, HMDB0031601, HMDB0031606, HMDB0031607, HMDB0032458, HMDB0037788, HMDB0040584, HMDB01184, HMDB12157, HMDB31407, HMDB31512, HMDB31530, HMDB31601, HMDB31606, HMDB31607, HMDB32458, HMDB37788, HMDB40584	Methyl propenyl ketone	0,158031951	0,140497509	0,361397723
85,02983961	HMDB0000549, HMDB0002523, HMDB0003407, HMDB0010720, HMDB0031209, HMDB0033977, HMDB0034439, HMDB00549, HMDB02523, HMDB03407, HMDB10720, HMDB31209, HMDB33977, HMDB34439	Succinic aldehyde	0,016142108	0,002618057	0,02284884
87,00900705	HMDB0000243, HMDB0011111, HMDB00243, HMDB0040261, HMDB0062676, HMDB11111, HMDB11666, HMDB40261, HMDB62676	Pyruvate	0,054294652	0,226967723	0,474056558
88,04039785	HMDB0000056, HMDB0000161, HMDB0000271, HMDB0001310, HMDB00056, HMDB00161, HMDB00271, HMDB0031219, HMDB0062251, HMDB01310, HMDB31219, HMDB62251	Alanine	0,137746726	0,163317507	0,39192014
89,02441532	HMDB0000190, HMDB0000700, HMDB0001051, HMDB0001311, HMDB0001882, HMDB00171, HMDB00190, HMDB0029580, HMDB0031232, HMDB0041929, HMDB0062492, HMDB00700, HMDB01051, HMDB01311, HMDB0144295, HMDB01882, HMDB03328, HMDB29580, HMDB31232, HMDB41929, HMDB62492	Lactate	-0,109148618	0,193150353	0,434090483
91,04019269	HMDB0000131, HMDB00131	Glycerol	-0,085712366	0,117267973	0,314638992
95,01352128	HMDB0032914, HMDB0060503, HMDB32914, HMDB60503	Furfural	0,045671925	0,108797979	0,296474493
102,0563395	HMDB0000092, HMDB0000112, HMDB0000452, HMDB0000650, HMDB0001906, HMDB0002166, HMDB0002299, HMDB0003911, HMDB00092, HMDB00112, HMDB0031329, HMDB0031654, HMDB0038394, HMDB0041945, HMDB00452, HMDB00485, HMDB00581, HMDB00650, HMDB0094692, HMDB01906, HMDB02166, HMDB02299, HMDB03911, HMDB05815, HMDB31329, HMDB31654, HMDB38394, HMDB41945, HMDB94692	Aminobutanoic acid (ABA)	-0,115962132	0,552154738	0,757042346
103,0400697	HMDB0000008, HMDB0000011, HMDB0000023, HMDB0000336, HMDB0000357, HMDB0000442, HMDB0000710, HMDB0000729, HMDB00008, HMDB00011, HMDB00023, HMDB0031212, HMDB00336, HMDB00357, HMDB00435, HMDB00442, HMDB0059748, HMDB00710, HMDB00729, HMDB00758, HMDB0126088, HMDB11597, HMDB31212, HMDB59748	Hydroxybutanoic acid	-0,771260478	0,043011394	0,165955466
104,0353768	HMDB0000187, HMDB0003406, HMDB00187, HMDB00589, HMDB0062263, HMDB03406, HMDB62263	Serine	0,025370129	0,341457632	0,602734929
105,0194147	HMDB0000139, HMDB0006372, HMDB00139, HMDB0031818, HMDB06372, HMDB31818	D-Glyceric acid	0,040995689	0,693655637	0,844166993
105,0337329	HMDB0006115, HMDB06115	Aromatic aldehyde	-0,037614384	0,212269501	0,458862942
107,0363561	HMDB0034260, HMDB34260	2,2-Dihydroperoxypropane	-0,186460199	0,28384517	0,546386287
107,0502731	HMDB0001858, HMDB0002048, HMDB0002055, HMDB0003119, HMDB0033895, HMDB01858, HMDB02048, HMDB02055, HMDB03119, HMDB13762, HMDB33895	Benzyl alcohol	-0,554666822	0,223355571	0,473219052
108,0458755	HMDB0001169, HMDB0035882, HMDB0040582, HMDB01169, HMDB35882, HMDB40582	Aminophenol	0,30703516	0,409585657	0,651952668
109,029373	HMDB0000957, HMDB0002434, HMDB0031180, HMDB0032037, HMDB0033002, HMDB0033127, HMDB00957, HMDB02434, HMDB31180, HMDB32037, HMDB33002, HMDB33127	Catechol	0,343372377	0,445812269	0,680478358
109,0396647	HMDB0003905, HMDB0033156, HMDB0060741, HMDB03905, HMDB06237, HMDB33156, HMDB60741	N-Acetylimidazole	0,244245941	0,325970343	0,58972228
110,0251315	HMDB0004230, HMDB0038175, HMDB03094, HMDB04230, HMDB38175	Pyrrole-2-carboxylic acid	0,068140153	0,182159084	0,417740595
110,0723083	HMDB0000870, HMDB0014417, HMDB0060263, HMDB00870, HMDB14417, HMDB60263	Histamine	-0,081319304	0,486186777	0,710703477
110,9916883	HMDB0029717, HMDB29717	2-Thiophenecarboxaldehyc	-0,008659459	0,003920246	0,030796891
111,008269	HMDB0000444, HMDB0000617, HMDB0032994, HMDB0039437, HMDB00444, HMDB00617, HMDB32994, HMDB39437	Furoic acid	-0,063755294	0,024751175	0,116037769
111,0195255	HMDB0000300, HMDB00300, HMDB0060760, HMDB60760	Uracil	-0,216633125	0,216703787	0,466581982
111,081258	HMDB0031483, HMDB0031486, HMDB0031487, HMDB0031488, HMDB0031539, HMDB0031540, HMDB0031550, HMDB0031551, HMDB0032301, HMDB0033667, HMDB0033827, HMDB0034372, HMDB0040339, HMDB31483, HMDB31486, HMDB31487, HMDB31488, HMDB31539, HMDB31540, HMDB31550, HMDB31551, HMDB32301, HMDB33667, HMDB33827, HMDB34372, HMDB40339	(Z)-4-Heptenal	-0,589165064	1,88913E-06	6,59001E-05

m/z	HMDB ID alternatives	Suggested annotation	Comparison [vegan] - [omnivore] Fold Change (log2)	p-value	p-value (BH adj.)
112,0518513	HMDB0000562, HMDB00562	Creatinine	-0,006966934	0,971120169	0,985816982
112,9998603	HMDB0062802, HMDB62802	Parabanic Acid	-0,040828885	0,719088965	0,860243607
113,0246394	HMDB0031859, HMDB0060461, HMDB31859, HMDB60461	2-Hydroxy-2,4-pentadienoic acid	0,014180757	0,716300685	0,860243607
113,0972785	HMDB0003671, HMDB0004814, HMDB0005841, HMDB0031405, HMDB0031429, HMDB0031475, HMDB0031482, HMDB0031538, HMDB0031549, HMDB0032405, HMDB0038052, HMDB0040191, HMDB0041320, HMDB0059693, HMDB0061896, HMDB03671, HMDB04814, HMDB05841, HMDB31405, HMDB31429, HMDB31475, HMDB31482, HMDB31538, HMDB31549, HMDB32405, HMDB38052, HMDB40191, HMDB41320, HMDB59693, HMDB61896	Heptanone	0,2444565	0,000996689	0,011588167
114,0381225	HMDB0031328, HMDB0032345, HMDB0040331, HMDB31328, HMDB32345, HMDB40331	1-Isothiocyanatobutane	-0,005037652	0,349945062	0,609086016
114,0562031	HMDB0000162, HMDB0003411, HMDB0012880, HMDB00162, HMDB0030409, HMDB0034208, HMDB03411, HMDB12880, HMDB30409, HMDB34208	Proline	0,181360607	0,26060143	0,518822937
115,0033984	HMDB0000134, HMDB0000176, HMDB00134, HMDB00176	Fumarate	0,213877272	0,241934154	0,496391958
115,0399639	HMDB0000019, HMDB0000310, HMDB0000720, HMDB0001865, HMDB00019, HMDB0003771, HMDB0012233, HMDB00310, HMDB0031643, HMDB0034466, HMDB00720, HMDB0128916, HMDB0128917, HMDB0128943, HMDB0129096, HMDB0129097, HMDB0129098, HMDB0132477, HMDB01865, HMDB03771, HMDB04260, HMDB12233, HMDB31643, HMDB34466	Ketovaline	-0,301514837	0,003750264	0,029729369
115,058744	HMDB0032411, HMDB0040244, HMDB32411, HMDB40244	2-Methyl-1-methylthio-2-butene	0,12284778	0,74991051	0,872880287
115,0764773	HMDB0000535, HMDB0000689, HMDB0029762, HMDB0030027, HMDB0030056, HMDB0030059, HMDB0031207, HMDB0031221, HMDB0031246, HMDB0031248, HMDB0031325, HMDB0031511, HMDB0031580, HMDB0033774, HMDB0033889, HMDB0034163, HMDB0037266, HMDB0040279, HMDB0040327, HMDB00535, HMDB00689, HMDB29762, HMDB30027, HMDB30056, HMDB30059, HMDB31207, HMDB31221, HMDB31246, HMDB31248, HMDB31325, HMDB31511, HMDB31580, HMDB33774, HMDB33889, HMDB34163, HMDB37266, HMDB40279, HMDB40327	Hexanoic acid	-0,05651124	0,034846369	0,146086702
116,0352396	HMDB0000532, HMDB0006454, HMDB0012249, HMDB00532, HMDB06454, HMDB12249	Aspartate semialdehyde	-0,309745308	0,016613314	0,087798848
116,0503096	HMDB0000738, HMDB0034171, HMDB00738, HMDB34171	Indole	0,052730753	0,981038995	0,992419958
116,0715802	HMDB0000043, HMDB0000883, HMDB0001382, HMDB0002141, HMDB0003355, HMDB00043, HMDB0013716, HMDB0015550, HMDB00883, HMDB01382, HMDB02141, HMDB03355, HMDB13716, HMDB15550, HMDB34366	Valine, Betaine	-0,36046302	0,002591371	0,02284884
117,0555408	HMDB0000351, HMDB0000354, HMDB0000396, HMDB0000407, HMDB0000410, HMDB0000531, HMDB0000642, HMDB0000754, HMDB0001863, HMDB0001987, HMDB0002011, HMDB00351, HMDB00354, HMDB00396, HMDB00407, HMDB0040735, HMDB00410, HMDB0041603, HMDB00420, HMDB00531, HMDB0059844, HMDB00605, HMDB0061927, HMDB0062584, HMDB00642, HMDB00754, HMDB0130836, HMDB01863, HMDB01987, HMDB02011, HMDB02187, HMDB02321, HMDB40735, HMDB41603, HMDB59844, HMDB61927, HMDB62584	5-Hydroxypentanoic acid	-0,427088687	0,006071676	0,041385986
117,0749191	HMDB0029579, HMDB0031431, HMDB0039806, HMDB29579, HMDB31431, HMDB39806	Diisopropyl sulfide	-0,357483545	0,002323213	0,021102518
118,0507756	HMDB0000167, HMDB0000719, HMDB0004041, HMDB00167, HMDB0061148, HMDB0061877, HMDB00719, HMDB04041, HMDB61148, HMDB61877	Threonine	-0,049502965	0,594416494	0,766762104
119,0350988	HMDB0000337, HMDB0000360, HMDB0000498, HMDB0002453, HMDB0002601, HMDB0002649, HMDB0006293, HMDB00337, HMDB00360, HMDB00498, HMDB02453, HMDB02601, HMDB02649, HMDB06118, HMDB06293	Tetrose	-0,095567099	0,308357927	0,56847381
119,0501843	HMDB0004072, HMDB0006236, HMDB0013815, HMDB0029636, HMDB0029637, HMDB0029638, HMDB0033910, HMDB0038997, HMDB0062765, HMDB0124943, HMDB04072, HMDB06236, HMDB13815, HMDB29636, HMDB29637, HMDB29638, HMDB33910, HMDB38997, HMDB62765	Phenylacetaldehyde	0,348822151	0,028505436	0,126820105
120,0116443	HMDB0000574, HMDB0003417, HMDB00574, HMDB03417	Cys	0,025544419	0,675060796	0,831430811
120,0450635	HMDB0004461, HMDB0033131, HMDB0035281, HMDB04461, HMDB33131, HMDB35281	Benzamide	0,150362048	0,772766528	0,885482802
121,0297413	HMDB0001870, HMDB0011718, HMDB0032918, HMDB0034170, HMDB01870, HMDB11718, HMDB32918, HMDB34170	Benzoate	-0,006037973	0,888450735	0,954337611
121,0660083	HMDB0029306, HMDB0030540, HMDB0032074, HMDB0032076, HMDB0032148, HMDB0032150, HMDB0032151, HMDB0032619, HMDB0033944, HMDB0041609, HMDB0059873, HMDB29306, HMDB30540, HMDB32074, HMDB32076, HMDB32148, HMDB32150, HMDB32151, HMDB32619, HMDB33944, HMDB41609, HMDB59873	Phenylethyl alcohol	0,147343181	0,921306244	0,966761787
121,1025331	HMDB0038140, HMDB0061784, HMDB38140, HMDB61784	Santene	1,019676189	0,004096275	0,031307269
124,0076111	HMDB0000251, HMDB00251	Taurine	-0,452509815	0,005203949	0,038133139
126,0028012	HMDB0032964, HMDB32964	2-Acetylthiazole	-0,478555665	0,004613137	0,034381671
126,0192196	HMDB0034368, HMDB34368	2,3,6-Trihydroxypyridine	-0,101341057	0,248215325	0,50335759
126,9053791	HMDB0059634, HMDB59634	I(-)	0,306178856	0,736718055	0,867582504
127,0033099	HMDB0059784, HMDB59784	5-Hydroxy-2-furoic acid	0,002760004	0,968357421	0,985306501
127,0141638	HMDB0041833, HMDB41833	Barbiturate	0,064246657	0,376960033	0,628506977
127,0514138	HMDB0000079, HMDB00079, HMDB0029874, HMDB0031547, HMDB0062558, HMDB29874, HMDB31547, HMDB62558	Dihydrothymine	0,146386784	0,471717782	0,700188213
128,0102858	HMDB0041861, HMDB41861	Cyanuric acid	-0,051306049	0,001320782	0,014218788
128,0350019	HMDB0000267, HMDB0000805, HMDB0001369, HMDB0001843, HMDB0002234, HMDB00267, HMDB0060262, HMDB0061093, HMDB0062585, HMDB00805, HMDB01369, HMDB01843, HMDB02234, HMDB60262, HMDB61093, HMDB62585	Oxoproline	0,026010978	0,410139685	0,651952668

m/z	HMDB ID alternatives	Suggested annotation	Comparison [vegan] - [omnivore] Fold Change (log2)	p-value	p-value (BH adj.)
128,0710005	HMDB0000070, HMDB0000716, HMDB0004226, HMDB0005960, HMDB00070, HMDB0015212, HMDB0029435, HMDB0029444, HMDB0059649, HMDB0062225, HMDB00716, HMDB0094696, HMDB0094697, HMDB04226, HMDB05960, HMDB15212, HMDB29435, HMDB29444, HMDB59649, HMDB62225, HMDB94696, HMDB94697	Pipecolate	0,451945857	5,11817E-05	0,000976502
129,0192608	HMDB0000620, HMDB0000634, HMDB0000749, HMDB0002092, HMDB0013233, HMDB0033809, HMDB0059743, HMDB0060365, HMDB0061185, HMDB00620, HMDB00634, HMDB00749, HMDB01657, HMDB02092, HMDB13233, HMDB33809, HMDB59743, HMDB60365, HMDB61185	Itaconate	0,037326516	0,010707244	0,063085924
129,0386557	HMDB0031370, HMDB0037493, HMDB0040238, HMDB31370, HMDB37493, HMDB40238	2-[(Methylthio)methyl]-2-butenal	-0,116806177	0,910423618	0,962636298
129,0902986	HMDB0000666, HMDB0030028, HMDB0030052, HMDB0031528, HMDB0031594, HMDB0031595, HMDB0031600, HMDB0032204, HMDB0032874, HMDB0033745, HMDB0034166, HMDB0034239, HMDB0035238, HMDB0036209, HMDB0036237, HMDB0036238, HMDB0038227, HMDB0039095, HMDB0039618, HMDB0039619, HMDB0040297, HMDB00666, HMDB30028, HMDB30052, HMDB31528, HMDB31594, HMDB31595, HMDB31600, HMDB32204, HMDB32874, HMDB33745, HMDB34166, HMDB34239, HMDB35238, HMDB36209, HMDB36237, HMDB36238, HMDB38227, HMDB39095, HMDB39618, HMDB39619, HMDB40297	Ethyl isovalerate	-0,140694289	0,045045794	0,168598232
129,1039234	HMDB0002064, HMDB0060268, HMDB02064, HMDB60268	N-Acetylputrescine	-0,065928229	0,064596254	0,209979201
130,051576	HMDB0000725, HMDB0000766, HMDB0000783, HMDB0001149, HMDB0002104, HMDB0002113, HMDB0006055, HMDB0006272, HMDB0036576, HMDB0059659, HMDB0060460, HMDB0061880, HMDB00725, HMDB00766, HMDB00783, HMDB01149, HMDB02104, HMDB02113, HMDB0240251, HMDB06055, HMDB06272, HMDB36576, HMDB59659, HMDB60460, HMDB61880	5-Aminolevulinic acid	-0,08385991	0,179459013	0,416192178
130,0871687	HMDB0000172, HMDB0000557, HMDB0000687, HMDB0001645, HMDB0001901, HMDB0003640, HMDB0013773, HMDB00172, HMDB0029168, HMDB0033923, HMDB0036382, HMDB00557, HMDB0060650, HMDB0061716, HMDB0062203, HMDB00687, HMDB01645, HMDB01901, HMDB02002, HMDB02291, HMDB03640, HMDB13773, HMDB29168, HMDB33923, HMDB36382, HMDB60650, HMDB61716, HMDB62203	(Iso)Leucine	-0,388966263	0,003019489	0,025563055
131,035504	HMDB0000576, HMDB0000622, HMDB0000661, HMDB0001844, HMDB0002001, HMDB0006833, HMDB0006855, HMDB0029884, HMDB0033958, HMDB0040531, HMDB00576, HMDB0061879, HMDB00622, HMDB00661, HMDB01844, HMDB02001, HMDB06833, HMDB06855, HMDB29884, HMDB33958, HMDB40531, HMDB61879	Glutarate	0,036685735	0,457571664	0,691905798
131,0459213	HMDB0000026, HMDB0000168, HMDB00026, HMDB0011733, HMDB0012265, HMDB00168, HMDB0033780, HMDB11733, HMDB12265, HMDB33780	Asparagine	-0,001848173	0,84165977	0,923179018
131,0716322	HMDB0000317, HMDB0000409, HMDB0000525, HMDB0000624, HMDB0000665, HMDB0000746, HMDB0001624, HMDB0001975, HMDB0010718, HMDB0012843, HMDB0029166, HMDB00317, HMDB0032158, HMDB0032456, HMDB0033851, HMDB0040409, HMDB00409, HMDB0041844, HMDB00525, HMDB0059770, HMDB0061652, HMDB00624, HMDB00665, HMDB00746, HMDB01624, HMDB01975, HMDB10718, HMDB12843, HMDB29166, HMDB32158, HMDB32456, HMDB33851, HMDB40409, HMDB41844, HMDB59770, HMDB61652	D-2-Hydroxyisocaproate	-0,005249042	0,838810633	0,921212686
131,0828955	HMDB0000214, HMDB0003374, HMDB00214, HMDB00818, HMDB03374, HMDB04984	Ornithine	0,136067459	0,51537644	0,730874126
132,0298522	HMDB0000191, HMDB0006483, HMDB0011753, HMDB00191, HMDB0062186, HMDB06483, HMDB11753, HMDB62186	Aspartate	-0,276252997	0,93531185	0,975689772
132,0451062	HMDB0004094, HMDB0029757, HMDB0032390, HMDB0034666, HMDB0059805, HMDB0060486, HMDB0061918, HMDB04094, HMDB29757, HMDB32390, HMDB34666, HMDB59805, HMDB60486, HMDB61918	Mandelonitrile	-0,193246837	0,042515918	0,1647728
132,0662072	HMDB0031658, HMDB0032356, HMDB0039499, HMDB31658, HMDB32356, HMDB39499	Hydroxy-valine	0,364370804	0,156575411	0,382286785
133,0134686	HMDB0000156, HMDB0000744, HMDB00156, HMDB0031518, HMDB0032872, HMDB00744, HMDB31518, HMDB32872	Malate	0,004741406	0,047929661	0,174912962
133,0277067	HMDB0032469, HMDB0061916, HMDB0129378, HMDB32469, HMDB61916	Phthalide	0,052917972	0,107397883	0,295428876
133,0504291	HMDB0000421, HMDB0001292, HMDB0003224, HMDB0012141, HMDB0012485, HMDB0029576, HMDB00421, HMDB0127615, HMDB01292, HMDB03224, HMDB12141, HMDB12485, HMDB29576	Deoxyribose	0,568375853	0,048141182	0,174912962
133,0657126	HMDB0029639, HMDB0029697, HMDB0029698, HMDB0030839, HMDB0031626, HMDB0032024, HMDB0032142, HMDB0032608, HMDB0032623, HMDB0033716, HMDB0034107, HMDB0034563, HMDB0036441, HMDB0059601, HMDB0059895, HMDB29639, HMDB29697, HMDB29698, HMDB30839, HMDB31626, HMDB32024, HMDB32142, HMDB32608, HMDB32623, HMDB33716, HMDB34107, HMDB34563, HMDB36441, HMDB59601, HMDB59895	Indan-1-ol	0,53872027	0,393884561	0,641527233
134,0051634	HMDB0032930, HMDB32930	Benzothiazole	0,024454281	0,50707781	0,727256333
134,0231899	HMDB0032931, HMDB32931	2-Benzoxazolol	0,013676696	0,815187762	0,911338113
134,0472032	HMDB0000034, HMDB00034	Adenine	-0,030272207	0,404599428	0,647359085
134,0613167	HMDB0001250, HMDB0010715, HMDB0032628, HMDB0032630, HMDB0037071, HMDB0037072, HMDB0040008, HMDB0040013, HMDB0040222, HMDB0040223, HMDB0040224, HMDB01250, HMDB10715, HMDB32628, HMDB32630, HMDB37071, HMDB37072, HMDB40008, HMDB40013, HMDB40222, HMDB40223, HMDB40224	2-Phenylacetamide	-0,722370264	0,068354751	0,214705473
135,0305049	HMDB0000613, HMDB0000943, HMDB00613, HMDB0062620, HMDB00943, HMDB62620	Threonate	0,147094674	0,7352115	0,867529672
135,0305049	HMDB0031160, HMDB0033053, HMDB0033054, HMDB31160, HMDB33053, HMDB33054	1-Pentanesulfenothioic acid	0,147094674	0,7352115	0,867529672
135,0305049	HMDB0000157, HMDB0014581, HMDB00157, HMDB14581	Hypoxanthine	0,147094674	0,7352115	0,867529672

m/z	HMDB ID alternatives	Suggested annotation	Comparison [vegan] - [omnivore] Fold Change (log2)	p-value	p-value (BH adj.)
135,0809615	HMDB0029667, HMDB0031312, HMDB0031446, HMDB0031627, HMDB0031628, HMDB0032029, HMDB0032149, HMDB0032441, HMDB0032544, HMDB0032621, HMDB0032624, HMDB0032625, HMDB0033962, HMDB0034378, HMDB0038984, HMDB0038985, HMDB0038986, HMDB0038987, HMDB0038988, HMDB0039782, HMDB0059896, HMDB0125105, HMDB29667, HMDB31312, HMDB31446, HMDB31627, HMDB31628, HMDB32029, HMDB32149, HMDB32441, HMDB32544, HMDB32621, HMDB32624, HMDB32625, HMDB33962, HMDB34378, HMDB38984, HMDB38985, HMDB38986, HMDB38987, HMDB38988, HMDB39782, HMDB59896	4-Propylphenol	0,066110224	0,794479319	0,897391148
141,1274613	HMDB0013821, HMDB0029557, HMDB0030959, HMDB0031266, HMDB0031267, HMDB0031268, HMDB0031417, HMDB0031684, HMDB0031730, HMDB0034152, HMDB0037304, HMDB0040581, HMDB0041498, HMDB0041499, HMDB0041601, HMDB0059835, HMDB0059887, HMDB13821, HMDB13822, HMDB29557, HMDB30959, HMDB31266, HMDB31267, HMDB31268, HMDB31417, HMDB31684, HMDB31730, HMDB34152, HMDB37304, HMDB40581, HMDB41498, HMDB41499, HMDB41601, HMDB59835, HMDB59887	2-Methyl-4-heptanone	0,102037682	0,29144772	0,553687172
142,0505534	HMDB0000894, HMDB0014491, HMDB0032161, HMDB0061082, HMDB0061705, HMDB00894, HMDB06723, HMDB06724, HMDB14491, HMDB32161, HMDB61082, HMDB61705	2-Hydroxymethylclavam	0,093145139	0,210055202	0,455642129
142,0660756	HMDB0033115, HMDB0041802, HMDB0042004, HMDB33115, HMDB41802, HMDB42004	6-Methylquinoline	1,082353629	0,010663842	0,063085924
142,9982082	HMDB0062781, HMDB62781	2-oxoglutarate(2-)	0,086641029	0,003967555	0,030890252
143,0334447	HMDB0000393, HMDB0000522, HMDB0002266, HMDB0013311, HMDB0029171, HMDB0031257, HMDB0036232, HMDB00393, HMDB0040207, HMDB00522, HMDB02266, HMDB03520, HMDB13311, HMDB29171, HMDB31257, HMDB36232, HMDB40207	2,3-Dimethylmaleate	0,023979982	0,50115572	0,722211629
143,0704383	HMDB0001988, HMDB0031176, HMDB0036594, HMDB0037114, HMDB0040433, HMDB01988, HMDB31176, HMDB36594, HMDB37114, HMDB40433	trans-4-Hydroxycyclohexanecarł	0,374828446	0,077642333	0,234270293
143,1076911	HMDB0000482, HMDB0001877, HMDB0029980, HMDB0030368, HMDB0031230, HMDB0031290, HMDB0031478, HMDB0031579, HMDB0031587, HMDB0031588, HMDB0031596, HMDB0031638, HMDB0031703, HMDB0032046, HMDB0032047, HMDB0032203, HMDB0032225, HMDB0032272, HMDB0032335, HMDB0032551, HMDB0034161, HMDB0034164, HMDB0034234, HMDB0034459, HMDB0035457, HMDB0036165, HMDB0039217, HMDB0039620, HMDB0040209, HMDB0040251, HMDB0040252, HMDB0040296, HMDB0040298, HMDB00482, HMDB0061931, HMDB01877, HMDB29980, HMDB30368, HMDB31230, HMDB31290, HMDB31478, HMDB31579, HMDB31587, HMDB31588, HMDB31596, HMDB31638, HMDB31703, HMDB32046, HMDB32047, HMDB32203, HMDB32225, HMDB32272, HMDB32335, HMDB32551, HMDB34161, HMDB34164, HMDB34234, HMDB34459, HMDB35457, HMDB36165, HMDB39217, HMDB39620, HMDB40209, HMDB40251, HMDB40252, HMDB40296, HMDB40298, HMDB61931	Octanoic acid	-0,837790934	0,012728721	0,072074317
144,0450713	HMDB0029737, HMDB29737	3-Methyleneoxindole	0,056355139	0,146573226	0,371546085
144,0663993	HMDB0000730, HMDB0000808, HMDB0001263, HMDB0003681, HMDB0012131, HMDB0012151, HMDB0015127, HMDB0029425, HMDB0029426, HMDB0059595, HMDB0060265, HMDB0061156, HMDB00730, HMDB00808, HMDB0094698, HMDB01263, HMDB03681, HMDB06238, HMDB12131, HMDB12151, HMDB15127, HMDB29425, HMDB29426, HMDB59595, HMDB60265, HMDB61156, HMDB94698	4-Acetamidobutanoate	0,029609154	0,53506971	0,747725621
144,0799435	HMDB0032967, HMDB32967	2,4-Dimethyl-1H-indole	0,219564288	0,281826833	0,546386287
145,0146434	HMDB0000208, HMDB0013701, HMDB00208, HMDB0062739, HMDB02812, HMDB13701, HMDB62739	Oxoglutarate	0,069890122	0,813182794	0,910263667
145,0146434	HMDB0033966, HMDB0041390, HMDB0041393, HMDB33966, HMDB41390, HMDB41393	Di-2-propenyl disulfide, 9CI	0,069890122	0,813182794	0,910263667
145,061322	HMDB0000641, HMDB0002031, HMDB0003423, HMDB0006899, HMDB0028687, HMDB00641, HMDB02031, HMDB03423, HMDB06899, HMDB28687	Glutamine	0,136449766	0,348838114	0,608373671
145,0979856	HMDB0000182, HMDB0003405, HMDB0012114, HMDB0012115, HMDB00182, HMDB0142894, HMDB03405, HMDB12114, HMDB12115	Lysine	-0,113509189	0,639924091	0,801743976
146,0456443	HMDB0000148, HMDB0002393, HMDB0002931, HMDB0003011, HMDB0003339, HMDB0006556, HMDB00148, HMDB0033550, HMDB0060475, HMDB0062183, HMDB02393, HMDB02931, HMDB03011, HMDB03339, HMDB06556, HMDB33550, HMDB60475, HMDB62183	Glutamate	-0,137740861	0,788778202	0,894427298
146,0808772	HMDB0029449, HMDB0033453, HMDB0061157, HMDB0142963, HMDB29449, HMDB33453, HMDB61157	(2R,3R,4R)-2-Amino-4-hydroxy-3-methylpentanoic acid	0,007753521	0,550580643	0,756072946
147,0297733	HMDB0000426, HMDB0000428, HMDB0000606, HMDB0000694, HMDB0001900, HMDB0011676, HMDB00426, HMDB00428, HMDB0059655, HMDB00606, HMDB00694, HMDB01900, HMDB02323, HMDB11676, HMDB59655	Hydroxyglutarate	-0,065752011	0,041953125	0,164049886
147,0297733	HMDB0033912, HMDB0038890, HMDB0041392, HMDB33912, HMDB38890, HMDB41392	2-Propenyl propyl disulfide	-0,065752011	0,041953125	0,164049886
147,0443588	HMDB0000567, HMDB0000930, HMDB0031725, HMDB0032947, HMDB0035243, HMDB0036626, HMDB0040986, HMDB00567, HMDB00930, HMDB0128078, HMDB0134039, HMDB0135273, HMDB0135274, HMDB0135275, HMDB0135648, HMDB31725, HMDB32947, HMDB35243, HMDB36626, HMDB40986	Cinnamate	-0,092885011	0,410461026	0,651952668
147,0655932	HMDB0000227, HMDB0012140, HMDB00227, HMDB0031639, HMDB0059629, HMDB0059705, HMDB12140, HMDB31639, HMDB59629, HMDB59705	Mevalonate	0,262511502	0,032328071	0,139554841

m/z	HMDB ID alternatives	Suggested annotation	Comparison [vegan] - [omnivore]<br>Fold Change (log2)	p-value	p-value (BH adj.)
147,0805264	HMDB0002214, HMDB0030837, HMDB0031616, HMDB0032014, HMDB0032015, HMDB0032016, HMDB0032017, HMDB0032140, HMDB0033382, HMDB0034121, HMDB0059845, HMDB0133593, HMDB0133746, HMDB02214, HMDB30837, HMDB31616, HMDB32014, HMDB32015, HMDB32016, HMDB32017, HMDB32140, HMDB33382, HMDB34121, HMDB59845	Cuminaldehyde	0,099055245	0,642147303	0,803375105
148,043429	HMDB0000696, HMDB0014997, HMDB0033951, HMDB00696, HMDB14997, HMDB33951	Methionine	0,075227317	0,726890679	0,864557648
149,0243906	HMDB0001587, HMDB0031515, HMDB0032598, HMDB0032612, HMDB0040528, HMDB01587, HMDB31515, HMDB32598, HMDB32612, HMDB40528	Phenylglyoxylic acid	0,017852577	0,793534949	0,897391148
149,0456853	HMDB0000098, HMDB0000283, HMDB0000366, HMDB0000621, HMDB0000646, HMDB0000751, HMDB0001644, HMDB0003371, HMDB00098, HMDB0012194, HMDB0012325, HMDB00283, HMDB0029941, HMDB0029942, HMDB00366, HMDB00487, HMDB0059753, HMDB0060254, HMDB00621, HMDB00646, HMDB00654, HMDB00751, HMDB0094694, HMDB0127617, HMDB01644, HMDB02245, HMDB03371, HMDB03763, HMDB12194, HMDB12325, HMDB29941, HMDB29942, HMDB59753, HMDB60254, HMDB94694	Pentose	0,17373324	0,151957447	0,380720112
149,0456853	HMDB0029578, HMDB0031161, HMDB0031472, HMDB0031709, HMDB0032180, HMDB0032408, HMDB0040056, HMDB29578, HMDB31161, HMDB31472, HMDB31709, HMDB32180, HMDB32408, HMDB40056	Diisopropyl disulfide	0,17373324	0,151957447	0,380720112
149,060306	HMDB0000764, HMDB0002097, HMDB0002222, HMDB0002237, HMDB0003654, HMDB0011743, HMDB0013744, HMDB0031310, HMDB0032073, HMDB0032075, HMDB0032569, HMDB0032570, HMDB0032592, HMDB0032617, HMDB0033967, HMDB0033970, HMDB0037715, HMDB0038355, HMDB0040428, HMDB0040925, HMDB0059824, HMDB0062349, HMDB00764, HMDB0125532, HMDB0134026, HMDB0134027, HMDB0135239, HMDB0135276, HMDB0135277, HMDB0135278, HMDB0135279, HMDB0135280, HMDB0135281, HMDB0135644, HMDB02097, HMDB02222, HMDB02237, HMDB03654, HMDB11743, HMDB13744, HMDB31310, HMDB32073, HMDB32075, HMDB32569, HMDB32570, HMDB32592, HMDB32617, HMDB33967, HMDB33970, HMDB37715, HMDB38355, HMDB40428, HMDB40925, HMDB59824, HMDB62349	Hydrocinnamic acid	0,062327786	0,745321344	0,872376123
149,0969442	HMDB0001878, HMDB0003647, HMDB0004487, HMDB0029652, HMDB0031570, HMDB0031613, HMDB0031817, HMDB0032063, HMDB0032213, HMDB0032626, HMDB0033544, HMDB0034846, HMDB0034921, HMDB0035089, HMDB0035172, HMDB0035250, HMDB0035591, HMDB0035761, HMDB0035770, HMDB0035824, HMDB0036061, HMDB0036070, HMDB0036089, HMDB0036999, HMDB0037561, HMDB0038913, HMDB0061788, HMDB0133616, HMDB01878, HMDB03647, HMDB04487, HMDB06315, HMDB29652, HMDB31570, HMDB31613, HMDB31817, HMDB32063, HMDB32213, HMDB32626, HMDB33544, HMDB34846, HMDB34921, HMDB35089, HMDB35172, HMDB35250, HMDB35591, HMDB35761, HMDB35770, HMDB35824, HMDB36061, HMDB36070, HMDB36089, HMDB36999, HMDB37561, HMDB38913, HMDB61788	Thymol	-0,123375755	0,700635242	0,847843971
150,0014886	HMDB0034413, HMDB0040578, HMDB34413, HMDB40578	1,2-Benzisothiazol-3(2H)-one	-0,478232136	0,276371768	0,541564452
150,0562558	HMDB0001859, HMDB0002210, HMDB0012219, HMDB0012992, HMDB0029703, HMDB0032609, HMDB0033903, HMDB0059846, HMDB0060680, HMDB0061919, HMDB01859, HMDB02210, HMDB12219, HMDB12992, HMDB29703, HMDB32609, HMDB33903, HMDB59846, HMDB60680, HMDB61919	Acetaminophen	0,015712596	0,445226471	0,680478358
151,0258056	HMDB0000292, HMDB0000786, HMDB0001182, HMDB00292, HMDB00786, HMDB01182	Xanthine	-0,447599455	3,24778E-05	0,00072617
151,0401079	HMDB0000020, HMDB0000440, HMDB0000669, HMDB0000703, HMDB0001101, HMDB00020, HMDB0002390, HMDB0003791, HMDB0004815, HMDB0012308, HMDB0029657, HMDB0029658, HMDB0029659, HMDB0029660, HMDB0029661, HMDB0029662, HMDB0031609, HMDB0032399, HMDB0032572, HMDB0032594, HMDB0032604, HMDB0032606, HMDB0032629, HMDB0032919, HMDB0033003, HMDB0034172, HMDB0036907, HMDB0041283, HMDB00440, HMDB0060390, HMDB0062635, HMDB00669, HMDB00703, HMDB01101, HMDB0124926, HMDB0125528, HMDB0137457, HMDB02390, HMDB03791, HMDB04815, HMDB06256, HMDB06778, HMDB12308, HMDB29657, HMDB29658, HMDB29659, HMDB29660, HMDB29661, HMDB29662, HMDB31609, HMDB32399, HMDB32572, HMDB32594, HMDB32604, HMDB32606, HMDB32629, HMDB32919, HMDB33003, HMDB34172, HMDB36907, HMDB41283, HMDB60390, HMDB62635	p-Hydroxyphenylacetic acid	-0,16112806	0,058563209	0,198704741
151,0500454	HMDB0004193, HMDB0004194, HMDB04193, HMDB04194	N1-Methyl-2-pyridone-5-carboxamide	0,257121499	0,613835234	0,783695935

m/z	HMDB ID alternatives	Suggested annotation	Comparison [vegan] - [omnivore] Fold Change (log2)	p-value	p-value (BH adj.)
151,112569	HMDB0003450, HMDB0003634, HMDB0003667, HMDB0030892, HMDB0030893, HMDB0031854, HMDB0032120, HMDB0032242, HMDB0032536, HMDB0034715, HMDB0034916, HMDB0034973, HMDB0034974, HMDB0034975, HMDB0034985, HMDB0035054, HMDB0035077, HMDB0035078, HMDB0035092, HMDB0035100, HMDB0035120, HMDB0035125, HMDB0035158, HMDB0035203, HMDB0035241, HMDB0035277, HMDB0035280, HMDB0035600, HMDB0035604, HMDB0035622, HMDB0035656, HMDB0035706, HMDB0035737, HMDB0035738, HMDB0035741, HMDB0035743, HMDB0035744, HMDB0036044, HMDB0036079, HMDB0036080, HMDB0036082, HMDB0036083, HMDB0036085, HMDB0036087, HMDB0036088, HMDB0036113, HMDB0036114, HMDB0036115, HMDB0036127, HMDB0036128, HMDB0036129, HMDB0036598, HMDB0037008, HMDB0037009, HMDB0037010, HMDB0037015, HMDB0037017, HMDB0037024, HMDB0037025, HMDB0037172, HMDB0037280, HMDB0037302, HMDB0037303, HMDB0038251, HMDB0038290, HMDB0038557, HMDB0039008, HMDB0039816, HMDB0040766, HMDB0041011, HMDB0041013, HMDB0041631, HMDB0059608, HMDB0059761, HMDB0059827, HMDB0059838, HMDB0059875, HMDB0059885, HMDB0061794, HMDB03450, HMDB03634, HMDB03667, HMDB06291, HMDB06410, HMDB30892, HMDB30893, HMDB31854, HMDB32120, HMDB32242, HMDB32536, HMDB34715, HMDB34916, HMDB34973, HMDB34974, HMDB34975, HMDB34985, HMDB35054, HMDB35077, HMDB35078, HMDB35092, HMDB35100, HMDB35120, HMDB35125, HMDB35158, HMDB35203, HMDB35241, HMDB35277, HMDB35280, HMDB35600, HMDB35604, HMDB35622, HMDB35656, HMDB35706, HMDB35737, HMDB35738, HMDB35741, HMDB35743, HMDB35744, HMDB36044, HMDB36079, HMDB36080, HMDB36082, HMDB36083, HMDB36085, HMDB36087, HMDB36088, HMDB36113, HMDB36114, HMDB36115, HMDB36127, HMDB36128, HMDB36129, HMDB36598, HMDB37008, HMDB37009, HMDB37010, HMDB37015, HMDB37017, HMDB37024, HMDB37025, HMDB37172, HMDB37280, HMDB37302, HMDB37303, HMDB38251, HMDB38290, HMDB38557, HMDB39008, HMDB39816, HMDB40766, HMDB41011, HMDB41013, HMDB41631, HMDB59608, HMDB59761, HMDB59827, HMDB59838, HMDB59875, HMDB59885, HMDB61794	(-)-trans-Carveol	-0,904351166	0,001318972	0,014218788
152,0344082	HMDB0001476, HMDB0001972, HMDB0014378, HMDB0014389, HMDB0059749, HMDB0059811, HMDB01476, HMDB01972, HMDB14378, HMDB14389, HMDB59749, HMDB59811	3-Hydroxyanthranilic acid	-0,145583013	0,113191538	0,304638953
152,0715673	HMDB0000073, HMDB0004825, HMDB00073, HMDB0012309, HMDB04825, HMDB12309	Dopamine	-0,020905806	0,54050746	0,751710534
153,0023388	HMDB0040002, HMDB40002	1-(2-Thienyl)-1,2-propanedione	0,169505945	0,09274532	0,268684116
153,0192513	HMDB0000152, HMDB0000397, HMDB0001856, HMDB0013676, HMDB0013677, HMDB00152, HMDB0029666, HMDB0034299, HMDB00397, HMDB0125090, HMDB01856, HMDB13676, HMDB13677, HMDB29666, HMDB34299	Gentisic acid	0,948384877	0,062385523	0,204664919
153,0536465	HMDB0005784, HMDB0030570, HMDB0032012, HMDB0034158, HMDB0037724, HMDB0037728, HMDB0059966, HMDB0125534, HMDB0126617, HMDB0131231, HMDB0131232, HMDB0131479, HMDB0135785, HMDB05784, HMDB30570, HMDB32012, HMDB34158, HMDB37724, HMDB37728, HMDB59966	Hydroxytyrosol	0,402203893	0,074183321	0,226181313
153,0701389	HMDB0034437, HMDB0034968, HMDB34437, HMDB34968	Biphenyl	0,266521233	0,182201505	0,417740595
153,0919602	HMDB0031302, HMDB0032314, HMDB0032442, HMDB0035135, HMDB0036177, HMDB0036239, HMDB0037276, HMDB0038268, HMDB0038697, HMDB0040285, HMDB0060285, HMDB31302, HMDB32314, HMDB32442, HMDB35135, HMDB36177, HMDB36239, HMDB37276, HMDB38268, HMDB38697, HMDB40285, HMDB60285	C9:2	0,166996273	0,309721081	0,569306197
154,0615445	HMDB0000177, HMDB00177, HMDB0034267, HMDB03412, HMDB34267	Histidine	-0,05381822	0,355652063	0,614116037
154,9859044	HMDB0041066, HMDB41066	S-Propyl thiosulfate	0,077830931	0,88833261	0,954337611
154,9987004	HMDB0004812, HMDB00578, HMDB04812	2,5-Furandicarboxylic acid	0,050536626	0,353337416	0,612545182
154,9987004	HMDB0040939, HMDB40939	Methyl phenyl disulfide	0,050536626	0,353337416	0,612545182
155,0460469	HMDB0000544, HMDB0001014, HMDB0002320, HMDB00544, HMDB01014, HMDB02320	5-Hydroxymethyl-4-methyluracil	0,066714501	0,244916657	0,499562978
155,1074225	HMDB0004362, HMDB0031263, HMDB0031271, HMDB0031514, HMDB0031531, HMDB0032265, HMDB0032307, HMDB0032413, HMDB0032491, HMDB0033167, HMDB0034716, HMDB0035307, HMDB0036208, HMDB0036222, HMDB0037023, HMDB0037496, HMDB0037618, HMDB0037629, HMDB0038272, HMDB0038275, HMDB0039736, HMDB0039794, HMDB0040210, HMDB0059728, HMDB0059817, HMDB0059840, HMDB0059894, HMDB0060286, HMDB04362, HMDB31263, HMDB31271, HMDB31514, HMDB31531, HMDB32265, HMDB32307, HMDB32413, HMDB32491, HMDB33167, HMDB34716, HMDB35307, HMDB36208, HMDB36222, HMDB37023, HMDB37496, HMDB37618, HMDB37629, HMDB38272, HMDB38275, HMDB39736, HMDB39794, HMDB40210, HMDB59728, HMDB59817, HMDB59840, HMDB59894, HMDB60286	C9:1	0,024866471	0,227786351	0,474056558
156,044297	HMDB0062189, HMDB62189	1-nitrosonaphthalene	0,081304441	0,383912341	0,636331303
156,1028434	HMDB0014447, HMDB0033433, HMDB14447, HMDB33433	Tranexamic Acid	0,003921891	0,553671811	0,757470764
157,0127245	HMDB0031210, HMDB0037759, HMDB0060348, HMDB0137818, HMDB31210, HMDB37759, HMDB60348	Zymonic acid	0,080367449	0,960065526	0,98375692
157,0499276	HMDB0000635, HMDB0012241, HMDB0036380, HMDB0059815, HMDB00635, HMDB12241, HMDB36380, HMDB59815	Isopropylmaleate	-0,040869816	0,282474831	0,546386287
157,0867424	HMDB0000451, HMDB0000909, HMDB0010721, HMDB0013211, HMDB0030303, HMDB0031177, HMDB0031307, HMDB0036230, HMDB0036395, HMDB0038305, HMDB0040447, HMDB0041616, HMDB00451, HMDB0059938, HMDB0059939, HMDB0060683, HMDB0060685, HMDB0062788, HMDB00909, HMDB10721, HMDB13211, HMDB30303, HMDB31177, HMDB31307, HMDB36230, HMDB36395, HMDB38305, HMDB40447, HMDB41616, HMDB59938, HMDB59939, HMDB60683, HMDB60685, HMDB62788	cis-4-Hydroxycyclohexylaceti acid	-0,02997644	0,636015992	0,801639866

m/z	HMDB ID alternatives	Suggested annotation	Comparison [vegan] - [omnivore] Fold Change (log2)	p-value	p-value (BH adj.)
157,1231227	HMDB0000798, HMDB0000847, HMDB0030331, HMDB0031291, HMDB0031480, HMDB0031557, HMDB0032042, HMDB0032309, HMDB0032406, HMDB0032454, HMDB0034162, HMDB0034165, HMDB0034606, HMDB0036234, HMDB0037143, HMDB0037164, HMDB0037288, HMDB0038040, HMDB0038603, HMDB0039216, HMDB0039219, HMDB0040221, HMDB0040295, HMDB0040430, HMDB0059822, HMDB00798, HMDB00847, HMDB30331, HMDB31291, HMDB31480, HMDB31557, HMDB32042, HMDB32309, HMDB32406, HMDB32454, HMDB34162, HMDB34165, HMDB34606, HMDB36234, HMDB37143, HMDB37164, HMDB37288, HMDB38040, HMDB38603, HMDB39216, HMDB39219, HMDB40221, HMDB40295, HMDB40430, HMDB59822	Oenanthic ether	-0,135756941	0,218925646	0,469049541
158,0605072	HMDB0001190, HMDB0062503, HMDB01190, HMDB62503	Indoleacetaldehyde	-0,150058796	0,030957982	0,135509021
158,0812467	HMDB0000339, HMDB0000678, HMDB0000927, HMDB0011757, HMDB0012154, HMDB0012175, HMDB0029230, HMDB0029409, HMDB0029412, HMDB0031345, HMDB00339, HMDB0036384, HMDB0038593, HMDB0038625, HMDB0061678, HMDB00678, HMDB00927, HMDB11757, HMDB12154, HMDB12175, HMDB29230, HMDB29409, HMDB29412, HMDB31345, HMDB36384, HMDB38593, HMDB38625, HMDB61678	2-Methylbutyrylglycine	-0,045615604	0,425137366	0,664649654
158,9954464	HMDB0036807, HMDB0040199, HMDB36807, HMDB40199	(2-Furanylmethyl) methyl disulfide	0,025697484	0,907718462	0,961999186
159,0297876	HMDB0000225, HMDB0000398, HMDB00225, HMDB0039447, HMDB00398, HMDB0059737, HMDB39447, HMDB59737	Oxoadipate	0,02011578	0,855049701	0,93241486
159,045073	HMDB0032394, HMDB0036073, HMDB0041009, HMDB0060497, HMDB32394, HMDB36073, HMDB41009, HMDB60497	6-Methylcoumarin	-0,085206303	0,066610571	0,214186619
159,1023793	HMDB0000486, HMDB0000711, HMDB0001954, HMDB0002264, HMDB0010722, HMDB0013898, HMDB0013899, HMDB0013900, HMDB0031509, HMDB0032270, HMDB0032827, HMDB00486, HMDB0059825, HMDB0059867, HMDB0062596, HMDB00711, HMDB01954, HMDB02264, HMDB10722, HMDB13898, HMDB13899, HMDB13900, HMDB31509, HMDB32270, HMDB32827, HMDB59825, HMDB59867, HMDB62596	Propylhydroxypentanoic acid	0,026298056	0,804089587	0,904082001
159,1131942	HMDB0002038, HMDB0006009, HMDB02038, HMDB06009	Methyl-Lys	-0,325329917	0,895355317	0,95680127
160,040349	HMDB0002285, HMDB0003320, HMDB0004077, HMDB0031172, HMDB0060289, HMDB02285, HMDB03320, HMDB04077, HMDB31172, HMDB60289	2-Indolecarboxylic acid	-0,07365655	0,743137046	0,87098858
160,0608929	HMDB0000510, HMDB0029423, HMDB0033747, HMDB00510, HMDB0061715, HMDB0062557, HMDB0062660, HMDB0094716, HMDB29423, HMDB33747, HMDB61715, HMDB62557, HMDB62660, HMDB94716	Aminoadipate	0,127889407	0,095364226	0,271586644
161,0449024	HMDB0000321, HMDB0000345, HMDB0000355, HMDB0000368, HMDB0000640, HMDB0029934, HMDB00321, HMDB0032873, HMDB00345, HMDB00355, HMDB00368, HMDB0041561, HMDB0059758, HMDB00640, HMDB29934, HMDB32873, HMDB41561, HMDB59758	Hydroxyadipate	0,002783773	0,095130264	0,271586644
161,9872469	HMDB0033585, HMDB33585	Acesulfame	-0,443045846	0,283025412	0,546386287
162,0936515	HMDB0039661, HMDB0040015, HMDB0040022, HMDB0040023, HMDB0040024, HMDB0040025, HMDB0040026, HMDB0041927, HMDB0061786, HMDB39661, HMDB40015, HMDB40022, HMDB40023, HMDB40024, HMDB40025, HMDB40026, HMDB41927, HMDB61786	(R)-2-Methylimino-1-phenylpropan-1-ol	0,300719246	0,285659016	0,547460795
163,0606688	HMDB0000174, HMDB0000849, HMDB0002712, HMDB0003081, HMDB0005876, HMDB0010207, HMDB0012327, HMDB00174, HMDB0029196, HMDB0033821, HMDB0059624, HMDB0059625, HMDB0060267, HMDB0062477, HMDB0062489, HMDB00849, HMDB0124735, HMDB02712, HMDB03081, HMDB05876, HMDB10207, HMDB11726, HMDB11736, HMDB12327, HMDB29196, HMDB33821, HMDB59624, HMDB59625, HMDB60267, HMDB62477, HMDB62489	Deoxyhexose	0,199994844	0,450667835	0,684638244
163,1123299	HMDB0029653, HMDB0031311, HMDB0031454, HMDB0031624, HMDB0031625, HMDB0031866, HMDB0032060, HMDB0035601, HMDB0035989, HMDB0036719, HMDB0036814, HMDB0036816, HMDB0037294, HMDB29653, HMDB31311, HMDB31454, HMDB31624, HMDB31625, HMDB31866, HMDB32060, HMDB35601, HMDB35989, HMDB36719, HMDB36814, HMDB36816, HMDB37294	1-(1-Methoxy-1-methylethyl)-4-methylbenzene	-0,138098765	0,344969609	0,604043171
164,0711161	HMDB0000159, HMDB0001007, HMDB0004992, HMDB0006044, HMDB00159, HMDB0033485, HMDB0033589, HMDB0034169, HMDB0059934, HMDB00612, HMDB0062402, HMDB0062407, HMDB01007, HMDB04992, HMDB06044, HMDB33485, HMDB33589, HMDB34169, HMDB59934, HMDB62402, HMDB62407	Phenylalanine	-0,180543713	0,04089127	0,162818208
165,0195685	HMDB0002107, HMDB0002334, HMDB0002428, HMDB0032613, HMDB02107, HMDB02334, HMDB02428, HMDB32613	Phthalate	-0,042652168	0,993228403	0,995511687
165,0402935	HMDB0000539, HMDB0000867, HMDB00539, HMDB0059750, HMDB0060255, HMDB0060256, HMDB00867, HMDB59750, HMDB60255, HMDB60256	Pentonic acid	-0,128580608	0,303767199	0,563585101
165,0554557	HMDB0000375, HMDB0000563, HMDB0000748, HMDB0000779, HMDB0002072, HMDB0002199, HMDB0002229, HMDB0005175, HMDB0029665, HMDB0029817, HMDB0031132, HMDB0032030, HMDB0032138, HMDB0032573, HMDB0032605, HMDB0032639, HMDB0033752, HMDB0034243, HMDB0034993, HMDB00375, HMDB0040645, HMDB0041611, HMDB0041683, HMDB00475, HMDB00563, HMDB0059969, HMDB0062348, HMDB0062590, HMDB00748, HMDB00779, HMDB0124925, HMDB0125519, HMDB0125525, HMDB0125539, HMDB0134032, HMDB0134033, HMDB0134034, HMDB0134035, HMDB0134036, HMDB0134037, HMDB0134038, HMDB0137472, HMDB0141765, HMDB0141766, HMDB0142136, HMDB0142137, HMDB02072, HMDB02199, HMDB02229, HMDB05175, HMDB29665, HMDB29817, HMDB31132, HMDB32030, HMDB32138, HMDB32573, HMDB32605, HMDB32639, HMDB33752, HMDB34243, HMDB34993, HMDB40645, HMDB41611, HMDB41683, HMDB59969, HMDB61145, HMDB62348, HMDB62590	3-(3-Hydroxyphenyl)propano acid	0,144018715	0,394742839	0,641527233
165,0744256	HMDB0038183, HMDB38183	3-(4-Methyl-3-pentenyl)thiophene	-0,080806516	0,193827551	0,434492608

m/z	HMDB ID alternatives	Suggested annotation	Comparison [vegan] - [omnivore] Fold Change (log2)	p-value	p-value (BH adj.)
165,0916573	HMDB0004586, HMDB0029691, HMDB0031085, HMDB0032062, HMDB0032135, HMDB0032462, HMDB0032615, HMDB0034922, HMDB0035063, HMDB0035235, HMDB0035328, HMDB0036042, HMDB0036084, HMDB0036878, HMDB0036879, HMDB0037011, HMDB0037012, HMDB0037044, HMDB0039382, HMDB0040176, HMDB0041000, HMDB0041584, HMDB0062603, HMDB0127496, HMDB0135742, HMDB04586, HMDB06539, HMDB29691, HMDB31085, HMDB32062, HMDB32135, HMDB32462, HMDB32615, HMDB34922, HMDB35063, HMDB35235, HMDB35328, HMDB36042, HMDB36084, HMDB36878, HMDB36879, HMDB37011, HMDB37012, HMDB37044, HMDB39382, HMDB40176, HMDB41000, HMDB41584, HMDB62603	C10:3	1,321494117	0,002584963	0,02284884
165,9785589	HMDB0030524, HMDB30524	2(3H)-Benzothiazolethione	-0,708087686	0,094916778	0,271586644
166,0644888	HMDB0029873, HMDB0060362, HMDB29873, HMDB60362	(S)-Spinacine	-0,100797723	0,034791216	0,146086702
167,0212665	HMDB0000289, HMDB00289	Urate	0,236160953	0,177254038	0,413276794
167,1068623	HMDB0013105, HMDB0031156, HMDB0031273, HMDB0032219, HMDB0032312, HMDB0032313, HMDB0032319, HMDB0032434, HMDB0033700, HMDB0034452, HMDB0034670, HMDB0035714, HMDB0035766, HMDB0035829, HMDB0036067, HMDB0036103, HMDB0037014, HMDB0037175, HMDB0037821, HMDB0040639, HMDB13105, HMDB31156, HMDB31273, HMDB32219, HMDB32312, HMDB32313, HMDB32319, HMDB32434, HMDB33700, HMDB34452, HMDB34670, HMDB35714, HMDB35766, HMDB35829, HMDB36067, HMDB36103, HMDB37014, HMDB37175, HMDB37821, HMDB40639	C10:2	0,482848338	0,054801105	0,191146254
168,0772616	HMDB0000001, HMDB00001, HMDB0000479, HMDB00479, HMDB04935, HMDB06703, HMDB06704	Methyl-His	-1,335979666	0,000101561	0,001689068
169,0646463	HMDB0032412, HMDB0032582, HMDB0034446, HMDB32412, HMDB32582, HMDB34446	Methyl beta-naphthyl ketone	0,028617066	4,46484E-05	0,000889646
169,0854384	HMDB0030471, HMDB0038276, HMDB30471, HMDB38276	1,4-Ipomeadiol	-0,111860751	0,025424416	0,116156048
169,1229977	HMDB0004980, HMDB0010726, HMDB0012183, HMDB0029358, HMDB0029859, HMDB0030031, HMDB0031002, HMDB0031003, HMDB0031297, HMDB0031362, HMDB0031440, HMDB0031441, HMDB0032119, HMDB0032275, HMDB0032393, HMDB0032452, HMDB0033203, HMDB0033377, HMDB0034427, HMDB0034581, HMDB0034880, HMDB0035122, HMDB0035365, HMDB0035837, HMDB0035839, HMDB0035907, HMDB0036099, HMDB0036211, HMDB0036990, HMDB0036992, HMDB0037021, HMDB0037116, HMDB0037217, HMDB0037497, HMDB0037632, HMDB0038744, HMDB0039149, HMDB0039768, HMDB0039795, HMDB0040194, HMDB0040208, HMDB0040213, HMDB0040330, HMDB0040530, HMDB0040726, HMDB0040727, HMDB0041012, HMDB0041630, HMDB0059610, HMDB0059852, HMDB04980, HMDB10726, HMDB12183, HMDB29358, HMDB29859, HMDB30031, HMDB31002, HMDB31003, HMDB31297, HMDB31362, HMDB31440, HMDB31441, HMDB32119, HMDB32275, HMDB32393, HMDB32452, HMDB33203, HMDB33377, HMDB34427, HMDB34581, HMDB34880, HMDB35122, HMDB35365, HMDB35837, HMDB35839, HMDB35907, HMDB36099, HMDB36211, HMDB36990, HMDB36992, HMDB37021, HMDB37116, HMDB37217, HMDB37497, HMDB37632, HMDB38744, HMDB39149, HMDB39768, HMDB39795, HMDB40194, HMDB40208, HMDB40213, HMDB40330, HMDB40530, HMDB40726, HMDB40727, HMDB41012, HMDB41630, HMDB59610, HMDB59852	C10:1	-0,087011157	0,606239377	0,776271273
170,0450396	HMDB0012289, HMDB12289	Tetrahydrodipicolinate	0,125926492	0,008029133	0,052613508
170,0591349	HMDB0015052, HMDB15052	Metronidazole	0,160308353	0,208137149	0,453738986
171,0440012	HMDB0001892, HMDB0033765, HMDB01892, HMDB33765	Menadione	-0,270371629	0,004525633	0,034020276
171,0773454	HMDB0000721, HMDB0011178, HMDB00721, HMDB11178	Glycylproline	-0,044904592	0,027877542	0,124662648
171,1025185	HMDB0036188, HMDB0036213, HMDB0036396, HMDB0040165, HMDB0040202, HMDB0040203, HMDB0059936, HMDB0059937, HMDB0060287, HMDB0094711, HMDB36188, HMDB36213, HMDB36396, HMDB40165, HMDB40202, HMDB40203, HMDB59936, HMDB59937, HMDB60287, HMDB94711	Tetrahydrofurfuryl butyrate	0,043967085	0,220658923	0,470451297
171,1387627	HMDB0000511, HMDB0029760, HMDB0030029, HMDB0031264, HMDB0031739, HMDB0032045, HMDB0032070, HMDB0032155, HMDB0032264, HMDB0032302, HMDB0032315, HMDB0033620, HMDB0034457, HMDB0034849, HMDB0035595, HMDB0035723, HMDB0036136, HMDB0036145, HMDB0036228, HMDB0037387, HMDB0037849, HMDB0038038, HMDB0038602, HMDB0038783, HMDB0039218, HMDB0040157, HMDB0040167, HMDB0040195, HMDB0040211, HMDB0040529, HMDB0041216, HMDB00511, HMDB0061805, HMDB0094654, HMDB29760, HMDB30029, HMDB31264, HMDB31739, HMDB32045, HMDB32070, HMDB32155, HMDB32264, HMDB32302, HMDB32315, HMDB33620, HMDB34457, HMDB34849, HMDB35595, HMDB35723, HMDB36136, HMDB36145, HMDB36228, HMDB37387, HMDB37849, HMDB38038, HMDB38602, HMDB38783, HMDB39218, HMDB40157, HMDB40167, HMDB40195, HMDB40211, HMDB40529, HMDB41216, HMDB61805, HMDB94654	Decanoic acid (FA10:0)	-0,849861907	0,000429759	0,006044346
172,0392192	HMDB0000842, HMDB0062188, HMDB00842, HMDB62188	2-Quinolinecarboxylic acid	0,050427819	0,43569191	0,675744189
172,0956431	HMDB0000701, HMDB0000747, HMDB0002087, HMDB0011756, HMDB0061684, HMDB00701, HMDB00747, HMDB02087, HMDB11756, HMDB61684	Acetyl-Leu	-0,175970606	0,000787676	0,009539634
172,9913014	HMDB0060015, HMDB60015	Phenol sulfate	0,661931191	0,105862292	0,294788907
173,0706256	HMDB0006240, HMDB0029739, HMDB06240, HMDB29739	Indole-acetamide	0,302859366	0,036765601	0,149114436
173,0810521	HMDB0000893, HMDB0002023, HMDB0033838, HMDB0041606, HMDB0059727, HMDB0059757, HMDB0060684, HMDB00837, HMDB00893, HMDB02023, HMDB33838, HMDB41606, HMDB59727, HMDB59757, HMDB60684	Suberic acid	0,499222611	0,093449354	0,268936755
173,0932306	HMDB0003357, HMDB0028854, HMDB0029127, HMDB0034365, HMDB0062764, HMDB0094702, HMDB03357, HMDB06489, HMDB28854, HMDB29127, HMDB34365, HMDB62764, HMDB94702	Acetylornithine	1,314516877	0,103130849	0,28916431
173,1037355	HMDB0000517, HMDB0003416, HMDB00517, HMDB03416	Arginine	0,359311726	0,031080051	0,135509021
174,088235	HMDB0000904, HMDB0003148, HMDB00904, HMDB03148	Citrulline	0,001437825	0,501885316	0,722211629
175,0247241	HMDB0000044, HMDB00044, HMDB0006355, HMDB0031193, HMDB06355, HMDB14273, HMDB29945, HMDB31193	Hexuronic acid, Ascorbate	0,256942481	0,249244669	0,503707162

m/z	HMDB ID alternatives	Suggested annotation	Comparison [vegan] - [omnivore] Fold Change (log2)	p-value	p-value (BH adj.)
176,0691789	HMDB0012271, HMDB12271	O-Ureidohomoserine	-0,115243146	7,96159E-05	0,001416321
177,0547631	HMDB0002040, HMDB0030903, HMDB0031055, HMDB0033248, HMDB0038712, HMDB0038965, HMDB0039471, HMDB0062273, HMDB0062729, HMDB0125103, HMDB0125518, HMDB0131168, HMDB0133457, HMDB0133611, HMDB0133612, HMDB0133613, HMDB0133614, HMDB0133615, HMDB0134055, HMDB0135647, HMDB0135649, HMDB0135650, HMDB0135651, HMDB0141076, HMDB0141077, HMDB0141078, HMDB0141782, HMDB02040, HMDB30903, HMDB31055, HMDB33248, HMDB38712, HMDB38965, HMDB39471, HMDB62273, HMDB62729	4-Methoxycinnamic acid	0,064574967	7,23934E-07	3,32248E-05
177,0916459	HMDB0002043, HMDB0031250, HMDB0031864, HMDB0033376, HMDB0033386, HMDB0033597, HMDB0035013, HMDB0036235, HMDB0036385, HMDB0037680, HMDB0037708, HMDB0038179, HMDB0040287, HMDB0040356, HMDB0040411, HMDB0040425, HMDB0040426, HMDB0040583, HMDB0040800, HMDB0041553, HMDB0126149, HMDB0126449, HMDB0132981, HMDB0133181, HMDB0133469, HMDB0133682, HMDB0133683, HMDB0133684, HMDB0133685, HMDB0133686, HMDB0133687, HMDB02043, HMDB31250, HMDB31864, HMDB33376, HMDB33386, HMDB33597, HMDB35013, HMDB36235, HMDB36385, HMDB37680, HMDB37708, HMDB38179, HMDB40287, HMDB40356, HMDB40411, HMDB40425, HMDB40426, HMDB40583, HMDB40800, HMDB41553	5-Phenylvaleric acid	0,014786403	0,027716248	0,12458025
178,0511175	HMDB0000714, HMDB0000992, HMDB0012884, HMDB0032398, HMDB0032595, HMDB00714, HMDB00992, HMDB12884, HMDB32398, HMDB32595	Hippurate	-1,04246293	0,068806654	0,21505162
179,0556448	HMDB0000122, HMDB0000143, HMDB0000169, HMDB0000211, HMDB0000346, HMDB0000516, HMDB0000660, HMDB0001151, HMDB0001266, HMDB0003345, HMDB0003418, HMDB0003449, HMDB0006088, HMDB00122, HMDB0012326, HMDB00143, HMDB00160, HMDB00169, HMDB00211, HMDB0032222, HMDB0033704, HMDB0034220, HMDB00346, HMDB00516, HMDB0061922, HMDB0062138, HMDB0062170, HMDB0062202, HMDB0062473, HMDB0062538, HMDB00660, HMDB01151, HMDB0125599, HMDB01266, HMDB02256, HMDB0240207, HMDB0240208, HMDB0240209, HMDB0240210, HMDB0240211, HMDB03340, HMDB03345, HMDB03418, HMDB03449, HMDB05762, HMDB06088, HMDB06564, HMDB12326, HMDB32222, HMDB33704, HMDB34220, HMDB61922, HMDB62138, HMDB62170, HMDB62202, HMDB62473, HMDB62538	Hexose	-0,041074698	0,008653188	0,055077223
180,0657557	HMDB0000158, HMDB0001119, HMDB0002184, HMDB0003831, HMDB0006050, HMDB00158, HMDB0059720, HMDB0060280, HMDB00647, HMDB01119, HMDB02184, HMDB03831, HMDB06050, HMDB59720, HMDB60280	Tyrosine	0,019402295	0,801187799	0,902630182
181,0504741	HMDB0000118, HMDB0000333, HMDB0000423, HMDB0000755, HMDB0002643, HMDB0004061, HMDB00118, HMDB0029232, HMDB0029273, HMDB0029646, HMDB00333, HMDB0033624, HMDB0037274, HMDB0041270, HMDB00423, HMDB0059763, HMDB0062595, HMDB00755, HMDB0124923, HMDB0125533, HMDB0125591, HMDB0125595, HMDB0126386, HMDB0127495, HMDB0129348, HMDB0131428, HMDB0133494, HMDB0133788, HMDB0134042, HMDB0134043, HMDB0140294, HMDB0140892, HMDB02643, HMDB04061, HMDB04285, HMDB06776, HMDB29232, HMDB29273, HMDB29646, HMDB33624, HMDB37274, HMDB41270, HMDB59763, HMDB62595	Homovanillic acid	0,081088144	0,076135332	0,231324074
181,9913406	HMDB0029723, HMDB29723	Saccharin	0,077056981	0,848655255	0,92735261
182,0454679	HMDB0000017, HMDB00017	4-Pyridoxate	1,23886686	0,029295732	0,129674511
182,0707159	HMDB0033141, HMDB33141	2-Amino-a-carboline	0,386493321	0,062250916	0,204664919
183,0484016	HMDB0032401, HMDB32401	(+/-)-3-[(2-methyl-3-furyl)thio]-2-butanone	0,433431344	0,021799874	0,106794887
183,0672073	HMDB0001490, HMDB0035056, HMDB0036072, HMDB0039650, HMDB0040603, HMDB0059730, HMDB0125593, HMDB0125597, HMDB0126618, HMDB0126619, HMDB0126620, HMDB0131233, HMDB01490, HMDB02909, HMDB06775, HMDB35056, HMDB36072, HMDB39650, HMDB40603, HMDB59730	7-Methyl-2-hydroxy-6-oxoocta-2,4-dienoate	0,092968986	0,359556969	0,61597972
183,0672073	HMDB0001565, HMDB00284, HMDB01565	Choline-P	0,092968986	0,359556969	0,61597972
183,0672073	HMDB0029748, HMDB29748	2-Aminodipyrido[1,2-a:3”,2”-d]imidazole	0,092968986	0,359556969	0,61597972
183,1025652	HMDB0036117, HMDB0036998, HMDB0039052, HMDB0039710, HMDB0040883, HMDB36117, HMDB36998, HMDB39052, HMDB39710, HMDB40883	5-exo-Hydroxy-1,2-campholide	0,128917821	0,033715789	0,143415452
183,1381593	HMDB0031062, HMDB0031063, HMDB0031087, HMDB0031272, HMDB0031406, HMDB0031691, HMDB0031843, HMDB0032308, HMDB0032320, HMDB0032396, HMDB0032404, HMDB0032453, HMDB0032461, HMDB0032489, HMDB0032534, HMDB0033371, HMDB0033724, HMDB0034428, HMDB0034429, HMDB0036140, HMDB0036784, HMDB0037106, HMDB0037185, HMDB0037225, HMDB0037309, HMDB0038273, HMDB0038274, HMDB0038277, HMDB0038278, HMDB0038311, HMDB0038906, HMDB0040593, HMDB31062, HMDB31063, HMDB31087, HMDB31272, HMDB31406, HMDB31691, HMDB31843, HMDB32308, HMDB32320, HMDB32396, HMDB32404, HMDB32453, HMDB32461, HMDB32489, HMDB32534, HMDB33371, HMDB33724, HMDB34428, HMDB34429, HMDB36140, HMDB36784, HMDB37106, HMDB37185, HMDB37225, HMDB37309, HMDB38273, HMDB38274, HMDB38277, HMDB38278, HMDB38311, HMDB38906, HMDB40593	Citronellyl formate	-0,233857684	0,085318748	0,253054245
184,0973621	HMDB0006348, HMDB0006548, HMDB0094728, HMDB0094729, HMDB0094730, HMDB0094731, HMDB06348, HMDB06548, HMDB94728, HMDB94729, HMDB94730, HMDB94731	Pseudoecgonine	-0,556208366	0,022432472	0,109279974
184,9878352	HMDB0061736, HMDB61736	Diethyldithiophosphate	-0,020195668	0,989974372	0,99357422
185,0566729	HMDB0061890, HMDB61890	Pyroglutamylglycine	-0,112861735	0,600317019	0,772089145
185,1178601	HMDB0010724, HMDB0030367, HMDB0030369, HMDB0033010, HMDB0038692, HMDB0038978, HMDB0039533, HMDB0039582, HMDB0039587, HMDB0040204, HMDB0040205, HMDB0041376, HMDB0041545, HMDB10724, HMDB30367, HMDB30369, HMDB33010, HMDB38692, HMDB38978, HMDB39533, HMDB39582, HMDB39587, HMDB40204, HMDB40205, HMDB41376, HMDB41545	3-Oxodecanoic acid	0,561914126	0,068206141	0,214705473

m/z	HMDB ID alternatives	Suggested annotation	Comparison [vegan] - [omnivore] Fold Change (log2)	p-value	p-value (BH adj.)
185,1541854	HMDB0000947, HMDB0002373, HMDB0032044, HMDB0032325, HMDB0032448, HMDB0033618, HMDB0033848, HMDB0034134, HMDB0034456, HMDB0034458, HMDB0034460, HMDB0036034, HMDB0036229, HMDB0038237, HMDB0038267, HMDB0039215, HMDB0040171, HMDB0040193, HMDB0040266, HMDB0041559, HMDB0059831, HMDB0059860, HMDB0062119, HMDB0094659, HMDB00947, HMDB02373, HMDB32044, HMDB32325, HMDB32448, HMDB33618, HMDB33848, HMDB34134, HMDB34456, HMDB34458, HMDB34460, HMDB36034, HMDB36229, HMDB38237, HMDB38267, HMDB39215, HMDB40171, HMDB40193, HMDB40266, HMDB41559, HMDB59831, HMDB59860, HMDB62119, HMDB94659	Undecanoic acid	-0,228969409	0,001643841	0,017064634
186,055608	HMDB0000734, HMDB00734	Indoleacrylic acid	0,896632568	0,003372353	0,027228624
187,0073655	HMDB0011635, HMDB0034782, HMDB11635, HMDB34782	p-Cresol sulfate	-0,675351815	0,22540592	0,473219052
187,1326573	HMDB0002203, HMDB0010725, HMDB0033201, HMDB0033574, HMDB0033640, HMDB0034783, HMDB0038186, HMDB0039470, HMDB0039668, HMDB0039669, HMDB0039893, HMDB0040329, HMDB0041600, HMDB02203, HMDB10725, HMDB33201, HMDB33574, HMDB33640, HMDB34783, HMDB38186, HMDB39470, HMDB39668, HMDB39669, HMDB39893, HMDB40329, HMDB41600	3-Hydroxycapric acid	0,118272928	0,376054265	0,628197929
188,0713057	HMDB0002302, HMDB0014970, HMDB0029738, HMDB02302, HMDB14970, HMDB29738	3-Indolepropionic acid	0,418964868	0,403024195	0,646738154
188,9867414	HMDB0059724, HMDB0061713, HMDB59724, HMDB61713	Pyrocatechol sulfate	0,195856679	0,733591813	0,867529672
189,0394304	HMDB0012710, HMDB0059927, HMDB12710, HMDB59927	3-Dehydroquinate	0,044842714	0,96573237	0,984003393
189,0873166	HMDB0001370, HMDB0028697, HMDB0029054, HMDB0031412, HMDB0039222, HMDB01370, HMDB28697, HMDB29054, HMDB31412, HMDB39222	Diaminopimelate	0,13355622	0,65355842	0,812985652
189,0947498	HMDB0032346, HMDB0032372, HMDB0032427, HMDB32346, HMDB32372, HMDB32427	(+/-)-Isobutyl 3-methylthiobutyrate	0,205700288	0,995221078	0,996363697
189,0982124	HMDB0004224, HMDB0034326, HMDB04224, HMDB34326	Hydroxy-Arg	0,317279429	0,396582348	0,642625361
190,9747718	HMDB0060347, HMDB60347	2-Chloromaleylacetate	0,062465649	0,356943729	0,614412217
191,0192537	HMDB0000094, HMDB0000193, HMDB0001874, HMDB0005971, HMDB0006511, HMDB00094, HMDB00193, HMDB0033717, HMDB0041862, HMDB01874, HMDB05971, HMDB06511, HMDB33717, HMDB41862	(Iso)Citrate	-0,106838925	0,310115187	0,569306197
191,0329619	HMDB0031186, HMDB31186	N-Nitrosothialdine	-0,117768505	0,266455962	0,528067269
191,1432326	HMDB0029820, HMDB0029821, HMDB0029822, HMDB0029823, HMDB0029824, HMDB0029825, HMDB0031733, HMDB0032498, HMDB0032541, HMDB0033545, HMDB0034671, HMDB0034959, HMDB0034979, HMDB0035682, HMDB0035753, HMDB0036022, HMDB0036027, HMDB0036144, HMDB0036565, HMDB0036684, HMDB0036818, HMDB0037139, HMDB0041615, HMDB0059883, HMDB29820, HMDB29821, HMDB29822, HMDB29823, HMDB29824, HMDB29825, HMDB31733, HMDB32498, HMDB32541, HMDB33545, HMDB34671, HMDB34959, HMDB34979, HMDB35682, HMDB35753, HMDB36022, HMDB36027, HMDB36144, HMDB36565, HMDB36684, HMDB36818, HMDB37139, HMDB41615, HMDB59883	2,6-Diisopropyl-3-methylphenol	-0,01379523	0,717426283	0,860243607
192,0662122	HMDB0000821, HMDB0000859, HMDB0011723, HMDB0013245, HMDB0013292, HMDB0032388, HMDB0034250, HMDB0060366, HMDB0060389, HMDB0060758, HMDB00821, HMDB00859, HMDB11723, HMDB13245, HMDB13292, HMDB32388, HMDB34250, HMDB60366, HMDB60389, HMDB60758	Phenylacetylglycine	0,00545956	0,722226088	0,862713902
193,0717558	HMDB0029915, HMDB0029916, HMDB0029965, HMDB0031437, HMDB0031449, HMDB0033816, HMDB0034219, HMDB0034221, HMDB0034222, HMDB0062564, HMDB29915, HMDB29916, HMDB29965, HMDB31437, HMDB31449, HMDB33816, HMDB34219, HMDB34221, HMDB34222, HMDB62564	D-4-O-Methyl-myo-inositol	0,200013416	0,401970702	0,646713011
193,0854635	HMDB0031517, HMDB0031984, HMDB0032575, HMDB0032590, HMDB0034206, HMDB0034990, HMDB0036432, HMDB0037271, HMDB0040730, HMDB0041194, HMDB0041505, HMDB0041506, HMDB0041666, HMDB0059898, HMDB0059940, HMDB0059941, HMDB0059942, HMDB0059943, HMDB0059944, HMDB0059945, HMDB0124918, HMDB0126161, HMDB0126162, HMDB0126163, HMDB0126262, HMDB0126462, HMDB0126463, HMDB0126464, HMDB0126465, HMDB0126466, HMDB0132270, HMDB0133190, HMDB0133191, HMDB0133192, HMDB0133193, HMDB0133194, HMDB0133463, HMDB0133484, HMDB0133515, HMDB0133538, HMDB0141323, HMDB31517, HMDB31984, HMDB32575, HMDB32590, HMDB34206, HMDB34990, HMDB36432, HMDB37271, HMDB40730, HMDB41194, HMDB41505, HMDB41506, HMDB41666, HMDB59898, HMDB59940, HMDB59941, HMDB59942, HMDB59943, HMDB59944, HMDB59945	(R)-3-Hydroxy-5-phenylpentanoic acid	0,044930383	0,585322921	0,762941837
193,1225955	HMDB0014018, HMDB0031840, HMDB0032567, HMDB0034450, HMDB0035126, HMDB0035375, HMDB0035376, HMDB0035801, HMDB0037018, HMDB0037231, HMDB0037389, HMDB0038046, HMDB0038292, HMDB0039685, HMDB0040439, HMDB0041497, HMDB0041610, HMDB0132986, HMDB14018, HMDB31840, HMDB32567, HMDB34450, HMDB35126, HMDB35375, HMDB35376, HMDB35801, HMDB37018, HMDB37231, HMDB37389, HMDB38046, HMDB38292, HMDB39685, HMDB40439, HMDB41497, HMDB41610	4-Hydroxypropofol	0,626081249	0,084895788	0,253054245
194,0450549	HMDB0000840, HMDB0001229, HMDB0002016, HMDB0002404, HMDB0004067, HMDB0006116, HMDB0013678, HMDB0030726, HMDB0038055, HMDB0039119, HMDB0060602, HMDB00840, HMDB01229, HMDB02016, HMDB02404, HMDB04067, HMDB06116, HMDB06340, HMDB13678, HMDB30726, HMDB38055, HMDB39119, HMDB60602	Salicyluric acid	0,080481954	0,937647058	0,975690018
194,0808955	HMDB0014903, HMDB0029217, HMDB0041943, HMDB0060065, HMDB0060351, HMDB14903, HMDB29217, HMDB41943, HMDB60065, HMDB60351	Methyl-Tyr	0,078144819	0,586944886	0,763904389
195,0501565	HMDB0000565, HMDB0000625, HMDB0003290, HMDB00565, HMDB00625, HMDB03290, HMDB03373	Hexonic acid	-0,008541179	0,82677107	0,917884016
195,0847441	HMDB0040241, HMDB40241	1-(2-Thienyl)-1-heptanone	0,251018976	0,079619313	0,239407038
195,1016176	HMDB0036226, HMDB0037725, HMDB0037734, HMDB0135672, HMDB36226, HMDB37725, HMDB37734	2,6-Dimethoxy-4-propylphenol	-1,61126169	0,002112907	0,019693506

m/z	HMDB ID alternatives	Suggested annotation	Comparison [vegan] - [omnivore] Fold Change (log2)	p-value	p-value (BH adj.)
195,111952	HMDB0029741, HMDB0040551, HMDB29741, HMDB40551	2-Isopropyl-3,5-dimethoxy-6-methylpyrazine	-1,333184847	0,001215469	0,013588325
195,13813	HMDB0030867, HMDB0030945, HMDB0031030, HMDB0031692, HMDB0032051, HMDB0032193, HMDB0032331, HMDB0032901, HMDB0034903, HMDB0035157, HMDB0036048, HMDB0036599, HMDB0037301, HMDB0038270, HMDB0038280, HMDB0039522, HMDB0040206, HMDB0040227, HMDB0040397, HMDB0040725, HMDB0041469, HMDB0041494, HMDB0061816, HMDB30867, HMDB30945, HMDB31030, HMDB31692, HMDB32051, HMDB32193, HMDB32331, HMDB32901, HMDB34903, HMDB35157, HMDB36048, HMDB36599, HMDB37301, HMDB38270, HMDB38280, HMDB39522, HMDB40206, HMDB40227, HMDB40397, HMDB40725, HMDB41469, HMDB41494, HMDB61816	Dihydro-5-(2-octenyl)-2(3H)-furanone	0,212086066	0,041589745	0,164049886
197,0420496	HMDB0040164, HMDB40164	4-Methyldibenzothiophene	0,037780431	0,824695788	0,917884016
197,0676826	HMDB0004400, HMDB0059771, HMDB04400, HMDB59771	5-Acetylamino-6-amino-3-methyluracil	-0,682206223	0,152735466	0,380720112
197,0803431	HMDB0004998, HMDB0030991, HMDB0033093, HMDB0034099, HMDB0059768, HMDB0059779, HMDB0131265, HMDB0135755, HMDB04998, HMDB30991, HMDB33093, HMDB34099, HMDB59768, HMDB59779	Guaifenesin	-0,105591813	0,175296952	0,412289381
197,0926746	HMDB0014606, HMDB14606	Metharbital	-0,071538386	0,240470495	0,494552526
197,1539661	HMDB0000529, HMDB0010729, HMDB0029585, HMDB0029763, HMDB0031683, HMDB0032214, HMDB0032235, HMDB0032248, HMDB0033378, HMDB0034160, HMDB0034844, HMDB0036182, HMDB0037186, HMDB0037305, HMDB0037329, HMDB0037498, HMDB0037742, HMDB0037813, HMDB0038081, HMDB0038269, HMDB0038895, HMDB0038924, HMDB0039220, HMDB0040158, HMDB0040163, HMDB0040361, HMDB0041264, HMDB0041449, HMDB00529, HMDB10729, HMDB29585, HMDB29763, HMDB31683, HMDB32214, HMDB32235, HMDB32248, HMDB33378, HMDB34160, HMDB34844, HMDB36182, HMDB37186, HMDB37305, HMDB37329, HMDB37498, HMDB37742, HMDB37813, HMDB38081, HMDB38269, HMDB38895, HMDB38924, HMDB39220, HMDB40158, HMDB40163, HMDB40361, HMDB41264, HMDB41449	C12:1	-0,29449552	0,06844968	0,214705473
198,088931	HMDB0060477, HMDB60477	gamma-Glutamyl-beta-aminopropiononitrile	-0,098804552	0,034454739	0,145847243
199,0371326	HMDB0037192, HMDB37192	Potassium 2-(1”-ethoxy) ethoxypropanoate	0,277197979	0,17541211	0,412289381
199,0974467	HMDB0000603, HMDB0013227, HMDB0030989, HMDB0030990, HMDB0034491, HMDB0034971, HMDB0036715, HMDB00603, HMDB13227, HMDB30989, HMDB30990, HMDB34491, HMDB34971, HMDB36715	cis-4-Decenedioic acid	-0,094355783	0,515353538	0,730874126
199,1697699	HMDB0000638, HMDB0030998, HMDB0032310, HMDB0032440, HMDB0033619, HMDB0034128, HMDB0034136, HMDB0034137, HMDB0035410, HMDB0036225, HMDB0040166, HMDB0059868, HMDB00638, HMDB30998, HMDB32310, HMDB32440, HMDB33619, HMDB34128, HMDB34136, HMDB34137, HMDB35410, HMDB36225, HMDB40166, HMDB59868	Dodecanoic acid (FA12:0)	0,110881254	0,67828546	0,834224148
199,9678561	HMDB0000731, HMDB00731	Sulfo-Cys	0,59816557	0,708848572	0,853751319
200,129109	HMDB0000832, HMDB0013116, HMDB0041540, HMDB0059745, HMDB00832, HMDB13116, HMDB41540, HMDB59745	Capryloylglycine	-0,011939013	0,671389624	0,828078858
201,0222307	HMDB0062551, HMDB0062721, HMDB62551, HMDB62721	4-ethylphenylsulfate	0,238235001	0,838341665	0,921212686
201,0759976	HMDB0032306, HMDB0033092, HMDB32306, HMDB33092	4-Heptenal diethyl acetal	-0,261045778	0,281335554	0,546386287
201,1238229	HMDB0028690, HMDB0028691, HMDB0028900, HMDB0028922, HMDB0094713, HMDB28690, HMDB28691, HMDB28900, HMDB28922, HMDB94713	Alanyl-Isoleucine	-0,143481144	0,531886657	0,745667467
201,1485984	HMDB0032443, HMDB0061654, HMDB32443, HMDB61654	3-Hydroxynonyl acetate	-0,453864252	6,25591E-08	4,54596E-06
202,0821324	HMDB0028682, HMDB0028724, HMDB0028797, HMDB0028839, HMDB0028855, HMDB28682, HMDB28724, HMDB28797, HMDB28839, HMDB28855	Alanyl-Asparagine	0,023501935	0,985964339	0,99357422
203,0021106	HMDB0060013, HMDB60013	O-methoxycatechol-O-sulphate	-0,544522618	0,057388101	0,196244799
203,0559769	HMDB0041031, HMDB41031	3,5,6-Trihydroxy-5-(hydroxymethyl)-2-methoxy-2-cyclohexen-1-one	-0,125073628	0,327383166	0,59105201
203,0818546	HMDB0000929, HMDB0013609, HMDB0013840, HMDB0014892, HMDB0030396, HMDB0060533, HMDB0060878, HMDB0060967, HMDB00929, HMDB13609, HMDB13840, HMDB14892, HMDB30396, HMDB60533, HMDB60878, HMDB60967	Tryptophan	-0,095465168	0,135995628	0,351893733
204,0661099	HMDB0000671, HMDB0004096, HMDB0011621, HMDB0030179, HMDB0032755, HMDB0059765, HMDB00671, HMDB04096, HMDB11621, HMDB30179, HMDB32755, HMDB59765	Indolelactic acid	-0,020071492	0,643588276	0,804024321
204,9810847	HMDB0059752, HMDB0060016, HMDB0060018, HMDB59752, HMDB60016, HMDB60018	5-Sulfoxymethylfurfural	-0,175489433	0,63800237	0,801639866
205,0148197	HMDB0128623, HMDB0128624, HMDB0128625, HMDB0131684, HMDB0136642, HMDB0136643	7-hydroxy-1-oxo-1H-isochromene-3-carboxylic acid	0,080218537	0,723668453	0,863254296
205,0490557	HMDB0029466, HMDB0029472, HMDB0029775, HMDB0030818, HMDB0032952, HMDB0035303, HMDB0036627, HMDB0129368, HMDB0136784, HMDB29466, HMDB29472, HMDB29775, HMDB30818, HMDB32952, HMDB35303, HMDB36627	Eugenitol	0,084276723	0,389080519	0,64014757

m/z	HMDB ID alternatives	Suggested annotation	Comparison [vegan] - [omnivore] Fold Change (log2)	p-value	p-value (BH adj.)
205,0646378	HMDB0028847, HMDB0028973, HMDB28847, HMDB28973	Glycyl-Methionine	0,03615653	0,725789765	0,864557648
205,0849516	HMDB0029049, HMDB0029070, HMDB29049, HMDB29070	Serinyl-Threonine	-0,049139641	0,293426463	0,554284447
205,1237757	HMDB0001925, HMDB0029669, HMDB0032176, HMDB0033196, HMDB0034469, HMDB0034470, HMDB0034472, HMDB0034556, HMDB0035008, HMDB0035016, HMDB0035017, HMDB0035181, HMDB0036025, HMDB0036240, HMDB0036386, HMDB0036391, HMDB0037194, HMDB0037604, HMDB0037623, HMDB0037720, HMDB0038605, HMDB0039101, HMDB0040431, HMDB0132932, HMDB0132940, HMDB01872, HMDB01925, HMDB29669, HMDB32176, HMDB33196, HMDB34469, HMDB34470, HMDB34472, HMDB34556, HMDB35008, HMDB35016, HMDB35017, HMDB35181, HMDB36025, HMDB36240, HMDB36386, HMDB36391, HMDB37194, HMDB37604, HMDB37623, HMDB37720, HMDB38605, HMDB39101, HMDB40431	Ibuprofen	0,155398494	0,575297943	0,762286527
205,1588799	HMDB0013825, HMDB0031737, HMDB0031738, HMDB0032397, HMDB0032528, HMDB0035245, HMDB0035631, HMDB0036023, HMDB0036024, HMDB0038130, HMDB0061831, HMDB0133179, HMDB13825, HMDB31737, HMDB31738, HMDB32397, HMDB32528, HMDB35245, HMDB35631, HMDB36023, HMDB36024, HMDB38130, HMDB61831	4-(1,1,3,3-Tetramethylbutyl)-phenol	0,029350653	0,012310888	0,070559298
207,1387491	HMDB0029668, HMDB0031869, HMDB0034672, HMDB0034984, HMDB0035228, HMDB0036686, HMDB0036821, HMDB0036824, HMDB0037776, HMDB0038047, HMDB0038529, HMDB0040233, HMDB0041636, HMDB0132947, HMDB29668, HMDB31869, HMDB34672, HMDB34984, HMDB35228, HMDB36686, HMDB36821, HMDB36824, HMDB37776, HMDB38047, HMDB38529, HMDB40233, HMDB41636	Eremopetasinorol	0,096502073	0,066810505	0,214186619
209,0314221	HMDB0000639, HMDB0000663, HMDB0029881, HMDB00639, HMDB00663, HMDB03435, HMDB29881	Galactaric acid	0,211494018	0,153685183	0,380720112
209,0451498	HMDB0011714, HMDB0035484, HMDB0125514, HMDB0125515, HMDB0125516, HMDB0127490, HMDB0127946, HMDB0127947, HMDB0132221, HMDB0132228, HMDB0140909, HMDB0141258, HMDB0141331, HMDB0142176, HMDB0142177, HMDB0142178, HMDB0142180, HMDB0142181, HMDB11714, HMDB35484	Vanilpyruvic acid	-0,20774287	0,574699852	0,762286527
209,0808315	HMDB0013070, HMDB0029187, HMDB0029233, HMDB0033798, HMDB0034047, HMDB0036199, HMDB0039428, HMDB0041406, HMDB0060737, HMDB0125803, HMDB0127493, HMDB0129253, HMDB0129254, HMDB0129255, HMDB0129256, HMDB0130385, HMDB0133478, HMDB0133511, HMDB0133519, HMDB0133526, HMDB0133535, HMDB0135671, HMDB13070, HMDB29187, HMDB29233, HMDB33798, HMDB34047, HMDB36199, HMDB39428, HMDB41406, HMDB60737	Sinapyl alcohol	-0,207032314	0,155134697	0,382139705
209,0929991	HMDB0015441, HMDB15441	Aprobarbital	0,021646827	0,633679962	0,801639866
209,1175289	HMDB0032797, HMDB0032871, HMDB0033102, HMDB0037104, HMDB0037135, HMDB0037642, HMDB0037735, HMDB0037816, HMDB0041573, HMDB32797, HMDB32871, HMDB33102, HMDB37104, HMDB37135, HMDB37642, HMDB37735, HMDB37816, HMDB41573	Jasmonic acid	0,083333593	0,494099682	0,718091538
209,1533475	HMDB0014631, HMDB14631	Altretamine	0,135461927	0,375326638	0,628197929
210,0876323	HMDB0015078, HMDB15078	Zalcitabine	-0,052840324	0,689517902	0,843281362
211,024341	HMDB0125210, HMDB0126568, HMDB0126571, HMDB0128683, HMDB0128685, HMDB0137303	2-oxo-3-(3,4,5-trihydroxyphenyl)propan acid	-0,421076937	0,007273369	0,048048317
211,0387964	HMDB0013696, HMDB0032997, HMDB0032999, HMDB13696, HMDB32997, HMDB32999	Urolithin B	-0,149967476	0,013399387	0,074083072
211,0757791	HMDB0014814, HMDB0032039, HMDB14814, HMDB32039	Benzyl Benzoate	-0,096625044	0,955822852	0,981716758
211,1089238	HMDB0011180, HMDB0014382, HMDB0015442, HMDB11180, HMDB14382, HMDB15442	L-prolyl-L-proline	-1,059075154	0,008730645	0,055167555
211,133311	HMDB0029388, HMDB0033601, HMDB0035085, HMDB0037326, HMDB0041572, HMDB29388, HMDB33601, HMDB35085, HMDB37326, HMDB41572	Cucurbic acid	0,127294227	0,04930686	0,178404903
211,1695585	HMDB0031017, HMDB0031028, HMDB0031741, HMDB0032792, HMDB0034286, HMDB0037187, HMDB0037226, HMDB0037626, HMDB0037907, HMDB0038078, HMDB0039805, HMDB0040451, HMDB31017, HMDB31028, HMDB31741, HMDB32792, HMDB34286, HMDB37187, HMDB37226, HMDB37626, HMDB37907, HMDB38078, HMDB39805, HMDB40451	10-Undecenyl acetate	-0,495287277	9,65089E-08	6,47352E-06
212,0020116	HMDB0000682, HMDB00682, HMDB03309	Indoxyl sulfate	-0,221030773	0,516305575	0,730874126
212,9822422	HMDB0006801, HMDB06801	2-Oxo-3-hydroxy-4-phosphobutanoic acid	-0,0651599	0,637247963	0,801639866
213,0223384	HMDB0135284	[(2E)-3-phenylprop-2-en-1-yl]oxysulfonic acid	0,581955269	0,320934184	0,583131918
213,1129206	HMDB0030984, HMDB0030985, HMDB0030986, HMDB30984, HMDB30985, HMDB30986	5-Hexyltetrahydro-2-oxo-3-furancarboxylic acid	-0,199649977	0,206883217	0,453271773
213,1239414	HMDB0003581, HMDB0029030, HMDB0029135, HMDB03581, HMDB29030, HMDB29135	Dethiobiotin	-0,27599797	0,018503286	0,095472577
213,1491265	HMDB0010727, HMDB0037398, HMDB10727, HMDB37398	3-Oxododecanoic acid	0,238326755	0,163599692	0,39192014
213,1850427	HMDB0000910, HMDB0029552, HMDB0031018, HMDB0032211, HMDB0034140, HMDB0036217, HMDB0036219, HMDB0037311, HMDB0038729, HMDB0059900, HMDB0061828, HMDB0061829, HMDB00910, HMDB29552, HMDB31018, HMDB32211, HMDB34140, HMDB36217, HMDB36219, HMDB37311, HMDB38729, HMDB59900, HMDB61828, HMDB61829	C13:0	-0,722417873	5,15127E-05	0,000976502
214,1442777	HMDB0013279, HMDB13279	N-Nonanoylglycine	-0,206725901	0,93944923	0,976400154
215,0333854	HMDB0014693, HMDB0030637, HMDB0138947, HMDB14693, HMDB30637	Methoxsalen	0,017370644	0,547242381	0,756072946
216,0972709	HMDB0000856, HMDB0028685, HMDB0028701, HMDB0028790, HMDB00856, HMDB28685, HMDB28701, HMDB28790	N-a-Acetylcitrulline	0,027446837	0,110949607	0,301395817

m/z	HMDB ID alternatives	Suggested annotation	Comparison [vegan] - [omnivore] Fold Change (log2)	p-value	p-value (BH adj.)
217,0861191	HMDB0040358, HMDB40358	(R)-Bitalin A	-0,098755183	0,013641534	0,074813947
218,0758672	HMDB0060827, HMDB0061056, HMDB60827, HMDB61056	ID11614	-0,02159889	0,443332228	0,679412483
218,102413	HMDB0000210, HMDB00210, HMDB0062717, HMDB62717	Pantothenic acid	0,132211147	0,287091714	0,548999945
219,1039003	HMDB0032589, HMDB0037492, HMDB0040424, HMDB0061823, HMDB0061942, HMDB0132933, HMDB0132935, HMDB0132936, HMDB0132937, HMDB0132939, HMDB0132948, HMDB32589, HMDB37492, HMDB40424, HMDB61823, HMDB61942	1-(5-Acetyl-2-hydroxyphenyl)-3-methyl-1-butanone	0,090133115	0,156306991	0,382286785
219,1394695	HMDB0013817, HMDB0036389, HMDB0037606, HMDB0037714, HMDB0037718, HMDB0038996, HMDB0039539, HMDB0040217, HMDB0040226, HMDB0126515, HMDB0126516, HMDB0126517, HMDB0126518, HMDB0126519, HMDB0126520, HMDB0133164, HMDB0133165, HMDB0133166, HMDB0133167, HMDB0133169, HMDB0133170, HMDB0133180, HMDB13817, HMDB36389, HMDB37606, HMDB37714, HMDB37718, HMDB38996, HMDB39539, HMDB40217, HMDB40226	2,6-Di-tert-butylbenzoquinone	0,138289454	0,460898794	0,692396623
219,1490211	HMDB0014888, HMDB14888	Prilocaine	-0,181233463	0,472145263	0,700188213
219,1732482	HMDB0013688, HMDB0030232, HMDB0032220, HMDB0033826, HMDB0034661, HMDB0034718, HMDB0034940, HMDB0035020, HMDB0035026, HMDB0035097, HMDB0035306, HMDB0035391, HMDB0035645, HMDB0035704, HMDB0035718, HMDB0035720, HMDB0035739, HMDB0035793, HMDB0036118, HMDB0036192, HMDB0036402, HMDB0036420, HMDB0036716, HMDB0036717, HMDB0036788, HMDB0036789, HMDB0036793, HMDB0036798, HMDB0037069, HMDB0037392, HMDB0037395, HMDB0037811, HMDB0038119, HMDB0038123, HMDB0038134, HMDB0038146, HMDB0038160, HMDB0038193, HMDB0038208, HMDB0038209, HMDB0038210, HMDB0038211, HMDB0038212, HMDB0038512, HMDB0038982, HMDB0039532, HMDB0039711, HMDB0040763, HMDB0059836, HMDB0060356, HMDB0134152, HMDB13688, HMDB30232, HMDB32220, HMDB33826, HMDB34661, HMDB34718, HMDB34940, HMDB35020, HMDB35026, HMDB35097, HMDB35306, HMDB35391, HMDB35645, HMDB35704, HMDB35718, HMDB35720, HMDB35739, HMDB35793, HMDB36118, HMDB36192, HMDB36402, HMDB36420, HMDB36716, HMDB36717, HMDB36788, HMDB36789, HMDB36793, HMDB36798, HMDB37069, HMDB37392, HMDB37395, HMDB37811, HMDB38119, HMDB38123, HMDB38134, HMDB38146, HMDB38160, HMDB38193, HMDB38208, HMDB38209, HMDB38210, HMDB38211, HMDB38212, HMDB38512, HMDB38982, HMDB39532, HMDB39711, HMDB40763, HMDB59836, HMDB60356	Nootkatol	0,178916043	0,446368831	0,680478358
221,0908307	HMDB0028848, HMDB0028995, HMDB28848, HMDB28995	Glycyl-Phenylalanine	-0,072965923	0,629485848	0,797895444
221,1542211	HMDB0035593, HMDB0036790, HMDB0036791, HMDB0037605, HMDB0037820, HMDB0040178, HMDB35593, HMDB36790, HMDB36791, HMDB37605, HMDB37820, HMDB40178	Rishitin	0,218808922	0,023318389	0,111723269
222,0524407	HMDB0031805, HMDB31805	(E)-Monocrotophos	-0,017644785	0,804550038	0,904082001
222,1128918	HMDB0015168, HMDB15168	Cerulenin	0,158609088	0,233330775	0,484439133
223,0598133	HMDB0030018, HMDB0032616, HMDB0034069, HMDB0041691, HMDB0127488, HMDB0128183, HMDB0128665, HMDB0128667, HMDB0128668, HMDB0128673, HMDB0128674, HMDB0140920, HMDB0141325, HMDB0142075, HMDB0142169, HMDB0142170, HMDB0142171, HMDB0142172, HMDB0142173, HMDB0142174, HMDB30018, HMDB32616, HMDB34069, HMDB41691	Elenolide	0,24166749	0,018161848	0,09426864
223,0975951	HMDB0030680, HMDB0030703, HMDB0032552, HMDB0032663, HMDB0036203, HMDB0040629, HMDB0040890, HMDB0124917, HMDB0124919, HMDB0124920, HMDB0125801, HMDB0127761, HMDB0127765, HMDB0135709, HMDB30680, HMDB30703, HMDB32552, HMDB32663, HMDB36203, HMDB40629, HMDB40890	Diplosporin	0,006522721	0,437063902	0,675744189
223,1332325	HMDB0029670, HMDB0032679, HMDB0032689, HMDB0034564, HMDB0036583, HMDB0037244, HMDB0037726, HMDB0037730, HMDB0037736, HMDB0112084, HMDB29670, HMDB32679, HMDB32689, HMDB34564, HMDB36583, HMDB37244, HMDB37726, HMDB37730, HMDB37736	Epoxyeremopetasinorol	-0,171760448	0,013423309	0,074083072
223,1694808	HMDB0000560, HMDB0030426, HMDB0030427, HMDB0031015, HMDB0032053, HMDB0032342, HMDB0032524, HMDB0036424, HMDB0037814, HMDB0038246, HMDB0038259, HMDB0038260, HMDB0039129, HMDB00560, HMDB30426, HMDB30427, HMDB31015, HMDB32053, HMDB32342, HMDB32524, HMDB36424, HMDB37814, HMDB38246, HMDB38259, HMDB38260, HMDB39129	5,8-Tetradecadienoic acid	0,339898313	0,160832318	0,389571615
225,0636575	HMDB0011105, HMDB11105	5-Acetylamino-6-formylamino-3-methyluracil	-0,000872935	0,63648574	0,801639866
225,1123883	HMDB0040705, HMDB0059729, HMDB40705, HMDB59729	Allixin	0,116711927	0,946004591	0,977865267
225,1847254	HMDB0000499, HMDB0000521, HMDB0002000, HMDB0005051, HMDB0010732, HMDB0030921, HMDB0031029, HMDB0031073, HMDB0032152, HMDB0032321, HMDB0034561, HMDB0037188, HMDB0037189, HMDB0037227, HMDB0037228, HMDB0037369, HMDB0037633, HMDB0038056, HMDB0040228, HMDB00499, HMDB00521, HMDB0062243, HMDB0062248, HMDB0094671, HMDB02000, HMDB05051, HMDB10732, HMDB30921, HMDB31029, HMDB31073, HMDB32152, HMDB32321, HMDB34561, HMDB37188, HMDB37189, HMDB37227, HMDB37228, HMDB37369, HMDB37633, HMDB38056, HMDB40228, HMDB62243, HMDB62248, HMDB94671	C14:1	-0,901546589	0,000219331	0,003355376
226,0344552	HMDB0006794, HMDB0125157, HMDB0126635, HMDB06794	5-(2”-Carboxyethyl)-4,6-Dihydroxypicolinate	-0,337750479	0,023273261	0,111723269
226,9645976	HMDB0037638, HMDB37638	Ammonium peroxydisulfate	0,005609415	0,350915406	0,609558236
227,0000132	HMDB0029735, HMDB29735	5-Ethynyl-5”-(1-propynyl)-2,2”-bithiophene	0,092604951	0,003313648	0,027004686
227,0361747	HMDB0013695, HMDB0030723, HMDB0030724, HMDB0142418, HMDB0142421, HMDB0142422, HMDB13695, HMDB30723, HMDB30724	Urolithin A	0,139648863	0,548192787	0,756072946

m/z	HMDB ID alternatives	Suggested annotation	Comparison [vegan] - [omnivore] Fold Change (log2)	p-value	p-value (BH adj.)
227,1030676	HMDB0006695, HMDB0028871, HMDB0029018, HMDB0040573, HMDB0094651, HMDB06695, HMDB28871, HMDB29018, HMDB40573, HMDB94651	Prolylhydroxyproline	0,033435033	0,499499473	0,722211629
227,1279562	HMDB0000933, HMDB0030994, HMDB00933, HMDB30994	Traumatic acid	1,333503349	0,106151052	0,294788907
227,2017582	HMDB0000806, HMDB0002221, HMDB0031072, HMDB0032318, HMDB0032548, HMDB0033788, HMDB0034461, HMDB0036216, HMDB0037312, HMDB0037495, HMDB0038034, HMDB0038969, HMDB0059865, HMDB0061781, HMDB00806, HMDB02221, HMDB31072, HMDB32318, HMDB32548, HMDB33788, HMDB34461, HMDB36216, HMDB37312, HMDB37495, HMDB38034, HMDB38969, HMDB59865, HMDB61781	C14:0	-0,743258701	0,000508989	0,006934977
228,1595911	HMDB0013267, HMDB13267	N-Decanoylglycine	-0,159239233	0,882026639	0,95071351
229,0170336	HMDB0127980, HMDB0134048, HMDB0135258, HMDB0135295, HMDB0135297, HMDB0135299, HMDB0135301, HMDB0135303, HMDB0135304, HMDB0135306, HMDB0135307, HMDB0135311, HMDB0135656, HMDB0135657	(4-ethenyl-2-methoxyphenyl)oxidanes acid	0,309408494	0,272491274	0,537009745
229,1441049	HMDB0000623, HMDB0030143, HMDB0041613, HMDB0041614, HMDB00623, HMDB30143, HMDB41613, HMDB41614	Dodecanedioic acid	0,436095836	0,1827255	0,417740595
230,0122787	HMDB0059911, HMDB0059994, HMDB59911, HMDB59994	Paracetamol sulfate	0,16650654	0,719171548	0,860243607
230,1136221	HMDB0028744, HMDB0029122, HMDB28744, HMDB29122	Asparaginyl-Valine	-0,025387218	0,943258499	0,977865267
231,0109505	HMDB0035145, HMDB35145	Chrycolide	0,024177367	0,827358758	0,917884016
231,0317723	HMDB0127988	(4-ethyl-2-methoxyphenyl)oxidanes acid	-0,181861041	0,036416504	0,149114436
231,0801554	HMDB0060324, HMDB60324	1-Methoxypyrene	0,129706132	0,738241076	0,867582504
231,093583	HMDB0032981, HMDB32981	2,3-Diphenylpyrazine	-0,036807378	0,986181584	0,99357422
232,0281433	HMDB0004148, HMDB0006275, HMDB04148, HMDB06275	Dopamine 4-sulfate	0,205840351	0,295840559	0,555441671
232,0428928	HMDB0038592, HMDB38592	Brassicanal B	0,127567217	0,320990046	0,583131918
232,0833197	HMDB0035923, HMDB35923	Hirsutin	0,087064836	0,955663727	0,981716758
232,978367	HMDB0014026, HMDB14026	p-Chlorobenzene sulfonyl urea	0,146287931	0,375779501	0,628197929
233,0103898	HMDB0034453, HMDB34453	4-[2,2”-Bithiophen-5-yl]-3-butyn-1-ol	0,031878051	0,06117565	0,203607508
233,0777455	HMDB0011169, HMDB0028763, HMDB0028828, HMDB0029057, HMDB0029158, HMDB11169, HMDB28763, HMDB28828, HMDB29057, HMDB29158	L-beta-aspartyl-L-threonine	-0,035882766	0,222310142	0,472815717
233,1536328	HMDB0030016, HMDB0031349, HMDB0032081, HMDB0033252, HMDB0033253, HMDB0033960, HMDB0034562, HMDB0034742, HMDB0034956, HMDB0035202, HMDB0035407, HMDB0035408, HMDB0035413, HMDB0035639, HMDB0035794, HMDB0035869, HMDB0035889, HMDB0035935, HMDB0036035, HMDB0036390, HMDB0036392, HMDB0036427, HMDB0036449, HMDB0036466, HMDB0036647, HMDB0037038, HMDB0037391, HMDB0037640, HMDB0037710, HMDB0037739, HMDB0038190, HMDB0038198, HMDB0038698, HMDB0038795, HMDB0038796, HMDB0038914, HMDB0039621, HMDB0041036, HMDB0134131, HMDB0134132, HMDB0134133, HMDB0134134, HMDB0134135, HMDB0134136, HMDB0134137, HMDB0134138, HMDB0134139, HMDB0134140, HMDB0134141, HMDB0134142, HMDB0134143, HMDB0134153, HMDB30016, HMDB31349, HMDB32081, HMDB33252, HMDB33253, HMDB33960, HMDB34562, HMDB34742, HMDB34956, HMDB35202, HMDB35407, HMDB35408, HMDB35413, HMDB35639, HMDB35794, HMDB35869, HMDB35889, HMDB35935, HMDB36035, HMDB36390, HMDB36392, HMDB36427, HMDB36449, HMDB36466, HMDB36647, HMDB37038, HMDB37391, HMDB37640, HMDB37710, HMDB37739, HMDB38190, HMDB38198, HMDB38698, HMDB38795, HMDB38796, HMDB38914, HMDB39621, HMDB41036	Valerenic acid	0,101630164	0,392265604	0,641527233
234,1122514	HMDB0033610, HMDB33610	Pandamarilactam 3x	0,511034006	0,225755878	0,473219052
235,0224504	HMDB0015021, HMDB15021	Isosorbide Dinitrate	-0,004241999	0,582476089	0,762836399
235,0302194	HMDB0033062, HMDB0036372, HMDB0036373, HMDB0038968, HMDB33062, HMDB36372, HMDB36373, HMDB38968	2-Propenyl 1-(2-propenylsulfinyl)propyl disulfide	0,039022029	0,665118366	0,822671228
235,1688421	HMDB0002352, HMDB0030891, HMDB0030916, HMDB0030920, HMDB0031348, HMDB0032048, HMDB0032292, HMDB0032681, HMDB0033083, HMDB0033104, HMDB0034714, HMDB0034722, HMDB0035032, HMDB0035040, HMDB0035127, HMDB0035147, HMDB0035301, HMDB0035648, HMDB0035827, HMDB0036446, HMDB0036549, HMDB0036668, HMDB0036680, HMDB0036687, HMDB0037393, HMDB0037906, HMDB0038027, HMDB0038122, HMDB0038144, HMDB0038178, HMDB0039525, HMDB0039688, HMDB0040712, HMDB02352, HMDB30891, HMDB30916, HMDB30920, HMDB31348, HMDB32048, HMDB32292, HMDB32681, HMDB33083, HMDB33104, HMDB34714, HMDB34722, HMDB35032, HMDB35040, HMDB35127, HMDB35147, HMDB35301, HMDB35648, HMDB35827, HMDB36446, HMDB36549, HMDB36668, HMDB36680, HMDB36687, HMDB37393, HMDB37906, HMDB38027, HMDB38122, HMDB38144, HMDB38178, HMDB39525, HMDB39688, HMDB40712	Capsidiol	0,05519752	0,741198383	0,869885586
237,1117307	HMDB0033191, HMDB0034046, HMDB33191, HMDB34046	2,2,4,4,-Tetramethyl-6-(1-oxopropyl)-1,3,5-cyclohexanetrione	-0,104426493	0,330362247	0,594134913
237,1489524	HMDB0038256, HMDB0112083, HMDB38256	Geranyl acetoacetate	-0,193556533	0,172747088	0,408226182

m/z	HMDB ID alternatives	Suggested annotation	*Comparison [vegan] - [omnivore]* Fold Change (log2)	p-value	p-value (BH adj.)
237,1852064	HMDB0029350, HMDB0030428, HMDB0031383, HMDB0031384, HMDB0032054, HMDB0032209, HMDB0032291, HMDB0032343, HMDB0033228, HMDB0034673, HMDB0035287, HMDB0035887, HMDB0036032, HMDB0036730, HMDB0036880, HMDB0037195, HMDB0037279, HMDB0037284, HMDB0038136, HMDB0038138, HMDB0038196, HMDB0038197, HMDB0038247, HMDB0038248, HMDB0038250, HMDB0039130, HMDB0039745, HMDB0040273, HMDB0040281, HMDB0041589, HMDB29350, HMDB30428, HMDB31383, HMDB31384, HMDB32054, HMDB32209, HMDB32291, HMDB32343, HMDB33228, HMDB34673, HMDB35287, HMDB35887, HMDB36032, HMDB36730, HMDB36880, HMDB37195, HMDB37279, HMDB37284, HMDB38136, HMDB38138, HMDB38196, HMDB38197, HMDB38247, HMDB38248, HMDB38250, HMDB39130, HMDB39745, HMDB40273, HMDB40281, HMDB41589	Geranyl 3-methylbutanoate	0,126383091	0,637455495	0,801639866
237,2214887	HMDB0034181, HMDB0037544, HMDB0060482, HMDB34181, HMDB37544, HMDB60482	3-Methylcyclopentadecano	0,022547702	0,691758606	0,843655252
238,0532736	HMDB0042011, HMDB42011	S-Phenylmercapturic acid	-0,07410097	0,986880991	0,99357422
238,0918056	HMDB0032293, HMDB32293	N-Gluconyl ethanolamine	-0,061297317	0,907941892	0,961999186
239,0585308	HMDB0033071, HMDB33071	Propyl 1-(propylsulfinyl)propyl disulfide	-0,1222663	0,1443667	0,368092874
239,0913802	HMDB0030254, HMDB0038627, HMDB0041756, HMDB0061112, HMDB0062776, HMDB0125802, HMDB0125804, HMDB0127748, HMDB0127826, HMDB0128672, HMDB0128705, HMDB0128721, HMDB0142074, HMDB30254, HMDB38627, HMDB41756, HMDB61112, HMDB62776	3-(3,4,5-Trimethoxyphenyl)propa acid	-2,767720341	0,002122924	0,019693506
239,1640845	HMDB0032369, HMDB32369	L-Menthyl acetoacetate	0,401587397	0,334030168	0,594134913
239,200383	HMDB0029765, HMDB0032347, HMDB0034455, HMDB0036426, HMDB0037190, HMDB0037211, HMDB0037229, HMDB0038954, HMDB0039715, HMDB0040997, HMDB29765, HMDB32347, HMDB34455, HMDB36426, HMDB37190, HMDB37211, HMDB37229, HMDB38954, HMDB39715, HMDB40997	C15:1	-0,526772191	0,052181088	0,185722077
239,2363498	HMDB0001551, HMDB0031052, HMDB01551, HMDB31052	Palmitaldehyde	-0,272328087	0,005341596	0,038815596
241,1795381	HMDB0010730, HMDB0030982, HMDB0032370, HMDB10730, HMDB30982, HMDB32370	3-Oxotetradecanoic acid	0,217736352	0,738098776	0,867582504
242,0779258	HMDB0000089, HMDB00089, HMDB0015122, HMDB0060478, HMDB15122, HMDB30167, HMDB60478	Cytidine	0,022250112	0,277294871	0,542154994
242,1416775	HMDB0002366, HMDB02366	Tiglylcarnitine	-0,101229997	0,796012143	0,897959364
242,1750276	HMDB0013286, HMDB13286	N-Undecanoylglycine	-0,418657584	0,505761522	0,726563505
243,0317906	HMDB0059983, HMDB0133637, HMDB0133640, HMDB0133642, HMDB0133644, HMDB0133646, HMDB0133648, HMDB0133649, HMDB0133651, HMDB0133652, HMDB0133656, HMDB0133658, HMDB0133734, HMDB0133735, HMDB0133737, HMDB0133738, HMDB0133740, HMDB0133741, HMDB0133745, HMDB0133766, HMDB0133768, HMDB0133770, HMDB0133772, HMDB0133774, HMDB0135245, HMDB0135653, HMDB59983	4-phenylbutanic acid-O-sulphate	0,025899213	0,428114229	0,666635014
243,2183893	HMDB0001186, HMDB01186	N1-Acetylspermine	-0,512564243	8,60815E-06	0,000227464
244,1365095	HMDB0059773, HMDB59773	S-3-oxodecanoyl cysteamine	0,072683726	0,598157494	0,770448058
245,0124571	HMDB0094710, HMDB0125171, HMDB0128001, HMDB0130016, HMDB0134064, HMDB0134069, HMDB0134071, HMDB0134074, HMDB0134080, HMDB0134083, HMDB94710	3-[3-(Sulfooxy)phenyl]propar acid	0,080025474	0,603269668	0,773604634
245,0463487	HMDB0034312, HMDB0041525, HMDB34312, HMDB41525	Isopimpinellin	0,347917127	0,202767503	0,446844947
245,0463487	HMDB0032077, HMDB32077	Dibenzyl disulfide	0,347917127	0,202767503	0,446844947
245,0764161	HMDB0000497, HMDB0011160, HMDB0028754, HMDB00497, HMDB11160, HMDB28754	5,6-Dihydrouridine	0,104885502	0,084434643	0,253013775
245,1132113	HMDB0011166, HMDB0011172, HMDB0028756, HMDB0028757, HMDB0028832, HMDB0028903, HMDB0028925, HMDB0029162, HMDB0059717, HMDB11166, HMDB11172, HMDB28756, HMDB28757, HMDB28832, HMDB28903, HMDB28925, HMDB29162, HMDB59717	L-beta-aspartyl-L-leucine	0,005254942	0,759216889	0,878033325
245,1385788	HMDB0000413, HMDB0031696, HMDB00413, HMDB31696	3-Hydroxydodecanedioic acid	0,227204062	0,237474655	0,490705922
245,1531152	HMDB0036207, HMDB0037705, HMDB0041250, HMDB36207, HMDB37705, HMDB41250	alpha-Amylcinnamyl acetate	0,190672271	0,572303143	0,762286527
246,0741803	HMDB0028727, HMDB0028748, HMDB28727, HMDB28748	Asparaginyl-Aspartate	-0,021066479	0,792813954	0,897391148
246,1099574	HMDB0028807, HMDB0029059, HMDB0029075, HMDB28807, HMDB29059, HMDB29075	Glutaminylthreonine	-0,124877052	0,530142221	0,744418706
246,9916775	HMDB0041788, HMDB0125151, HMDB0140929, HMDB41788	Vanillic acid 4-sulfate	0,20552292	0,111514827	0,301765788
247,0297892	HMDB0029229, HMDB0128019, HMDB29229	4-Methoxyphenylethanol sulfate	0,004140001	0,713181035	0,857784638
247,0581511	HMDB0011163, HMDB0028749, HMDB11163, HMDB28749	L-beta-aspartyl-L-aspartic acid	0,056940577	0,284899054	0,547206994
247,1125981	HMDB0028986, HMDB0029133, HMDB28986, HMDB29133	Methionyl-Valine	0,07008383	0,091537258	0,266958156
247,1690892	HMDB0032228, HMDB0036221, HMDB0036640, HMDB0037713, HMDB0037719, HMDB32228, HMDB36221, HMDB36640, HMDB37713, HMDB37719	C16:4	0,064551039	0,482286171	0,709204926
248,0229573	HMDB0002062, HMDB02062, HMDB06346	Norepinephrine sulfate	0,296794647	0,127302246	0,33235796
248,0486521	HMDB0015028, HMDB15028	Sulfapyridine	0,060899575	0,855945277	0,93241486

m/z	HMDB ID alternatives	Suggested annotation	Comparison [vegan] - [omnivore] Fold Change (log2)	p-value	p-value (BH adj.)
248,0793668	HMDB0001526, HMDB0006878, HMDB01526, HMDB06878, HMDB06951	S-Acetyldihydrolipoamide	-0,147754827	0,857565043	0,93241486
249,1117246	HMDB0002012, HMDB0032116, HMDB0038804, HMDB0094669, HMDB0133182, HMDB0134155, HMDB0135907, HMDB02012, HMDB32116, HMDB38804, HMDB94669	Ubiquinone-1	0,279832045	0,220499849	0,470451297
249,1489996	HMDB0015371, HMDB0030917, HMDB0031901, HMDB0034721, HMDB0035117, HMDB0035137, HMDB0035148, HMDB0035358, HMDB0035760, HMDB0035798, HMDB0036036, HMDB0036037, HMDB0036550, HMDB0036563, HMDB0036664, HMDB0037064, HMDB0037529, HMDB0037559, HMDB0039156, HMDB0039635, HMDB0039644, HMDB0040754, HMDB0061839, HMDB15371, HMDB30917, HMDB31901, HMDB34721, HMDB35117, HMDB35137, HMDB35148, HMDB35358, HMDB35760, HMDB35798, HMDB36036, HMDB36037, HMDB36550, HMDB36563, HMDB36664, HMDB37064, HMDB37529, HMDB37559, HMDB39156, HMDB39635, HMDB39644, HMDB40754, HMDB61839	Gemfibrozil	0,00326497	0,946465924	0,977865267
249,1850046	HMDB0035293, HMDB0037631, HMDB0037712, HMDB35293, HMDB37631, HMDB37712	C16:3	0,415451301	0,16649598	0,396678946
251,046557	HMDB0030228, HMDB30228	Nigakinone	0,172914718	0,383231745	0,636331303
251,076671	HMDB0000071, HMDB00071, HMDB0029956, HMDB29956	Deoxyinosine	0,111481695	0,591582982	0,766508708
251,1029212	HMDB0013940, HMDB0013941, HMDB0028699, HMDB0029004, HMDB0029046, HMDB0029098, HMDB13940, HMDB13941, HMDB28699, HMDB29004, HMDB29046, HMDB29098	3”-Hydroxyhexobarbital	0,077500246	0,578389079	0,762373662
251,1272357	HMDB0033197, HMDB0036047, HMDB0059661, HMDB33197, HMDB36047, HMDB59661	2,2,4,4-Tetramethyl-6-(1-oxobutyl)-1,3,5-cyclohexanetrione	0,260656805	0,059671948	0,201681933
253,0711585	HMDB0029438, HMDB29438	L-Phosphoarginine	0,094197141	0,36863915	0,624181241
253,0711585	HMDB0128666, HMDB0128669, HMDB0128671, HMDB0128675, HMDB0137285, HMDB0142077	3-(6,7-dimethoxy-2H-1,3-benzodioxol-5-yl)-3-hydroxypropanal	0,094197141	0,36863915	0,624181241
253,081893	HMDB0029445, HMDB0060669, HMDB0060670, HMDB29445, HMDB60669, HMDB60670	L-Agaridoxin	0,080129343	0,567294628	0,762286527
253,0933971	HMDB0006790, HMDB0038664, HMDB06790, HMDB06864, HMDB06934, HMDB38664	Galactosylglycerol	0,002653569	0,668742712	0,8259825
253,0933971	HMDB0014789, HMDB14789	Dyphylline	0,002653569	0,668742712	0,8259825
253,1073672	HMDB0032796, HMDB32796	Methyl 3-(2,3-dihydroxy-3-methylbutyl)-4-hydroxybenzoate	-0,400523589	0,066573507	0,214186619
253,1169635	HMDB0014356, HMDB0029390, HMDB0029443, HMDB0033143, HMDB0037817, HMDB14356, HMDB29390, HMDB29443, HMDB33143, HMDB37817	Midodrine	-0,26349032	0,038390841	0,15427103
253,1169635	HMDB0013075, HMDB0028917, HMDB0028939, HMDB0029064, HMDB0029065, HMDB13075, HMDB28917, HMDB28939, HMDB29064, HMDB29065	Spermic acid 2	-0,26349032	0,038390841	0,15427103
253,2176881	HMDB0002186, HMDB0003229, HMDB0010735, HMDB0012328, HMDB0031053, HMDB0031145, HMDB0031711, HMDB0032638, HMDB0033791, HMDB0035877, HMDB0037647, HMDB0038958, HMDB0041422, HMDB02186, HMDB03229, HMDB10735, HMDB12328, HMDB31053, HMDB31145, HMDB31711, HMDB32638, HMDB33791, HMDB35877, HMDB37647, HMDB38958, HMDB41422, HMDB60082	Hypogeic acid	-0,681014006	0,005632289	0,039929723
254,0801225	HMDB0014608, HMDB0029322, HMDB0030215, HMDB0030320, HMDB0038639, HMDB0038640, HMDB0041996, HMDB14608, HMDB29322, HMDB30215, HMDB30320, HMDB38639, HMDB38640, HMDB41996	Ketorolac	0,020400845	0,203437436	0,446844947
255,0651211	HMDB0005760, HMDB0029462, HMDB0029519, HMDB0030808, HMDB0032577, HMDB0033904, HMDB0036457, HMDB0037316, HMDB0041647, HMDB0061744, HMDB0128077, HMDB0129959, HMDB0134575, HMDB0137512, HMDB0137518, HMDB0137526, HMDB0138504, HMDB0141337, HMDB05760, HMDB29462, HMDB29519, HMDB30808, HMDB32577, HMDB33904, HMDB36457, HMDB37316, HMDB41647, HMDB61744	Dihydrodaidzein	-0,358349669	0,019569267	0,099211632
255,0864577	HMDB0141328, HMDB0141330, HMDB0142079, HMDB0142082	3-hydroxy-3-(3,4,5-trimethoxyphenyl)propar acid	-2,318594582	0,006846086	0,045921436
257,0715552	HMDB0028777, HMDB0028882, HMDB28777, HMDB28882	Cysteinyl-Histidine	0,002930821	0,59371383	0,766762104
258,0727347	HMDB0041934, HMDB41934	Mizoribine	-0,08312432	0,901270768	0,960767861
258,1214855	HMDB0028906, HMDB0028928, HMDB28906, HMDB28928	Isoleucyl-Glutamate	0,068336227	0,78198661	0,889038232
259,1447317	HMDB0033481, HMDB0094673, HMDB33481, HMDB94673	Baptifoline	0,180343475	0,555173939	0,757470764
260,0214477	HMDB0062550, HMDB62550	2-Methoxyacetaminophen sulfate	0,134829996	0,576085804	0,762286527
261,0725511	HMDB0011164, HMDB0028815, HMDB0029145, HMDB0030413, HMDB0030419, HMDB11164, HMDB28815, HMDB29145, HMDB30413, HMDB30419	L-beta-aspartyl-L-glutamic acid	-0,654641624	0,001616999	0,016988231
261,1336776	HMDB0031897, HMDB31897	Phaseolic acid	0,072147814	0,153645053	0,380720112
263,1029146	HMDB0004259, HMDB0006344, HMDB0061136, HMDB04259, HMDB06344, HMDB61136	Acetyl-N-formyl-5-methoxykynurenamine	-0,285068356	0,536462338	0,748472253
263,2002728	HMDB0035910, HMDB0036153, HMDB0037218, HMDB0037224, HMDB0037273, HMDB0039630, HMDB35910, HMDB36153, HMDB37218, HMDB37224, HMDB37273, HMDB39630	Cedryl acetate	0,459182578	0,027404928	0,123819156
263,2358303	HMDB0037543, HMDB37543	(±)-(Z)-2-(5-Tetradecenyl)cyclobutan[illegible]	0,111890089	0,441637252	0,679292816
264,1061255	HMDB0000235, HMDB00235	Thiamine	-0,246049542	0,54035623	0,751710534

m/z	HMDB ID alternatives	Suggested annotation	Comparison [vegan] - [omnivore] Fold Change (log2)	p-value	p-value (BH adj.)
265,1076047	HMDB0040891, HMDB0041582, HMDB0125455, HMDB0133195, HMDB0133196, HMDB0133197, HMDB0133198, HMDB40891, HMDB41582	3”,4”,5”-Trimethoxycinnamyl alcohol acetate	0,187580065	5,99276E-09	6,53211E-07
267,0720348	HMDB0000425, HMDB00425	3-Deoxy-D-glycero-D-galacto-2-nonulosonic acid	-0,164664522	0,871106338	0,943931618
267,1223513	HMDB0038935, HMDB0061643, HMDB38935, HMDB61643	Kamahine C	-0,328891832	0,008085103	0,052613508
267,1378165	HMDB0014400, HMDB14400	Diethylstilbestrol	-0,215804781	0,764784499	0,882132385
267,2321597	HMDB0030997, HMDB0031046, HMDB0040888, HMDB0060038, HMDB0061858, HMDB0062437, HMDB30997, HMDB31046, HMDB40888, HMDB60038, HMDB61858, HMDB62437	Cyclohexaneundecanoic acid	-1,281164927	2,99807E-07	1,53783E-05
269,0874276	HMDB0029955, HMDB29955	D-erythro-L-galacto-Nonulose	-0,034645736	0,280756564	0,546386287
269,210185	HMDB0010733, HMDB0030972, HMDB0030973, HMDB0030978, HMDB10733, HMDB30972, HMDB30973, HMDB30978	3-Oxohexadecanoic acid	0,424792807	0,921179077	0,966761787
269,2476288	HMDB0002259, HMDB0031067, HMDB0034131, HMDB0037372, HMDB0040392, HMDB0040598, HMDB0059870, HMDB0061859, HMDB02259, HMDB31067, HMDB34131, HMDB37372, HMDB40392, HMDB40598, HMDB59870, HMDB61859	Heptadecanoic acid	-1,179016537	3,35421E-10	2,08818E-07
270,1504214	HMDB0060766, HMDB60766	4-Hydroxyatomoxetine	0,20619707	0,067106545	0,214347644
270,2067119	HMDB0013317, HMDB13317	Tridecanoylglycine	1,008844129	0,371410468	0,626440867
270,9908742	HMDB0128634, HMDB0128636, HMDB0128638	[(7-hydroxy-1-oxo-1H-isochromen-3-yl)methoxy]sulfonic acid	-0,186893854	0,223772224	0,473219052
271,0610589	HMDB0002670, HMDB0005897, HMDB0029631, HMDB0030873, HMDB0031824, HMDB0033308, HMDB0033928, HMDB0034002, HMDB0034105, HMDB0037254, HMDB0038549, HMDB0041656, HMDB0041698, HMDB0041704, HMDB0125364, HMDB0125365, HMDB0126021, HMDB0126022, HMDB0128074, HMDB0128075, HMDB0129341, HMDB0129965, HMDB0130173, HMDB0130182, HMDB0133545, HMDB0133993, HMDB0133999, HMDB0134891, HMDB0134892, HMDB0137525, HMDB0137528, HMDB0137531, HMDB0138088, HMDB0138618, HMDB02670, HMDB05897, HMDB29631, HMDB30873, HMDB31824, HMDB33308, HMDB33928, HMDB34002, HMDB34105, HMDB37254, HMDB38549, HMDB41656, HMDB41698, HMDB41704	Naringenin	-0,186332577	0,917569438	0,96534228
271,1909263	HMDB0035918, HMDB35918	7(14)-Bisabolene-2,3,10,11-tetrol	0,124541368	0,934411963	0,975689772
271,2260028	HMDB0010734, HMDB0031057, HMDB0061658, HMDB0112184, HMDB0112185, HMDB0112186, HMDB0112187, HMDB0112188, HMDB0112189, HMDB0112190, HMDB0112191, HMDB0112192, HMDB10734, HMDB31057, HMDB61658	(R)-3-Hydroxy-hexadecanoic acid	0,026248531	0,903303485	0,961759022
272,1283697	HMDB0030187, HMDB0038834, HMDB30187, HMDB38834	(E,E)-Piperlonguminine	0,079260893	0,748768646	0,872880287
272,1717524	HMDB0028722, HMDB0029121, HMDB28722, HMDB29121	Arginyl-Valine	-0,096376426	0,31929356	0,583131918
272,1877682	HMDB0013238, HMDB13238	Heptanoylcarnitine	0,13319134	0,807748022	0,905342256
273,0056767	HMDB0029200, HMDB0041748, HMDB0127955, HMDB0131141, HMDB29200, HMDB41748	Ferulic acid 4-sulfate	0,292740786	0,119872456	0,317716661
273,0439168	HMDB0059981, HMDB0126242, HMDB0126373, HMDB0133253, HMDB0133255, HMDB0133259, HMDB0135676, HMDB0135698, HMDB0135699, HMDB59981	4-Hydroxy-5-(phenyl)-valeric acid-O-sulphate	0,20051331	0,024897401	0,11609911
273,0965026	HMDB0028833, HMDB28833	Glutamyl-Gamma-glutamate	0,212537094	0,830309841	0,919098001
273,1697661	HMDB0000394, HMDB00394	3-Hydroxytetradecanedioic acid	0,194957878	0,510621718	0,729937931
274,1040505	HMDB0005766, HMDB0011738, HMDB0028796, HMDB0028817, HMDB0029147, HMDB05766, HMDB11738, HMDB28796, HMDB28817, HMDB29147	Norophthalmic acid	0,13906121	0,964017197	0,984003393
274,1402368	HMDB0003869, HMDB0004207, HMDB0028824, HMDB0029154, HMDB03869, HMDB04207, HMDB28824, HMDB29154	Epsilon-(gamma-Glutamyl)-lysine	0,276349956	0,98942505	0,99357422
275,0190702	HMDB0034454, HMDB34454	5-(4-Acetoxy-1-butynyl)-2,2”-bithiophene	0,095815872	0,56743931	0,762286527
275,087692	HMDB0011737, HMDB0028818, HMDB0029148, HMDB0042036, HMDB11737, HMDB28818, HMDB29148, HMDB42036	gamma-Glutamylglutamic acid	-0,002177585	0,585330377	0,762941837
275,1015421	HMDB0011106, HMDB11106	5-Amino-6-ribitylamino uracil	0,002876403	0,582625048	0,762836399
275,1665717	HMDB0015586, HMDB0031463, HMDB0038938, HMDB0038994, HMDB0040375, HMDB0041201, HMDB0137236, HMDB0137237, HMDB15586, HMDB31463, HMDB38938, HMDB38994, HMDB40375, HMDB41201	Cyclandelate	-0,282522863	0,470093611	0,700188213
275,2001286	HMDB0002697, HMDB0004590, HMDB0005886, HMDB0006547, HMDB0032464, HMDB0032672, HMDB0034382, HMDB0062677, HMDB02697, HMDB04590, HMDB05886, HMDB06547, HMDB32464, HMDB32672, HMDB34382, HMDB62677	19-Norandrosterone	0,421104095	0,261324637	0,51907764
275,2363095	HMDB0005830, HMDB05830	5a-Androstan-3b-ol	0,61343957	1,47855E-06	6,06903E-05
276,019292	HMDB0002028, HMDB02028	DOPA sulfate	0,097948316	0,910751085	0,962636298

m/z	HMDB ID alternatives	Suggested annotation	Comparison [vegan] - [omnivore] Fold Change (log2)	p-value	p-value (BH adj.)
276,0427675	HMDB0060008, HMDB0060009, HMDB0060010, HMDB60008, HMDB60009, HMDB60010	N-(2-hydroxymethyl-3-chlorophenyl)anthranilic acid	-0,005254267	0,50116533	0,722211629
276,1020823	HMDB0028803, HMDB0028971, HMDB0028987, HMDB28803, HMDB28971, HMDB28987	Glutaminylmethionine	0,024932305	0,876569138	0,948347752
276,1020823	HMDB0033355, HMDB0034110, HMDB33355, HMDB34110	Dehydroaporheine	0,024932305	0,876569138	0,948347752
276,1705223	HMDB0041944, HMDB41944	N-Acetylprocainamide	0,067089664	0,928905911	0,972396103
277,1543414	HMDB0013243, HMDB0028914, HMDB0028998, HMDB0062483, HMDB13243, HMDB28914, HMDB28936, HMDB28998, HMDB62483	Leucyl-phenylalanine	0,001059726	0,436397533	0,675744189
277,1806226	HMDB0030801, HMDB0030974, HMDB0031928, HMDB0039251, HMDB0137260, HMDB30801, HMDB30974, HMDB31928, HMDB39251	1-(4-Hydroxy-3-methoxyphenyl)-3-decanone	0,412137073	0,015668236	0,083309158
277,2164727	HMDB0001388, HMDB0003073, HMDB0030962, HMDB0030963, HMDB0030964, HMDB01388, HMDB03073, HMDB30962, HMDB30963, HMDB30964	Alpha-Linolenic acid	0,61275186	0,031282897	0,135714859
278,0722116	HMDB0032162, HMDB32162	Acrylic acid-2-acrylamido-2-methyl propane sulfonic acid copolymer	-0,068844747	0,044477496	0,168598232
278,1229723	HMDB0037844, HMDB37844	N-(1-Deoxy-1-fructosyl)valine	0,063875628	0,564083446	0,762230786
278,1533681	HMDB0013889, HMDB0015273, HMDB13889, HMDB15273	E-10-Hydroxynortriptyline	0,11410013	0,996938914	0,996938914
279,0378276	HMDB0127482, HMDB0127483	6-[(2-carboxyacetyl)oxy]-3,4,5-trihydroxyoxane-2-carboxylic acid	-0,288852198	0,01769873	0,092414924
279,161581	HMDB0030148, HMDB0036979, HMDB0040755, HMDB0041229, HMDB30148, HMDB36979, HMDB40755, HMDB41229	Prehumulinic acid	0,233577923	0,180182305	0,416761193
281,1015697	HMDB0125459, HMDB0125461, HMDB0125462, HMDB0125463	2-[4,6-dihydroxy-3-(4-hydroxy-3-methylbut-2-en-1-yl)-2-methoxyphenyl]acetic acid	0,165176583	0,603027534	0,773604634
281,1377111	HMDB0033375, HMDB0034941, HMDB0034983, HMDB0035846, HMDB0038660, HMDB0038661, HMDB0038989, HMDB0039241, HMDB0041324, HMDB33375, HMDB34941, HMDB34983, HMDB35846, HMDB38660, HMDB38661, HMDB38989, HMDB39241, HMDB41324	Octyl gallate	-0,057287088	0,935409001	0,975689772
281,1750299	HMDB0033630, HMDB33630	Lactapiperanol C	0,160085949	0,769885741	0,883342587
283,0224882	HMDB0032876, HMDB0034042, HMDB0034138, HMDB0037921, HMDB0128435, HMDB0140295, HMDB0140296, HMDB32876, HMDB34042, HMDB34138, HMDB37921	Rhein	0,022758371	0,835275629	0,921212686
283,064838	HMDB0014718, HMDB0014967, HMDB14718, HMDB14967	Mazindol	-0,082219828	0,578774501	0,762373662
283,1024769	HMDB0126522	[(2Z)-2-(phenylmethylidene)hept acid	-0,004983315	0,608118064	0,77753512
283,1430196	HMDB0014947, HMDB14947	Tropicamide	-0,119169858	0,393242214	0,641527233
283,1687653	HMDB0003422, HMDB0030432, HMDB0038050, HMDB03422, HMDB30432, HMDB38050	Boldione	0,028622047	0,911892263	0,96267561
284,0875162	HMDB0005923, HMDB05923	N4-Acetylcytidine	-0,035774814	0,548856636	0,756072946
285,0595876	HMDB0029498, HMDB0041272, HMDB0059998, HMDB0124997, HMDB0125471, HMDB0135181, HMDB29498, HMDB41272, HMDB59998	3”-Methoxyfukiic acid	0,128262933	0,951195571	0,979271001
285,1594462	HMDB0060995, HMDB60995	10-alpha-methoxy-9,10-dihydrolysergol	-0,644749435	0,004083251	0,031307269
285,2061738	HMDB0000672, HMDB0041287, HMDB0059777, HMDB00672, HMDB41287, HMDB59777	Hexadecanedioic acid	-0,077947515	0,16267081	0,391847917
285,2224093	HMDB0000305, HMDB0006216, HMDB0006217, HMDB0006221, HMDB0030403, HMDB00305, HMDB0034735, HMDB0036728, HMDB0036841, HMDB0038709, HMDB03439, HMDB06216, HMDB06217, HMDB06221, HMDB30403, HMDB34735, HMDB36728, HMDB36841, HMDB38709	Vitamin A	0,068869135	0,831615049	0,919098001
286,1620286	HMDB0014578, HMDB0014873, HMDB14578, HMDB14873	Cyproheptadine	-0,355619813	0,022934937	0,111107028
286,2036265	HMDB0000791, HMDB00791, HMDB00834	L-Octanoylcarnitine	0,282700094	0,343341745	0,603009832
286,2385563	HMDB0032358, HMDB32358	Lauroyl diethanolamide	0,483078982	0,02408255	0,114130345
287,0210034	HMDB0029191, HMDB0135678, HMDB0135688, HMDB0135693, HMDB0135695, HMDB0135696, HMDB29191	5”-(3”,4”-Dihydroxyphenyl)-gamma-valerolactone sulfate	0,186279029	0,627226303	0,797290578
287,0497466	HMDB0000788, HMDB00788	Orotidine	0,139914201	0,00596347	0,041385986
287,274455	HMDB0005943, HMDB0040180, HMDB05943, HMDB40180	5b-Pregnanediol	0,169071769	0,367756389	0,623897998
288,1241948	HMDB0041836, HMDB0041955, HMDB41836, HMDB41955	Benzoyl ecgonine	-0,031072178	0,296193093	0,555441671
288,1598712	HMDB0013960, HMDB0014568, HMDB0014712, HMDB0040700, HMDB13960, HMDB14568, HMDB14712, HMDB31435, HMDB40700	Donepezil metabolite M4	-0,12814322	0,156948015	0,382286785

m/z	HMDB ID alternatives	Suggested annotation	Comparison [vegan] - [omnivore] Fold Change (log2)	p-value	p-value (BH adj.)
289,0388116	HMDB0059975, HMDB0059976, HMDB0125823, HMDB0127756, HMDB0127760, HMDB0135701, HMDB0135702, HMDB0135708, HMDB0135711, HMDB0135712, HMDB0135714, HMDB0135715, HMDB59975, HMDB59976	4-Hydroxy-5-(3”-hydroxyphenyl)-valeric acid-3”-O-sulphate	0,062820221	0,183227031	0,417740595
289,1081559	HMDB0038722, HMDB0127764, HMDB38722	5,6-Dihydro-11-methoxyyangonin	-0,188435699	0,323319822	0,586143212
289,1924376	HMDB0013962, HMDB0060962, HMDB13962, HMDB60962	Verapamil metabolite D-617	-0,022386921	0,064775694	0,209979201
289,2149369	HMDB0014347, HMDB14347	Succinylcholine	0,198879072	0,071777212	0,221949393
289,2179037	HMDB0000031, HMDB0000365, HMDB0000490, HMDB0000546, HMDB0002961, HMDB00031, HMDB0003818, HMDB0005849, HMDB0006770, HMDB00365, HMDB0037711, HMDB00490, HMDB00546, HMDB02961, HMDB03818, HMDB05849, HMDB06770, HMDB37711	Androsterone	0,224724548	0,054338716	0,191061938
290,0353149	HMDB0142153	(2S)-2-amino-3-[4-hydroxy-3-(sulfooxy)phenyl]-2-methylpropanoic acid	-0,02292787	0,154136343	0,380756066
291,0184024	HMDB0124954, HMDB0124955, HMDB0127788, HMDB0127789, HMDB0127843, HMDB0127844, HMDB0128005, HMDB0133893	[2-hydroxy-5-(2-hydroxy-3-methoxy-3-oxopropyl)phenyl]oxidar acid	0,142280601	0,107225364	0,295428876
291,0657311	HMDB0039404, HMDB0039405, HMDB39404, HMDB39405	3-Hydroxy-9-(4-hydroxyphenyl)-1H,3H-naphtho[1,8-cd]pyran-1-one	0,852408459	0,073234967	0,224073303
291,1236888	HMDB0030767, HMDB0037774, HMDB0037775, HMDB0094645, HMDB30767, HMDB37774, HMDB37775, HMDB94645	11,12-Dimethoxydihydrokawai	-0,003693719	0,333324294	0,594134913
293,0481373	HMDB0135202	(2-hydroxy-1,2-diphenylethoxy)sulfonic acid	0,04209622	0,550201696	0,756072946
295,2626038	HMDB0013622, HMDB0035575, HMDB13622, HMDB35575	Nonadeca-10(Z)-enoic acid	-0,9958465	4,2573E-07	2,06243E-05
296,0376081	HMDB0000709, HMDB00709	L-Cysteinylglycine disulfide	-0,09328659	0,479861628	0,708846137
296,0868637	HMDB0032367, HMDB32367	L-Menthone 1,2-glycerol ketal	0,153943351	0,235394131	0,48756219
298,2766005	HMDB0000252, HMDB0001480, HMDB0002100, HMDB00252, HMDB01480, HMDB02100	Sphingosine	-0,323288387	1,60078E-06	6,06903E-05
299,0417043	HMDB0035179, HMDB35179	Mumefural	-0,137152338	0,01947109	0,099211632
299,064478	HMDB0029920, HMDB29920	beta-D-3-Ribofuranosyluric acid	0,041532195	0,734951658	0,867529672
299,2226533	HMDB0011531, HMDB0011562, HMDB11531, HMDB11562	MG(0:0/14:1(9Z)/0:0)	0,008553504	0,005456693	0,03900194
300,0376395	HMDB0000781, HMDB0000814, HMDB0000841, HMDB00781, HMDB00814, HMDB00841	Acetylglucosamine sulfate	0,233872575	0,42786068	0,666635014
300,99912	HMDB0002899, HMDB02899	Ellagic acid	-0,008994095	0,461726479	0,692396623
301,1431091	HMDB0000530, HMDB0005056, HMDB0012270, HMDB0014325, HMDB0036472, HMDB00530, HMDB0126616, HMDB05056, HMDB12270, HMDB14325, HMDB36472	6-Ketoestriol	-0,276024869	0,08953446	0,263763679
301,1662708	HMDB0031094, HMDB31094	Glycerol tributanoate	-0,338839949	0,002188181	0,020085197
302,114763	HMDB0013240, HMDB13240	Indoleacetyl glutamine	0,137288021	0,257885359	0,514590464
303,0180352	HMDB0059985, HMDB0059987, HMDB0060020, HMDB0128711, HMDB0128713, HMDB59985, HMDB59987, HMDB60020	5-(3”,4”,5”-Trihydroxyphenyl)-gamma-valerolactone-3”-O-sulphate	-0,065112014	0,664251084	0,822671228
305,0306721	HMDB0059651, HMDB59651	2-(3,4-Dihydroxybenzoyloxy)-4,6-dihydroxybenzoate	0,44064768	0,248140539	0,50335759
305,0484818	HMDB0135218, HMDB0135220, HMDB0135222, HMDB0135224, HMDB0135226, HMDB0135546, HMDB0135548, HMDB0135550	(3-oxo-1,3-diphenylpropoxy)sulfoni acid	0,212062413	0,857435709	0,93241486
305,1744864	HMDB0012799, HMDB0034780, HMDB12799, HMDB34780	5”-Carboxy-gamma-chromanol	0,190834935	0,38542981	0,636543172
306,0756906	HMDB0000125, HMDB00125, HMDB01463, HMDB06960	Glutathione	0,004394038	0,480422095	0,708846137
307,1537429	HMDB0030496, HMDB0035724, HMDB0036489, HMDB30496, HMDB35724, HMDB36489	ACRL Toxin II	-0,141924183	0,989950023	0,99357422
307,1906008	HMDB0029852, HMDB0031471, HMDB0032664, HMDB0033243, HMDB0034781, HMDB0040867, HMDB29852, HMDB31471, HMDB32664, HMDB33243, HMDB34781, HMDB40867	Methylgingerol	0,455997736	0,038153024	0,15402517
307,2269841	HMDB0030960, HMDB0031091, HMDB0031379, HMDB0061646, HMDB0094643, HMDB0112070, HMDB0112090, HMDB0112096, HMDB0112100, HMDB30960, HMDB31091, HMDB31379, HMDB61646, HMDB94643	Obtusilactone A	0,167679006	0,201431307	0,44580736
308,0970676	HMDB0000230, HMDB0000773, HMDB00230, HMDB00773, HMDB00800	N-Acetylneuraminic acid	-0,025442462	0,784614285	0,890864136

m/z	HMDB ID alternatives	Suggested annotation	Comparison [vegan] - [omnivore] Fold Change (log2)	p-value	p-value (BH adj.)
308,9779278	HMDB0011688, HMDB11688	Ribose 1,5-bisphosphate	0,168062626	0,558120332	0,757470764
309,0412599	HMDB0033657, HMDB0035230, HMDB33657, HMDB35230	De-O-methylsterigmatocystin	0,016963658	0,462127104	0,692396623
309,1175285	HMDB0015102, HMDB15102, HMDB61038	Desloratadine	-0,280889773	0,270524193	0,53491405
309,2790445	HMDB0002231, HMDB0034296, HMDB0034451, HMDB0035159, HMDB0035572, HMDB0035573, HMDB0035574, HMDB0037127, HMDB0062436, HMDB02231, HMDB34296, HMDB34451, HMDB35159, HMDB35572, HMDB35573, HMDB35574, HMDB37127, HMDB62436	C20:1	0,08128346	0,945848802	0,977865267
311,0405055	HMDB0013680, HMDB0029276, HMDB13680, HMDB29276, HMDB39236	Caftaric acid	0,001105354	0,407883239	0,65141792
311,1397929	HMDB0013302, HMDB13302	Phenylalanylphenylalani	-0,228888304	0,238181605	0,491003214
311,1677947	HMDB0032549, HMDB32549	N-Undecylbenzenesulfonic acid	-0,074222225	0,470925791	0,700188213
311,2211419	HMDB0003871, HMDB0004706, HMDB0006940, HMDB0010201, HMDB0010208, HMDB0010221, HMDB0040900, HMDB0062281, HMDB0062434, HMDB03871, HMDB04706, HMDB06940, HMDB10201, HMDB10208, HMDB10221, HMDB40900, HMDB62281, HMDB62434	13-L-Hydroperoxylinoleic acid	0,358922474	0,461626518	0,692396623
314,1028482	HMDB0029324, HMDB0136318, HMDB0136320, HMDB0137014, HMDB29324	Citpressine II	-0,153554514	0,212592464	0,458862942
315,2523265	HMDB0011532, HMDB0011563, HMDB0031008, HMDB0059633, HMDB0072849, HMDB0072862, HMDB0072865, HMDB0072878, HMDB11532, HMDB11563, HMDB31008, HMDB59633, HMDB72849, HMDB72862, HMDB72865, HMDB72878	MG(0:0/15:0/0:0)	0,736251243	0,006075007	0,041385986
316,1166184	HMDB0037839, HMDB37839	N-(1-Deoxy-1-fructosyl)histidine	-0,097234043	0,826675393	0,917884016
316,1909378	HMDB0014869, HMDB0015592, HMDB14869, HMDB15592	Nateglinide	-0,265210854	0,013845921	0,07546027
316,211292	HMDB0061635, HMDB61635	3-hydroxynonanoyl carnitine	0,119839458	0,573159799	0,762286527
319,0260863	HMDB0029465, HMDB29465	Erosnin	-0,214971454	0,08928007	0,263763679
319,0958098	HMDB0041463, HMDB0126080, HMDB41463	2,3-Dihydro-2,3-dihydroxy-4-(4-methoxyphenyl)-1H-phenalen-1-one	0,045987975	0,019793138	0,099766568
319,1541927	HMDB0032698, HMDB0041824, HMDB0041838, HMDB0134978, HMDB32698, HMDB41824, HMDB41838	Bis(2-methylpropanoyloxy)-9,10-epoxy-p-mentha-1,3,5-triene	0,158339493	0,728726917	0,864557648
319,2258581	HMDB0002190, HMDB0002232, HMDB0002283, HMDB0003876, HMDB0004264, HMDB0004673, HMDB0004679, HMDB0004680, HMDB0004682, HMDB0005998, HMDB0006111, HMDB0006245, HMDB0010222, HMDB0010409, HMDB0011134, HMDB0011136, HMDB0012508, HMDB0012567, HMDB0012598, HMDB0036262, HMDB0036705, HMDB0036810, HMDB0038520, HMDB0038688, HMDB0038689, HMDB0038694, HMDB0060101, HMDB0062287, HMDB0062294, HMDB0062302, HMDB0062431, HMDB0062433, HMDB0112068, HMDB0112071, HMDB02110, HMDB02190, HMDB02218, HMDB02232, HMDB02283, HMDB03876, HMDB03978, HMDB04264, HMDB04672, HMDB04673, HMDB04679, HMDB04680, HMDB04682, HMDB04698, HMDB05080, HMDB05998, HMDB06111, HMDB06245, HMDB10222, HMDB10409, HMDB11134, HMDB11136, HMDB11138, HMDB11642, HMDB12508, HMDB12567, HMDB12598, HMDB36262, HMDB36705, HMDB36810, HMDB38520, HMDB38688, HMDB38689, HMDB38694, HMDB60101, HMDB62287, HMDB62294, HMDB62302, HMDB62431, HMDB62433	5,6-Epoxy-8,11,14-eicosatrienoic acid	-0,023065197	0,425314801	0,664649654
321,0427431	HMDB0135416, HMDB0135451, HMDB0135454, HMDB0135455, HMDB0135458, HMDB0135459, HMDB0135462, HMDB0141213	[2-hydroxy-5-(3-phenylpropanoyl)phenyl] acid	0,110525327	0,694114374	0,844166993
321,0593739	HMDB0038515, HMDB38515	L-gamma-Glutamyl-S-allylthio-L-cysteine	0,060123806	0,906389969	0,961999186
321,0990185	HMDB0030266, HMDB30266	Berberrubine	-0,031794152	0,151460216	0,380614721
323,0548581	HMDB0029535, HMDB0030588, HMDB0128875, HMDB0129364, HMDB29535, HMDB30588	Grevilline A	-0,005954352	0,761924651	0,879997742
323,1870268	HMDB0033631, HMDB0038580, HMDB33631, HMDB38580	Lactapiperanol D	0,092189363	0,650701804	0,811748173
325,0344817	HMDB0128433, HMDB0128434, HMDB0128436, HMDB0140672, HMDB0140673, HMDB0140674	13,14-dihydroxy-9-oxo-8,17-dioxatetracyclo[8.7.0.0^{2}, 1(10),2(7),3,5,11(16),12, heptaen-5-yl acetate	-0,019660056	0,041896647	0,164049886
325,1833085	HMDB0031031, HMDB0059915, HMDB31031, HMDB59915	2-Dodecylbenzenesulfonic acid	0,029094304	0,287989516	0,549511724
326,0880807	HMDB0010316, HMDB0029344, HMDB10316, HMDB29344	Acetaminophen glucuronide	0,144154374	0,371101997	0,626440867
327,1832369	HMDB0061062, HMDB61062	7-hydroxygranisetron	-0,144346196	0,441696132	0,679292816
327,1832369	HMDB0034579, HMDB34579	8-O-Methyloblongine	-0,144346196	0,441696132	0,679292816
327,2323316	HMDB0002183, HMDB0030053, HMDB0030446, HMDB0035185, HMDB0038908, HMDB02183, HMDB30053, HMDB30446, HMDB35185, HMDB38908	C22:6	-0,815556353	4,48904E-05	0,000889646
328,1554258	HMDB0003601, HMDB0038724, HMDB03601, HMDB38724	(S)-Reticuline	0,256279903	0,392475934	0,641527233
330,115222	HMDB0033665, HMDB33665	N”-Hydroxyneosaxitoxin	0,12890819	0,033589662	0,143415452

m/z	HMDB ID alternatives	Suggested annotation	Comparison [vegan] - [omnivore] Fold Change (log2)	p-value	p-value (BH adj.)
331,1759357	HMDB0029784, HMDB0033019, HMDB0033110, HMDB0033215, HMDB0033220, HMDB0033221, HMDB0033644, HMDB0034772, HMDB0035025, HMDB0035634, HMDB0038747, HMDB0038980, HMDB0038981, HMDB0039055, HMDB0060012, HMDB29784, HMDB33019, HMDB33110, HMDB33215, HMDB33220, HMDB33221, HMDB33644, HMDB34772, HMDB35025, HMDB35634, HMDB38747, HMDB38980, HMDB38981, HMDB39055, HMDB60012	Unshuoside A	-0,000610997	0,00954923	0,058230271
333,0065086	HMDB0133344, HMDB0134560, HMDB0137537	[3-(7-hydroxy-4-oxo-4H-chromen-2-yl)phenyl]oxidanesulfoni acid	1,495444692	0,184826575	0,419710346
333,02888	HMDB0060029, HMDB0128699, HMDB0128707, HMDB0128717, HMDB60029	5-(3”,4”,5”-Trihydroxyphenyl)-gamma-valerolactone-O-methyl-O-sulphate	0,696644402	0,176272487	0,413197872
333,110474	HMDB0034937, HMDB34937	Cappariloside A	-0,044048311	0,156002032	0,382286785
334,1243542	HMDB0000489, HMDB00489, HMDB01515	Aspartylglycosamine	0,013536772	0,557439272	0,757470764
334,3092749	HMDB0031678, HMDB31678	Pipericine	-0,13872926	0,4873861	0,710703477
335,0772864	HMDB0029287, HMDB0029288, HMDB0030654, HMDB0033563, HMDB0033997, HMDB0033999, HMDB0125035, HMDB0125036, HMDB0125038, HMDB0125039, HMDB0125050, HMDB0125051, HMDB0125053, HMDB0125054, HMDB0125065, HMDB0125066, HMDB0125068, HMDB0125069, HMDB0125078, HMDB0125080, HMDB0125083, HMDB0126514, HMDB29287, HMDB29288, HMDB30654, HMDB33563, HMDB33997, HMDB33999	3-Caffeoyl-1,5-quinolactone	0,130391606	0,584999898	0,762941837
335,2204161	HMDB0001085, HMDB0001193, HMDB0002236, HMDB0002656, HMDB0002982, HMDB0004243, HMDB0004244, HMDB0004688, HMDB0004690, HMDB0004692, HMDB0004693, HMDB0004696, HMDB0004699, HMDB0005050, HMDB0005087, HMDB0005088, HMDB0005089, HMDB0010204, HMDB0010211, HMDB0010216, HMDB0010219, HMDB0011135, HMDB0012498, HMDB0012839, HMDB0034686, HMDB0039171, HMDB0039441, HMDB0060103, HMDB0060104, HMDB0060105, HMDB0062409, HMDB0062479, HMDB0062614, HMDB0062615, HMDB0062619, HMDB0062687, HMDB0062688, HMDB0062749, HMDB01085, HMDB01193, HMDB02236, HMDB02656, HMDB02982, HMDB04243, HMDB04244, HMDB04683, HMDB04688, HMDB04690, HMDB04692, HMDB04693, HMDB04696, HMDB04699, HMDB05050, HMDB05072, HMDB05087, HMDB05088, HMDB05089, HMDB06501, HMDB10204, HMDB10211, HMDB10216, HMDB10218, HMDB10219, HMDB11135, HMDB12498, HMDB12839, HMDB12840, HMDB34686, HMDB39171, HMDB39441, HMDB60103, HMDB60104, HMDB60105, HMDB62409, HMDB62479, HMDB62614, HMDB62615, HMDB62619, HMDB62687, HMDB62688, HMDB62749	Leukotriene B4	0,461100334	0,135357461	0,35128484
337,0372638	HMDB0134587, HMDB0135131, HMDB0135370, HMDB0135374, HMDB0135378, HMDB0135421, HMDB0135425, HMDB0135430, HMDB0135433, HMDB0135434, HMDB0135439, HMDB0135440, HMDB0135445	5-[(E)-2-(3,5-dihydroxyphenyl)ethenyl 2-methoxyphenyloxidanesu acid	0,88473281	0,20943955	0,455439619
337,310462	HMDB0002068, HMDB0002884, HMDB0031013, HMDB0032470, HMDB0035566, HMDB0035567, HMDB0035568, HMDB0035569, HMDB0062659, HMDB02068, HMDB02884, HMDB31013, HMDB32470, HMDB35566, HMDB35567, HMDB35568, HMDB35569, HMDB62659	Erucic acid	0,022661728	0,589543199	0,766142577
338,0376237	HMDB0006273, HMDB0012268, HMDB06273, HMDB12268	5-amino-1-(5-phospho-D-ribosyl)imidazole-4-carboxylate	0,213674871	0,333957669	0,594134913
339,2073096	HMDB0030359, HMDB30359	14,19-Dihydroaspidospermatin	-0,010497598	0,925904177	0,9704188
339,2333153	HMDB0033786, HMDB0039762, HMDB33786, HMDB39762	Plastoquinone 3	-0,299415655	0,575621228	0,762286527
340,1245347	HMDB0006591, HMDB0011668, HMDB06591, HMDB11668	Lactosamine	-0,050658706	0,639887595	0,801743976
341,0517196	HMDB0129381	6-(2H-1,3-benzodioxole-5-carbonyloxy)-3,4,5-trihydroxyoxane-2-carboxylic acid	0,055630983	0,780076585	0,889038232
341,1082004	HMDB0000048, HMDB0000055, HMDB0000163, HMDB0000186, HMDB0000258, HMDB0000740, HMDB0000975, HMDB0002923, HMDB00048, HMDB00055, HMDB0005826, HMDB0006603, HMDB0006792, HMDB0011740, HMDB0011742, HMDB00163, HMDB00186, HMDB00258, HMDB0029880, HMDB0029882, HMDB0029898, HMDB0029902, HMDB0029919, HMDB0029933, HMDB0033368, HMDB0037138, HMDB0038489, HMDB0039237, HMDB0039727, HMDB0039876, HMDB0041627, HMDB0060068, HMDB00740, HMDB00975, HMDB0125600, HMDB0133305, HMDB02923, HMDB05761, HMDB05826, HMDB06603, HMDB06792, HMDB11740, HMDB11742, HMDB14719, HMDB29880, HMDB29882, HMDB29898, HMDB29902, HMDB29919, HMDB29933, HMDB33368, HMDB37138, HMDB38489, HMDB39237, HMDB39727, HMDB39876, HMDB41627, HMDB60068	Disaccharide	0,055325745	0,4914966	0,715500893
342,2087955	HMDB0030340, HMDB0033959, HMDB30340, HMDB33959	Piperolein B	-0,032640037	0,499467079	0,722211629
343,1674976	HMDB0015118, HMDB0015399, HMDB15118, HMDB15399	Formoterol	0,084163006	0,575642099	0,762286527
343,2389603	HMDB0003166, HMDB0003318, HMDB0006001, HMDB03166, HMDB03318, HMDB06001	16b-Hydroxystanozolol	-0,237912464	0,283507481	0,546386287
345,2081609	HMDB0029561, HMDB0030280, HMDB29561, HMDB30280	(+)-Calycanthine	0,125255584	0,415369139	0,656162843
345,2436798	HMDB0031465, HMDB0033897, HMDB0034944, HMDB0036722, HMDB0041001, HMDB0041302, HMDB31465, HMDB33897, HMDB34944, HMDB36722, HMDB41001, HMDB41302	Methyl-[10]-shogaol	-0,146610002	0,11837064	0,315655041

m/z	HMDB ID alternatives	Suggested annotation	Comparison [vegan] - [omnivore] Fold Change (log2)	p-value	p-value (BH adj.)
347,1142985	HMDB0124870, HMDB0127865	1-(2,4,6-trihydroxyphenyl)-3-(3,4,5-trimethoxyphenyl)propan 1-one	-0,033297857	0,10089197	0,284717793
347,1730862	HMDB0033009, HMDB0033011, HMDB0033219, HMDB0033236, HMDB0033645, HMDB0034786, HMDB0034874, HMDB0039014, HMDB0039469, HMDB0041546, HMDB33009, HMDB33011, HMDB33219, HMDB33236, HMDB33645, HMDB34786, HMDB34874, HMDB39014, HMDB39469, HMDB41546	Foeniculoside VIII	0,125272323	2,89803E-05	0,000665462
348,1912524	HMDB0029225, HMDB29225	Coutaric acid	0,011312301	0,594291957	0,766762104
348,2912184	HMDB0013625, HMDB13625	Dihomo-gamma-Linolenoyl ethanolamide	0,152382708	0,51342065	0,730874126
349,00123	HMDB0037851, HMDB0129972, HMDB0133329, HMDB0133354, HMDB0133378, HMDB0137556, HMDB37851	Apigenin 7-sulfate	1,31597933	0,312780963	0,572993698
349,1662926	HMDB0034915, HMDB0036689, HMDB34915, HMDB36689	Calonectrin	0,055866502	0,336503642	0,596404828
350,1486249	HMDB0030388, HMDB30388	9-(beta-D-Ribofuranosyl)zeatin	0,059277432	0,338362604	0,598483146
351,0174915	HMDB0125406, HMDB0128084, HMDB0128088, HMDB0128106, HMDB0129980, HMDB0133387, HMDB0134578, HMDB0141341	(5,7-dihydroxy-4-oxo-2-phenyl-3,4-dihydro-2H-1-benzopyran-3-yl)oxidanesulfonic acid	-0,126001171	0,680703028	0,836018367
351,0281523	HMDB0003173, HMDB03173	Petunidin	-0,01347328	0,532760693	0,74569394
351,2199597	HMDB0001220, HMDB0001320, HMDB0001335, HMDB0001381, HMDB0001403, HMDB0001452, HMDB0001509, HMDB0002122, HMDB0002132, HMDB0002363, HMDB0002400, HMDB0002776, HMDB0004240, HMDB0004385, HMDB0005077, HMDB0005082, HMDB0005844, HMDB0012481, HMDB0012564, HMDB0012587, HMDB0034676, HMDB0035064, HMDB0035338, HMDB0035339, HMDB0036756, HMDB0060041, HMDB0060042, HMDB0062291, HMDB0062798, HMDB01220, HMDB01320, HMDB01335, HMDB01381, HMDB01403, HMDB01452, HMDB01509, HMDB02122, HMDB02132, HMDB02363, HMDB02400, HMDB02776, HMDB04240, HMDB04385, HMDB05077, HMDB05082, HMDB05092, HMDB05844, HMDB11598, HMDB11650, HMDB12481, HMDB12564, HMDB12587, HMDB34676, HMDB35064, HMDB35338, HMDB35339, HMDB36756, HMDB60041, HMDB60042, HMDB62291, HMDB62798	Prostaglandin E2	-0,132497613	0,293548913	0,554284447
352,0847254	HMDB0032802, HMDB32802	4-Hydroxybenzyl isothiocyanate 4’”’-acetylrhamnoside	0,147743528	0,871404762	0,943931618
353,032628	HMDB0059996, HMDB0127741, HMDB0129332, HMDB0129986, HMDB0132272, HMDB0133592, HMDB0134595, HMDB0134600, HMDB0134604, HMDB0134610, HMDB0135091, HMDB0135528, HMDB0135529, HMDB59996	Dihydronaringenin-O-sulphate	1,699052455	0,127013521	0,33235796
353,341411	HMDB0001160, HMDB0035638, HMDB0035642, HMDB0035649, HMDB0035650, HMDB01160, HMDB35638, HMDB35642, HMDB35649, HMDB35650	C23:0	0,226666581	0,357232791	0,614412217
355,1790919	HMDB0033356, HMDB33356	Xanthoplanine	-0,213761136	0,142853751	0,366377856
355,2115578	HMDB0041044, HMDB41044	5,7-Megastigmadien-9-ol glucoside	-0,233947054	0,047597723	0,174912962
356,1177197	HMDB0031944, HMDB31944	Niazimicin A	0,167354738	0,59111011	0,766508708
357,2245972	HMDB0012983, HMDB12983	Kinetensin 1-3	-0,024468297	0,036737238	0,149114436
357,2805222	HMDB0006322, HMDB0006323, HMDB0035583, HMDB06322, HMDB06323, HMDB12841, HMDB13637, HMDB35583	C24:5	-0,000190808	0,690525415	0,843330758
357,2996626	HMDB0011131, HMDB0011535, HMDB0031075, HMDB0072842, HMDB0072877, HMDB11131, HMDB11535, HMDB31075, HMDB72842, HMDB72877	MG(18:0/0:0/0:0)	0,091354194	0,152888533	0,380720112
358,1137652	HMDB0010336, HMDB0029452, HMDB10336, HMDB29452	Epinephrine glucuronide	-0,306829648	0,045341845	0,168598232
358,2622297	HMDB0013164, HMDB0061638, HMDB13164, HMDB61638	2-Hydroxylauroylcarnitine	0,111187395	0,319940305	0,583131918
359,1497939	HMDB0037235, HMDB0039034, HMDB0039100, HMDB0040311, HMDB0040796, HMDB37235, HMDB39034, HMDB39100, HMDB40311, HMDB40796	Quassinol	0,503362382	0,413056197	0,65407493
361,0416322	HMDB0036932, HMDB0126649, HMDB0126650, HMDB0126651, HMDB0126652, HMDB0126653, HMDB0130446, HMDB0130447, HMDB0130448, HMDB0130449, HMDB0130450, HMDB36932	2-O-Galloylgalactaric acid	-0,26523946	0,399595052	0,64407927
361,0565838	HMDB0128749, HMDB0128750, HMDB0129279, HMDB0130277, HMDB0130281	2-(3,4-dihydroxy-5-methoxyphenyl)-3,5,7-trihydroxy-6-methoxy-4H-chromen-4-one	-0,042644816	0,062034251	0,204664919
362,2708175	HMDB0005085, HMDB0013630, HMDB0013652, HMDB0013659, HMDB05085, HMDB13630, HMDB13652, HMDB13659	Leukotriene B4 dimethylamide	0,416531982	0,062666896	0,204664919
365,0343776	HMDB0126071, HMDB0126072, HMDB0133292, HMDB0133303	6-hydroxy-3-[3-(3-hydroxyphenyl)-3-oxoprop-1-en-1-yl]-2-methoxyphenyloxidanesu acid	0,036173712	0,75373739	0,874014633
365,9797666	HMDB0041985, HMDB41985	Phosalone	0,034502577	0,045173988	0,168598232

m/z	HMDB ID alternatives	Suggested annotation	*Comparison [vegan] - [omnivore]* Fold Change (log2)	p-value	p-value (BH adj.)
367,1047334	HMDB0030669, HMDB0039959, HMDB0039960, HMDB0126418, HMDB0126419, HMDB0126420, HMDB0126421, HMDB0126422, HMDB0126423, HMDB0127742, HMDB0129399, HMDB0135673, HMDB30669, HMDB39959, HMDB39960	3-O-Feruloylquinic acid	0,131998449	0,355040807	0,614116037
367,1573803	HMDB0001032, HMDB0002833, HMDB0013230, HMDB01032, HMDB02833, HMDB13230	Dehydroepiandrosterone sulfate	-0,207608681	0,779703873	0,889038232
367,1880931	HMDB0114760, HMDB0114764	LPA(a-13:0/0:0)	-0,12100901	0,963442677	0,984003393
368,3149199	HMDB0062341, HMDB62341	N-Stearoyl GABA	-0,146582196	0,846854529	0,927013183
369,0986523	HMDB0031941, HMDB0034119, HMDB0035527, HMDB0036555, HMDB0038398, HMDB0038900, HMDB0038932, HMDB0039731, HMDB0039732, HMDB0132502, HMDB0132503, HMDB0137144, HMDB31941, HMDB34119, HMDB35527, HMDB36555, HMDB38398, HMDB38900, HMDB38932, HMDB39731, HMDB39732	8”-Episesaminone	-0,099654752	0,838037378	0,921212686
371,1497084	HMDB0005789, HMDB0030087, HMDB0030826, HMDB0032733, HMDB0032734, HMDB0035182, HMDB0039066, HMDB0041416, HMDB0124810, HMDB0124811, HMDB0124812, HMDB0124813, HMDB0124815, HMDB0124816, HMDB0124817, HMDB0124828, HMDB0124911, HMDB0124912, HMDB0124913, HMDB0124914, HMDB0124915, HMDB0125800, HMDB0130099, HMDB0130100, HMDB0130288, HMDB0130289, HMDB0130290, HMDB0130291, HMDB0130292, HMDB0133480, HMDB0133481, HMDB0133492, HMDB0133513, HMDB0133514, HMDB0133520, HMDB0133527, HMDB0133537, HMDB05789, HMDB30087, HMDB30826, HMDB32733, HMDB32734, HMDB35182, HMDB39066, HMDB41416	Tetrahydrocurcumin	-0,303076751	0,044005815	0,168598232
371,1753048	HMDB0003134, HMDB03134	Biocytin	-0,011838605	0,758285142	0,878033325
371,2568939	HMDB0013627, HMDB0014629, HMDB0041301, HMDB13627, HMDB14629, HMDB41301	Cervonoyl ethanolamide	0,189226125	0,093059935	0,268702858
372,3282482	HMDB0039738, HMDB39738	Pipereicosalidine	-0,074883648	0,75037936	0,872880287
373,2005505	HMDB0011651, HMDB0015094, HMDB0030456, HMDB0040525, HMDB11651, HMDB15094, HMDB30456, HMDB40525	11beta,20-Dihydroxy-3-oxopregn-4-en-21-oic acid	-0,115937199	0,468512496	0,700188213
373,2751974	HMDB0000308, HMDB00308, HMDB0038522, HMDB0041453, HMDB38522, HMDB41453	3b-Hydroxy-5-cholenoic acid	0,495790127	0,33393231	0,594134913
375,2162198	HMDB0002294, HMDB0003733, HMDB0011652, HMDB0012580, HMDB0012601, HMDB0012638, HMDB0012777, HMDB0012855, HMDB0030147, HMDB0030455, HMDB0040955, HMDB0040956, HMDB02294, HMDB03733, HMDB11652, HMDB12580, HMDB12601, HMDB12638, HMDB12777, HMDB12855, HMDB30147, HMDB30455, HMDB40955, HMDB40956	Resolvin D2	0,052051556	0,700336904	0,847843971
375,2906519	HMDB0000381, HMDB0000713, HMDB0000717, HMDB0000761, HMDB0002431, HMDB0002492, HMDB00381, HMDB00713, HMDB00717, HMDB00761, HMDB02431, HMDB02492	Allolithocholic acid	0,112748912	0,558547822	0,757470764
377,1937339	HMDB0003933, HMDB03933	Pentosidine	0,02882108	0,183480101	0,417740595
377,2122245	HMDB0031978, HMDB31978	Methyl (9Z)-6”-oxo-6,6”-diapo-6-carotenoate	0,178133798	0,704724439	0,851135333
377,2350817	HMDB0035508, HMDB35508	Annoglabasin F	0,08188867	0,837447666	0,921212686
378,2391411	HMDB0000277, HMDB00277, HMDB0059630, HMDB0062531, HMDB59630, HMDB62531	Sphingosine 1-phosphate	-0,061235705	0,651920242	0,812106359
378,2648753	HMDB0002304, HMDB02304	Leukotriene B4 ethanolamide	0,193104584	0,012184434	0,070363088
379,2029308	HMDB0015696, HMDB15696	Bopindolol	0,292433864	0,119249401	0,317028895
381,0050566	HMDB0060600, HMDB60600	Olsalazine-O-sulfate	0,095397488	0,515511664	0,730874126
381,3001521	HMDB0011544, HMDB0011574, HMDB0035959, HMDB0036865, HMDB11544, HMDB11574, HMDB35959, HMDB36865	MG(0:0/20:2(11Z,14Z)/(	0,139453239	0,198784027	0,44219304
382,0274532	HMDB0015427, HMDB15427	Ceftizoxime	-0,194357773	0,096253082	0,272508727
383,0626471	HMDB0040571, HMDB0129411, HMDB0129412, HMDB40571	2-O-Feruloylhydroxycitric acid	0,329608654	0,051664463	0,184636933
383,1510594	HMDB0035427, HMDB0040809, HMDB0040834, HMDB0041100, HMDB0041417, HMDB35427, HMDB40809, HMDB40834, HMDB41100, HMDB41417	Glicoisoflavanone	-0,037763844	0,847281545	0,927013183
383,1954507	HMDB0061005, HMDB61005	repaglinide aromatic amine	0,00668308	0,957678131	0,982465095
383,2071295	HMDB0012643, HMDB0036010, HMDB12643, HMDB36010	20-Trihydroxy-leukotriene-B4	-0,020284511	0,970090331	0,985816982
383,2348796	HMDB0033523, HMDB33523	(-)-Pyrifolidine	-0,0876253	0,521992505	0,735917092
383,3513578	HMDB0039540, HMDB39540	Cerebronic acid	0,074433559	0,68231631	0,836821128
384,2743126	HMDB0013330, HMDB13330	3-Hydroxy-cis-5-tetradecenoylcarnitine	0,164509517	0,044259751	0,168598232
384,3102698	HMDB0062517, HMDB62517	Pentadecanoylcarnitine	0,021182479	0,830859987	0,919098001
385,1656954	HMDB0030810, HMDB0035404, HMDB0035405, HMDB30810, HMDB35404, HMDB35405	Porson	0,139202277	1,48495E-09	2,9748E-07
385,33077	HMDB0011147, HMDB0011542, HMDB0011572, HMDB0072854, HMDB0072859, HMDB11147, HMDB11542, HMDB11572, HMDB72854, HMDB72859	DG(18:0e/2:0/0:0)	0,26456799	0,118256574	0,315655041
387,2013847	HMDB0031676, HMDB0036318, HMDB0036846, HMDB0038306, HMDB0040614, HMDB31676, HMDB36318, HMDB36846, HMDB38306, HMDB40614	5a,6a-Epoxy-7E-megastigmene-3a,9e-diol 3-glucoside	-0,062304978	0,334251902	0,594134913
387,3274266	HMDB0036371, HMDB36371	3-Hydroxy-1-phenyl-1-eicosanone	0,244746949	0,225336354	0,473219052
391,1527045	HMDB0030655, HMDB30655	Calabaxanthone	0,055432978	0,171488056	0,406352133

m/z	HMDB ID alternatives	Suggested annotation	*Comparison [vegan] - [omnivore]* Fold Change (log2)	p-value	p-value (BH adj.)
391,261884	HMDB0039019, HMDB39019	3-Hydroxy-10”-apo-b,y-carotenal	0,535933755	0,002957054	0,025279914
393,1347415	HMDB0029510, HMDB0034436, HMDB29510, HMDB34436	Garcinone B	0,21310786	0,162263022	0,391847917
395,0098337	HMDB0128825, HMDB0131940, HMDB0133829	[3-(3,5,7-trihydroxy-6-methoxy-4-oxo-4H-chromen-2-yl)phenyl]oxidanesulfoni acid	0,128478276	0,001283589	0,014168221
395,1532407	HMDB0030900, HMDB30900	Eremopetasitenin A2	0,203185598	0,386551901	0,637189523
395,1842362	HMDB0039042, HMDB0040305, HMDB0040312, HMDB39042, HMDB40305, HMDB40312	(3”x,5”a,9”x,10”b)-O-(3-Hydroxy-6-oxo-7-drimen-11-yl)umbelliferone	-0,058938641	0,581428908	0,762836399
397,9882749	HMDB0000793, HMDB00793	Monoiodothyronine	-0,108560865	0,12182835	0,321922185
399,1667996	HMDB0030975, HMDB30975	Corchoionoside B	0,037831075	0,778491932	0,889038232
399,2518514	HMDB0012518, HMDB0030065, HMDB12518, HMDB30065	11”-Carboxy-gamma-tocotrienol	0,266373405	0,090671533	0,265936044
401,208407	HMDB0060583, HMDB60583	Trandolaprilat	0,193224207	0,418043023	0,658002736
401,2929877	HMDB0029887, HMDB29887	Sorbitan palmitate	0,253749109	0,072835094	0,223634515
401,3412194	HMDB0001419, HMDB0001496, HMDB0002103, HMDB0002902, HMDB0004035, HMDB0006119, HMDB0006247, HMDB0006283, HMDB0006892, HMDB0011643, HMDB0013643, HMDB0029320, HMDB0034254, HMDB0035681, HMDB0038959, HMDB0039868, HMDB0039869, HMDB0039870, HMDB0059592, HMDB0062328, HMDB01419, HMDB01496, HMDB02103, HMDB02902, HMDB04035, HMDB06119, HMDB06247, HMDB06283, HMDB06892, HMDB11643, HMDB13643, HMDB29320, HMDB34254, HMDB35681, HMDB38959, HMDB39868, HMDB39869, HMDB39870, HMDB59592, HMDB62328	24-Hydroxycholesterol	0,530933376	0,000600422	0,007862977
403,3560384	HMDB0006893, HMDB0031035, HMDB0036844, HMDB06893, HMDB31035, HMDB36844	3a,7a-Dihydroxy-5b-cholestane	0,438112287	0,090881813	0,265936044
405,1719886	HMDB0030628, HMDB0030778, HMDB0030779, HMDB0030780, HMDB0033099, HMDB0033536, HMDB0033611, HMDB0034269, HMDB0034917, HMDB0037444, HMDB0038875, HMDB0038876, HMDB30628, HMDB30778, HMDB30779, HMDB30780, HMDB33099, HMDB33536, HMDB33611, HMDB34269, HMDB34917, HMDB37444, HMDB38875, HMDB38876	Rubraflavone A	0,307586783	1,70573E-09	2,9748E-07
405,2626856	HMDB0000391, HMDB0000400, HMDB0000502, HMDB0033399, HMDB0035288, HMDB00391, HMDB00400, HMDB00502, HMDB0062680, HMDB33399, HMDB35288, HMDB62680	7-Ketodeoxycholic acid	0,406043752	0,501903953	0,722211629
405,33719	HMDB0112081	3,4-Dimethyl-5-pentyl-2-furanpentadecanoic acid	0,319800122	0,164656994	0,393372324
407,1460453	HMDB0060830, HMDB60830	Melatonin glucuronide	0,042813473	0,077327591	0,234130761
411,1233944	HMDB0013842, HMDB13842	O-Deethylated candesartan	-0,196064254	0,150702615	0,379805436
411,157257	HMDB0033467, HMDB33467	Dicaffeoylputrescine	-0,46457031	0,033677322	0,143415452
411,1738874	HMDB0014725, HMDB14725	Cinalukast	-0,250289574	0,14832131	0,374887486
411,1862219	HMDB0041512, HMDB41512	Isopentyl gentiobioside	-0,098376627	0,658901973	0,818465129
411,2403395	HMDB0003752, HMDB03752	LysoPC(10:0)	0,192424807	0,057863583	0,19709783
411,3464245	HMDB0011552, HMDB0011582, HMDB11552, HMDB11582	MG(0:0/22:1(13Z)/0:0)	0,889909209	0,047915161	0,174912962
413,2693583	HMDB0012516, HMDB0030041, HMDB0030140, HMDB12516, HMDB30041, HMDB30140	11”-Carboxy-alpha-tocotrienol	0,512943021	0,004128826	0,031307269
413,3263055	HMDB0092907, HMDB0092908, HMDB0092909, HMDB0092910, HMDB0092911, HMDB0092912, HMDB0093139, HMDB0093140, HMDB0093141, HMDB0093142, HMDB0093143, HMDB0093144, HMDB92907, HMDB92908, HMDB92909, HMDB92910, HMDB92911, HMDB92912, HMDB93139, HMDB93140, HMDB93141, HMDB93142, HMDB93143, HMDB93144	DG(8:0/13:0/0:0)	0,849498815	0,056506153	0,194843845
415,3211291	HMDB0000430, HMDB0001420, HMDB0001903, HMDB0002197, HMDB0006226, HMDB0006720, HMDB0012453, HMDB0012457, HMDB0012459, HMDB0015046, HMDB0030024, HMDB0030047, HMDB0030048, HMDB0035300, HMDB00430, HMDB0060133, HMDB0060425, HMDB0060508, HMDB0062401, HMDB0062610, HMDB0062736, HMDB01420, HMDB01903, HMDB02197, HMDB06226, HMDB06720, HMDB12453, HMDB12457, HMDB12459, HMDB15046, HMDB30024, HMDB30047, HMDB30048, HMDB35300, HMDB60133, HMDB60425, HMDB60508, HMDB62401, HMDB62610, HMDB62736	24,25-Dihydroxyvitamin D	0,387374839	0,04543645	0,168598232
417,2986018	HMDB0012515, HMDB0039317, HMDB12515, HMDB39317	11”-Carboxy-alpha-chromanol	0,893428112	2,04049E-06	6,59001E-05
417,3168831	HMDB0035686, HMDB35686	beta-Citraurinene	0,830030015	6,0644E-06	0,000170586
418,2954642	HMDB0006463, HMDB0062340, HMDB06463, HMDB62340	Stearidonyl carnitine	0,546099745	4,11203E-06	0,000123644
420,1605268	HMDB0041991, HMDB41991	Pitavastatin	-0,080706511	0,941236254	0,977092874
420,3095449	HMDB0006318, HMDB0006319, HMDB06318, HMDB06319	Gamma-linolenyl carnitine	0,348142501	0,025209738	0,116156048
421,2263588	HMDB0032386, HMDB32386	2-Methylacetophenone	-0,025698624	0,416687007	0,657054376
421,2717009	HMDB0011142, HMDB11142	DHAP(18:0e)	0,098087062	0,528988361	0,743996533

m/z	HMDB ID alternatives	Suggested annotation	Comparison [vegan] - [omnivore] Fold Change (log2)	p-value	p-value (BH adj.)
423,1800759	HMDB0030149, HMDB0030862, HMDB0031743, HMDB0031917, HMDB0036596, HMDB0037097, HMDB0038868, HMDB0041259, HMDB0129603, HMDB0132647, HMDB0133035, HMDB0133036, HMDB0133037, HMDB0133038, HMDB0133039, HMDB0133062, HMDB0133063, HMDB0133064, HMDB0133065, HMDB0133066, HMDB0133068, HMDB0133069, HMDB0133070, HMDB0133071, HMDB0133072, HMDB0134737, HMDB30149, HMDB30862, HMDB31743, HMDB31917, HMDB36596, HMDB37097, HMDB38868, HMDB41259	Glisoflavanone	0,035412137	0,072601134	0,223634515
423,2761677	HMDB0000307, HMDB0000311, HMDB0000316, HMDB0000340, HMDB0000367, HMDB0000377, HMDB0000399, HMDB0000433, HMDB0000437, HMDB0013158, HMDB0013192, HMDB00307, HMDB00311, HMDB00316, HMDB00340, HMDB00342, HMDB00367, HMDB00377, HMDB00399, HMDB00433, HMDB00437, HMDB13158, HMDB13192	1b-Hydroxycholic acid	0,169481617	0,4858406	0,710703477
423,3282422	HMDB0006327, HMDB0038656, HMDB06327, HMDB38656	Alpha-Tocotrienol	0,51453208	0,103909643	0,290414131
424,3804836	HMDB0013310, HMDB13310	Tetracosanoylglycine	0,70489952	0,042168479	0,164155864
425,1094647	HMDB0130070, HMDB0130073, HMDB0130076, HMDB0130077, HMDB0130078, HMDB0130082, HMDB0130085, HMDB0130086, HMDB0130087	6-[3-(1,2-dihydroxybutyl)-1-oxo-1H-isochromen-7-yl]oxy-3,4,5-trihydroxyoxane-2-carboxylic acid	0,167142312	0,457832162	0,691905798
425,1415469	HMDB0003556, HMDB03556	Chitobiose	0,108669536	2,01403E-06	6,59001E-05
427,1832329	HMDB0000851, HMDB00851	Pyridinoline	0,089272724	0,040617254	0,162469015
427,212941	HMDB0030158, HMDB30158	Austalide L	0,225824249	0,571732142	0,762286527
427,306788	HMDB0029886, HMDB0032298, HMDB29886, HMDB32298	Sorbitan oleate	0,492328792	0,068436786	0,214705473
429,0811032	HMDB0033587, HMDB0041717, HMDB0041718, HMDB0127308, HMDB0129981, HMDB0132466, HMDB0132467, HMDB0133342, HMDB0133343, HMDB0134552, HMDB0134553, HMDB0134554, HMDB0134555, HMDB0134556, HMDB0134557, HMDB0134558, HMDB0134559, HMDB0134561, HMDB0134562, HMDB0134563, HMDB0134564, HMDB33587, HMDB41717, HMDB41718	Coumestrin	0,307789354	0,470099256	0,700188213
429,1051474	HMDB0128698, HMDB0128702, HMDB0128706, HMDB0128714	6-[1-(6,7-dimethoxy-2H-1,3-benzodioxol-5-yl)-3-oxopropoxy]-3,4,5-trihydroxyoxane-2-carboxylic acid	-0,12841299	0,111777924	0,301765788
429,3009996	HMDB0011590, HMDB0012458, HMDB0030066, HMDB0034403, HMDB0036249, HMDB0060127, HMDB0060128, HMDB11590, HMDB12458, HMDB30066, HMDB34403, HMDB36249, HMDB60127, HMDB60128	MG(24:6(6Z,9Z,12Z,15Z	0,590756513	0,002824782	0,024388218
429,3166218	HMDB0035640, HMDB35640	Sintaxanthin	0,499273162	0,014330071	0,077613799
429,3729036	HMDB0001893, HMDB0012171, HMDB0030022, HMDB0059642, HMDB0062391, HMDB01893, HMDB12171, HMDB30022, HMDB59642, HMDB62391	Alpha-Tocopherol	-0,097452024	0,015646781	0,083309158
431,2206675	HMDB0062316, HMDB0062320, HMDB0114743, HMDB0114744, HMDB62316, HMDB62320	1-(6Z,9Z,12Z-octadecatrienoyl)-glycero-3-phosphate	0,049344245	0,227331559	0,474056558
431,3149368	HMDB0006228, HMDB0012454, HMDB0030702, HMDB0033769, HMDB0060134, HMDB0062211, HMDB0062399, HMDB0062400, HMDB0062686, HMDB0062712, HMDB06228, HMDB12454, HMDB30702, HMDB33769, HMDB60134, HMDB62211, HMDB62399, HMDB62400, HMDB62686, HMDB62712	24-Hydroxycalcitriol	0,81492011	0,009713956	0,058417721
433,2043793	HMDB0061126, HMDB61126	4,5-Dihydro-drospirenone-3-sulfate	-0,108390811	0,30301867	0,563395054
433,2343739	HMDB0007852, HMDB0007856, HMDB07852, HMDB07856	LPA(0:0/18:2(9Z,12Z))	0,155823057	0,102948975	0,28916431
433,2597393	HMDB0033030, HMDB0040389, HMDB33030, HMDB40389	(12S,15S)-15-O-Demethyl-10,29-dideoxy-11,12-dihydro-striatin C	0,102189008	0,961362788	0,983929989
433,3309576	HMDB0000359, HMDB0003533, HMDB00359, HMDB0062208, HMDB01974, HMDB03533, HMDB06263, HMDB62208	3a,7a-Dihydroxycoprostanic acid	0,671949487	0,012980404	0,073025243
435,2510421	HMDB0007851, HMDB0007855, HMDB0011133, HMDB00443, HMDB07851, HMDB07855, HMDB11133	LPA(0:0/18:1(9Z))	0,103363798	0,304581654	0,563896395
437,1394194	HMDB0129155, HMDB0129156, HMDB0129157, HMDB0129165, HMDB0129166, HMDB0129167, HMDB0129172, HMDB0129173, HMDB0129174, HMDB0129178, HMDB0129182, HMDB0129183, HMDB0129184, HMDB0129189, HMDB0129190, HMDB0129191, HMDB0129195, HMDB0129199, HMDB0135817, HMDB0135818, HMDB0135819, HMDB0135824, HMDB0135825, HMDB0135826, HMDB0135831, HMDB0135832, HMDB0135833	3-(3-hydroxyphenyl)-2-(4-hydroxyphenyl)-4-[(E)-2-(4-hydroxyphenyl)ethenyl]-2,3-dihydro-1-benzofuran-6-ol	0,268862844	0,25029098	0,504050196
437,3257177	HMDB0002126, HMDB02126	27-Nor-5b-cholestane-3a,7a,12a,24,25-pentol	0,961046923	0,006135379	0,041473261
439,1531185	HMDB0014934, HMDB14934	Candesartan	0,042022709	0,898796745	0,959303258
440,1568366	HMDB0032803, HMDB32803	Mandelonitrile rutinoside	-0,035560987	0,343687943	0,603009832

m/z	HMDB ID alternatives	Suggested annotation	*Comparison [vegan] - [omnivore]* Fold Change (log2)	p-value	p-value (BH adj.)
441,1547895	HMDB0128914	1-hydroxy-2-9-[(3-methylbut-2-enoyl)oxy]-2-oxo-2H,8H,9H-furo[2,3-h]chromen-8-ylpropan-2-yl 3-methylbut-2-enoate	0,013078199	0,979115762	0,99162479
441,3563598	HMDB0092917, HMDB0092918, HMDB0092919, HMDB0092920, HMDB0092921, HMDB0092922, HMDB0092967, HMDB0092968, HMDB0092969, HMDB0092970, HMDB0092971, HMDB0092972, HMDB0093145, HMDB0093146, HMDB0093147, HMDB0093148, HMDB0093149, HMDB0093150, HMDB0093427, HMDB0093428, HMDB0093429, HMDB0093430, HMDB0093431, HMDB0093432, HMDB92917, HMDB92918, HMDB92919, HMDB92920, HMDB92921, HMDB92922, HMDB92967, HMDB92968, HMDB92969, HMDB92970, HMDB92971, HMDB92972, HMDB93145, HMDB93146, HMDB93147, HMDB93148, HMDB93149, HMDB93150, HMDB93427, HMDB93428, HMDB93429, HMDB93430, HMDB93431, HMDB93432	DG(8:0/15:0/0:0)	1,086700949	0,056531528	0,194843845
443,2794335	HMDB0000969, HMDB00969	1,25-Dihydroxyvitamin D3-26,23-lactone	1,000153758	0,092206918	0,268014774
443,3891532	HMDB0012160, HMDB0035930, HMDB0036253, HMDB0039752, HMDB0040753, HMDB0062380, HMDB12160, HMDB35930, HMDB36253, HMDB39752, HMDB40753, HMDB62380	4,4-Dimethyl-14alpha-hydroxymethyl-5alpha-cholesta-8-en-3beta-ol	0,659925082	0,000354302	0,005149187
444,2827241	HMDB0012936, HMDB12936	Dynorphin B (10-13)	0,869114693	0,08528081	0,253054245
445,0762825	HMDB0032877, HMDB0041737, HMDB0041738, HMDB0041739, HMDB0041832, HMDB0125397, HMDB0125398, HMDB0125399, HMDB0129970, HMDB0129971, HMDB0130004, HMDB0130202, HMDB0130203, HMDB0132462, HMDB0132464, HMDB0133326, HMDB0133327, HMDB0133328, HMDB0133351, HMDB0133352, HMDB0133353, HMDB0133375, HMDB0133376, HMDB0133377, HMDB0134895, HMDB0134896, HMDB0134897, HMDB32877, HMDB41737, HMDB41738, HMDB41739, HMDB41832	Glucorhein	0,386077496	0,347684936	0,607577683
447,2013332	HMDB0004482, HMDB0006224, HMDB0010317, HMDB0010322, HMDB0010349, HMDB0033273, HMDB0034584, HMDB0037041, HMDB04482, HMDB06224, HMDB06767, HMDB10317, HMDB10322, HMDB10349, HMDB33273, HMDB34584, HMDB37041	2-Methoxyestrone 3-glucuronide	0,244151032	0,301547161	0,561857104
448,3061236	HMDB0000631, HMDB0000637, HMDB0000708, HMDB0006898, HMDB00631, HMDB00637, HMDB00708, HMDB04012, HMDB04013, HMDB06898	Deoxycholic acid glycine conjugate	0,958422302	0,382490157	0,636331303
450,2641995	HMDB0035366, HMDB35366	Cytochalasin Opho	0,134401463	0,361041351	0,617309918
451,1758895	HMDB0041323, HMDB41323	Heteroartonin A	0,032381788	0,728606837	0,864557648
451,3420278	HMDB0000483, HMDB0000513, HMDB0000556, HMDB0000558, HMDB0002180, HMDB0002208, HMDB00483, HMDB00513, HMDB00519, HMDB00556, HMDB00558, HMDB00587, HMDB0062416, HMDB0062421, HMDB02180, HMDB02208, HMDB62416, HMDB62421	5b-Cholestane-3a,7a,12a,23S,25-pentol	0,995746164	0,108324882	0,296110649
452,2774194	HMDB0011473, HMDB0011503, HMDB11473, HMDB11503	LysoPE(0:0/16:0)	-0,138152135	0,622093287	0,791920214
453,3224701	HMDB0002157, HMDB02157	27-Norcholestanehexol	0,870408804	0,020218799	0,100174958
453,3856904	HMDB0040817, HMDB40817	Nb-Arachidoyltryptamine	0,9034081	0,053172965	0,188483031
454,3895434	HMDB0006460, HMDB06460	Arachidyl carnitine	0,766074442	0,044453536	0,168598232
455,1225622	HMDB0060086, HMDB60086	5-S-Glutathionyl-aminochrome reduced	0,075030138	0,948893997	0,979213687
455,1728315	HMDB0036981, HMDB0133936, HMDB0133938, HMDB0133940, HMDB0133942, HMDB0133944, HMDB0133946, HMDB0133952, HMDB0134635, HMDB0134637, HMDB0134639, HMDB0134641, HMDB0134643, HMDB0134645, HMDB0134647, HMDB0134651, HMDB0134653, HMDB0134654, HMDB36981	Atrovirinone	-0,026194242	0,343602187	0,603009832
455,2192972	HMDB0012496, HMDB0062308, HMDB0114748, HMDB12496, HMDB62308	1-Lyso-2-arachidonoyl-phosphatidate	0,108441332	0,442995376	0,679412483
455,2466151	HMDB0000907, HMDB00907	Sulfolithocholic acid	-0,042480267	0,878481298	0,949238776
457,2351829	HMDB0062312, HMDB0114742, HMDB62312	1-(5Z,8Z,11Z,14Z-eicosatetraenoyl)-sn-glycero-3-phosphate	-0,263595128	0,522399862	0,735917092
457,2594616	HMDB0037611, HMDB37611	Lucidenic acid A	-0,061249002	0,358933595	0,61597972
459,1304197	HMDB0030869, HMDB0040129, HMDB0041385, HMDB0135246, HMDB0135251, HMDB0135253, HMDB0135254, HMDB30869, HMDB40129, HMDB41385	Wistin	0,045193429	0,767211626	0,883342587
459,2508939	HMDB0062313, HMDB0114747, HMDB0114759, HMDB62313	1-(8Z,11Z,14Z-eicosatrienoyl)-glycero-3-phosphate	0,213521508	0,001905772	0,01888447
461,1673968	HMDB0039233, HMDB39233	Verbasoside	0,028766315	0,701026953	0,847843971
461,2176293	HMDB0010337, HMDB10337	6-Dehydrotestosterone glucuronide	0,290671211	0,125667762	0,330067134
462,1724398	HMDB0015542, HMDB15542	Pivampicillin	0,046711541	0,751314885	0,872880287
464,3021023	HMDB0000138, HMDB0000331, HMDB00138, HMDB0032596, HMDB00331, HMDB32596	Cholylglycine	0,344053186	0,420831542	0,660009181
465,1388596	HMDB0036561, HMDB0126687, HMDB0126714, HMDB0127780, HMDB0127781, HMDB0132675, HMDB36561	Agnuside	0,190641214	1,15885E-07	7,21799E-06

m/z	HMDB ID alternatives	Suggested annotation	*Comparison [vegan] - [omnivore]* Fold Change (log2)	p-value	p-value (BH adj.)
465,3037931	HMDB0000653, HMDB00653	Cholesterol sulfate	-0,39641202	2,34835E-05	0,000585075
465,984622	HMDB0000998, HMDB00998	dCTP	-0,057723895	0,050053679	0,179616493
466,1742006	HMDB0031949, HMDB31949	Bis-N-butyl phthalate	0,070918776	0,25746767	0,514590464
466,3069931	HMDB0042042, HMDB42042	Tiropramide	-0,401242601	1,9728E-06	6,59001E-05
467,9448605	HMDB0060640, HMDB60640	Lamivudine-triphosphate	0,077296921	0,749318056	0,872880287
469,1508235	HMDB0129028, HMDB0129029	3,4,5-trihydroxy-6-3-hydroxy-5-[(E)-2-(4-hydroxyphenyl)ethenyl]-2-[(1E)-3-methylbuta-1,3-dien-1-yl]phenoxyoxane-2-carboxylic acid	0,115872838	0,000661246	0,008356613
469,2246795	HMDB0038199, HMDB38199	Lucidenic acid D1	0,390524693	0,062471778	0,204664919
469,258412	HMDB0003218, HMDB0034415, HMDB0036437, HMDB03218, HMDB34415, HMDB36437	Withanolide	0,417569333	0,035438206	0,147857013
471,0949294	HMDB0038363, HMDB0040293, HMDB38363, HMDB40293	(-)-Epigallocatechin 3-(3-methyl-gallate)	-0,170069725	0,293669053	0,554284447
471,2425658	HMDB0002522, HMDB0002586, HMDB0002642, HMDB02522, HMDB02586, HMDB02642	Chenodeoxycholic acid sulfate	0,283508128	0,301508179	0,561857104
471,3464307	HMDB0002392, HMDB0033233, HMDB0034523, HMDB0034530, HMDB0034567, HMDB0035106, HMDB0035107, HMDB0035258, HMDB0035260, HMDB0035748, HMDB0035758, HMDB0035962, HMDB0035977, HMDB0036001, HMDB0036063, HMDB0036064, HMDB0036638, HMDB0036654, HMDB0037779, HMDB0041043, HMDB02392, HMDB33233, HMDB34523, HMDB34530, HMDB34567, HMDB35106, HMDB35107, HMDB35258, HMDB35260, HMDB35748, HMDB35758, HMDB35962, HMDB35977, HMDB36001, HMDB36063, HMDB36064, HMDB36638, HMDB36654, HMDB37779, HMDB41043	Maslinic acid	1,093634283	0,000899013	0,010593775
473,3616716	HMDB0034505, HMDB0034528, HMDB0034644, HMDB0034683, HMDB0035326, HMDB0039692, HMDB34505, HMDB34528, HMDB34644, HMDB34683, HMDB35326, HMDB39692	Soyasapogenol A	1,185193096	0,001796342	0,018214069
474,2847152	HMDB0014907, HMDB14907	Hydrocortamate	0,195267922	0,160100469	0,388879134
477,1044483	HMDB0029479, HMDB0030548, HMDB0030805, HMDB0033650, HMDB0036120, HMDB0037332, HMDB0037360, HMDB0039190, HMDB0039211, HMDB0039338, HMDB0040509, HMDB0040510, HMDB0041743, HMDB0041745, HMDB0126054, HMDB0126055, HMDB0126056, HMDB0126057, HMDB0126140, HMDB0126573, HMDB0126590, HMDB0126591, HMDB0126596, HMDB0126597, HMDB0127510, HMDB0127511, HMDB0127513, HMDB0127514, HMDB0128063, HMDB0128064, HMDB0128065, HMDB0128066, HMDB0128067, HMDB0128068, HMDB0128549, HMDB0128597, HMDB0128598, HMDB0128599, HMDB0129310, HMDB0129320, HMDB0129425, HMDB0129989, HMDB0129990, HMDB0129991, HMDB0130192, HMDB0130193, HMDB0130194, HMDB0130195, HMDB0130196, HMDB0130197, HMDB0133822, HMDB0133842, HMDB0133844, HMDB0137325, HMDB29479, HMDB30548, HMDB30805, HMDB33650, HMDB36120, HMDB37332, HMDB37360, HMDB39190, HMDB39211, HMDB39338, HMDB40509, HMDB40510, HMDB41743, HMDB41745	Isorhamnetin 7-glucoside	0,086610458	0,477138962	0,706392487
477,1941983	HMDB0032771, HMDB0032774, HMDB32771, HMDB32774	Eremopetasitenin C2	0,161970646	0,706750805	0,852402078
478,2919659	HMDB0011475, HMDB0011476, HMDB0011505, HMDB0011506, HMDB11475, HMDB11476, HMDB11505, HMDB11506	LysoPE(0:0/18:1(11Z))	0,237106152	0,482291882	0,709204926
480,3087099	HMDB0010381, HMDB0011129, HMDB0011130, HMDB10381, HMDB11129, HMDB11130	LysoPC(15:0)	0,045537613	0,891540789	0,954337611
481,4170523	HMDB0038961, HMDB38961	N-[2-(1H-Indol-3-yl)ethyl]docosanamide	0,963774869	0,020097829	0,100144611
482,1585119	HMDB0133696, HMDB0133697	2-amino-4-(1-[(carboxymethyl)-C-hydroxycarbonimidoyl]-2-[(2-hydroxy-2-methyl-3-oxo-1-phenylbutyl)sulfanyl]eth C-hydroxycarbonimidoyl)b acid	0,096563168	0,458753896	0,692099303
483,1652572	HMDB0035733, HMDB0125915, HMDB35733	Dukunolide E	-0,055717279	0,519950025	0,73484023
485,3271059	HMDB0034526, HMDB0034689, HMDB0035259, HMDB0035261, HMDB0035295, HMDB0036652, HMDB0036851, HMDB0037963, HMDB0038737, HMDB0040499, HMDB0041041, HMDB34526, HMDB34689, HMDB35259, HMDB35261, HMDB35295, HMDB36652, HMDB36851, HMDB37963, HMDB38737, HMDB40499, HMDB41041	Bassic acid	0,837995399	0,006028491	0,041385986
487,2553143	HMDB0038732, HMDB38732	alpha-Ionol O-[arabinosyl-(1->6)-glucoside]	0,372360311	0,025442437	0,116156048
489,1756319	HMDB0130297	3,4,5-trihydroxy-6-(2-[6-(2-methylbut-3-en-2-yl)-7-oxo-2H,3H,7H-furo[3,2-g]chromen-2-yl]propan-2-yloxy)oxane-2-carboxylic acid	0,326295612	0,145119613	0,368933826

m/z	HMDB ID alternatives	Suggested annotation	Comparison [vegan] - [omnivore] Fold Change (log2)	p-value	p-value (BH adj.)
489,229128	HMDB0030630, HMDB0129655, HMDB0129656, HMDB0129657, HMDB0129658, HMDB0129659, HMDB0129660, HMDB0129661, HMDB0129662, HMDB0129663, HMDB0129664, HMDB0129665, HMDB30630	Rubraflavone C	-0,075749852	0,413297347	0,65407493
489,3569164	HMDB0034525, HMDB0034532, HMDB0037783, HMDB34525, HMDB34532, HMDB37783	Barringtogenol C	1,192811845	0,005160973	0,038133139
493,2068132	HMDB0033412, HMDB0037084, HMDB0038610, HMDB33412, HMDB37084, HMDB38610	Gibberellin A20 13-glucoside	0,076649567	0,686402486	0,840650236
493,2460777	HMDB0033655, HMDB33655	7,11-Bisdeacetylvaltrate 7-(3-methylpentanoate) 11-(3-hydroxy-3-methylbutanoate)	0,185329058	0,558343792	0,757470764
495,3343124	HMDB0061702, HMDB0062541, HMDB61702, HMDB62541	2-Palmitoylglycerophosph	0,718257461	0,012380244	0,070559298
497,3855423	HMDB0072193, HMDB0072251, HMDB72193, HMDB72251	TG(10:0/8:0/8:0)	1,201017608	0,108301651	0,296110649
498,2880279	HMDB0000874, HMDB0000896, HMDB0000951, HMDB00874, HMDB00896, HMDB00951, HMDB04011	Tauroursodeoxycholic acid	0,172807942	0,891955451	0,954337611
499,2905268	HMDB0014554, HMDB14554	Mupirocin	0,465487606	0,060499249	0,202905172
500,2776121	HMDB0011487, HMDB0011488, HMDB0011517, HMDB0011518, HMDB11487, HMDB11488, HMDB11517, HMDB11518	LysoPE(0:0/20:4(5Z,8Z,	0,32188588	0,178279469	0,414559191
501,1448255	HMDB0124742, HMDB0124743, HMDB0124744, HMDB0124745, HMDB0124746, HMDB0124747	6-(4,5-dihydroxy-2-methyl-6-[(3,4,5,6-tetrahydroxyoxan-2-yl)methoxy]oxan-3-yloxy)-3,4,5-trihydroxyoxane-2-carboxylic acid	0,009905962	0,805665281	0,904170045
503,1554915	HMDB0125552, HMDB0125553, HMDB0125554, HMDB0125555, HMDB0125556, HMDB0125558, HMDB0125559, HMDB0125560, HMDB0125561, HMDB0125562, HMDB0125563, HMDB0125564, HMDB0125566, HMDB0125567, HMDB0125568, HMDB0125569, HMDB0125571, HMDB0125572, HMDB0125573, HMDB0125576, HMDB0125577, HMDB0125578, HMDB0129040, HMDB0129041, HMDB0129042, HMDB0129045, HMDB0129046, HMDB0129047, HMDB0129048, HMDB0129049, HMDB0129052, HMDB0129053, HMDB0129054, HMDB0129055, HMDB0129057, HMDB0129058, HMDB0129059, HMDB0129061, HMDB0129062, HMDB0129063, HMDB0129064, HMDB0129066, HMDB0129067, HMDB0129068, HMDB0129073, HMDB0129074, HMDB0129075, HMDB0129080, HMDB0129081, HMDB0129082, HMDB0129571	6-6-[(E)-2-(2,4-dihydroxyphenyl)ethenyl 2,4-dihydroxy-3-(3-methylbut-2-en-1-yl)phenoxy-3,4,5-trihydroxyoxane-2-carboxylic acid	0,079018177	0,906340122	0,961999186
503,2607639	HMDB0127533, HMDB0127534	(1S,16R)-5,7,11-trihydroxy-8,8,12,16-tetramethyl-3-[1-(2-methyl-1,3-thiazol-4-yl)prop-1-en-2-yl]-10-methylidene-17-oxa-4-azabicyclo[14.1.0]heptac 4-en-9-one	0,250791008	0,00195536	0,019158135
504,3086355	HMDB0011483, HMDB0011513, HMDB11483, HMDB11513	LysoPE(0:0/20:2(11Z,14	0,213758246	0,456378754	0,691905798
505,9798017	HMDB0001134, HMDB01134	Phosphoadenosine phosphosulfate	0,100906083	0,372885958	0,627715358
505,9894434	HMDB0000538, HMDB0001440, HMDB00538, HMDB0059593, HMDB01440, HMDB59593	ATP	0,026083855	0,508129697	0,727568302
506,3236281	HMDB0011482, HMDB0011512, HMDB11482, HMDB11512	LysoPE(0:0/20:1(11Z))	0,224959826	0,217694563	0,467560737
507,1875872	HMDB0038710, HMDB38710	(7”R,8”R)-4,7”-Epoxy-3”-methoxy-4”,5,9,9”-lignanetetrol 9”-glucoside	0,060325831	0,965948285	0,984003393
507,2440379	HMDB0060828, HMDB60828	ID14326	0,05191992	0,936957317	0,975690018
508,4715918	HMDB0011773, HMDB11773	C14 Cer	-0,144417164	0,176913553	0,413276794
509,2203151	HMDB0000579, HMDB00579	Deuteroporphyrin IX	0,075011158	0,055465252	0,192692031
510,2528748	HMDB0062801, HMDB62801	Sulfoglycolithocholate(2)	0,549110245	1,15774E-06	5,04775E-05
510,2867846	HMDB0035368, HMDB35368	Cytochalasin Ppho	0,335972138	0,00309939	0,025987192
512,2686959	HMDB0002639, HMDB02639	Sulfolithocholylglycine	0,306962874	0,486785468	0,710703477
513,356898	HMDB0034529, HMDB0034647, HMDB0035510, HMDB34529, HMDB34647, HMDB35510	16-Acetylpriverogenin A	0,894220719	0,008215721	0,052677272
514,2842715	HMDB0000036, HMDB0000889, HMDB0000922, HMDB0000932, HMDB00036, HMDB0011637, HMDB00889, HMDB00922, HMDB00932, HMDB11637	Taurocholic acid	-0,05041993	0,485673449	0,710703477
517,2068451	HMDB0060499, HMDB60499	NPC	0,173385934	0,891047285	0,954337611
517,2491609	HMDB0012231, HMDB0060469, HMDB12231, HMDB60469	Geranylfarnesyl diphosphate	0,237358176	0,045185413	0,168598232
517,3147262	HMDB0034392, HMDB0035304, HMDB0035699, HMDB0039691, HMDB34392, HMDB35304, HMDB35699, HMDB39691	Perulactone	0,461308408	0,008976991	0,055517278
517,388468	HMDB0037781, HMDB37781	Ganoderiol C	1,204086717	0,008942473	0,055517278

m/z	HMDB ID alternatives	Suggested annotation	Comparison [vegan] - [omnivore] Fold Change (log2)	p-value	p-value (BH adj.)
519,187318	HMDB0040636, HMDB40636	(7”S,8”S)-4,7”-Epoxy-3,8”-bilign-7-ene-3”,5-dimethoxy-4”,9,9”-triol 4”-glucoside	0,213163186	0,288934412	0,550110933
521,3503751	HMDB0061701, HMDB61701	2-Oleoylglycerophosphoch	1,030179447	0,005450851	0,03900194
523,1017965	HMDB0034318, HMDB0038507, HMDB34318, HMDB38507	Sennidin C	0,199817416	0,403469674	0,646738154
523,298136	HMDB0003577, HMDB03577	VPGPR Enterostatin	0,32873913	0,143822176	0,367779876
524,2764383	HMDB0011496, HMDB0011526, HMDB11496, HMDB11526	LysoPE(0:0/22:6(4Z,7Z,	-0,150148791	0,57583216	0,762286527
527,2650596	HMDB0033976, HMDB0035797, HMDB0035895, HMDB0037565, HMDB33976, HMDB35797, HMDB35895, HMDB37565	Physapubenolide	0,44752383	0,009255744	0,056838093
527,3367619	HMDB0033079, HMDB0035225, HMDB0035335, HMDB33079, HMDB35225, HMDB35335	Ganoderic acid V	0,648895575	0,009703418	0,058417721
535,3398042	HMDB0115487, HMDB0115613, HMDB0115689, HMDB0115781	PA(8:0/16:0)	0,112329202	0,375697379	0,628197929
537,4878797	HMDB0112106, HMDB0112107, HMDB0112111, HMDB0112113, HMDB0112116, HMDB0112118, HMDB0112119, HMDB0112121, HMDB0112146, HMDB0112147, HMDB0112148, HMDB0112149, HMDB0112150, HMDB0112151, HMDB0112152, HMDB0112153, HMDB0112176, HMDB0112179	FAHFA(16:0/9-O-18:0)	0,523056836	0,195577197	0,436172163
539,4308624	HMDB0071246, HMDB0071529, HMDB0072647, HMDB0096559, HMDB0096560, HMDB0096561, HMDB71246, HMDB71529, HMDB72647, HMDB96559, HMDB96560, HMDB96561	TG(8:0/8:0/a-13:0)[rac]	1,55648398	0,061163967	0,203607508
541,2639797	HMDB0010320, HMDB10320	Cortolone-3-glucuronide	0,373761962	0,005892689	0,041385986
544,3421734	HMDB0010393, HMDB0010394, HMDB10393, HMDB10394	LysoPC(20:3(5Z,8Z,11Z	-0,009602252	0,250967986	0,504249041
545,3482369	HMDB0041547, HMDB41547	Hovenidulcigenin B	0,50332337	0,05461827	0,191146254
547,1475453	HMDB0125967, HMDB0125968, HMDB0125969, HMDB0125970, HMDB0125971, HMDB0125972, HMDB0125973, HMDB0125975, HMDB0125976, HMDB0125977, HMDB0125978, HMDB0125979, HMDB0125980, HMDB0125981, HMDB0125982, HMDB0125983, HMDB0125984, HMDB0125985, HMDB0131899, HMDB0132622, HMDB0132623, HMDB0132624, HMDB0132626, HMDB0132628, HMDB0132629, HMDB0132630, HMDB0132631, HMDB0132632, HMDB0132633, HMDB0140256	6-(2-[4,6-dihydroxy-2-methoxy-3-(3-methylbut-2-en-1-yl)phenyl]-6-hydroxy-3-oxo-2,3-dihydro-1-benzofuran-5-yloxy)-3,4,5-trihydroxyoxane-2-carboxylic acid	0,00506018	0,823334036	0,917884016
550,244849	HMDB0060622, HMDB60622	Endoxifen O-glucuronide	0,30760505	0,27281573	0,537009745
550,389631	HMDB0010390, HMDB10390	LysoPC(20:0)	0,879848863	0,018654668	0,095687475
551,3061451	HMDB0004309, HMDB04309	Triterpenoid	0,402367399	0,185889584	0,421027837
551,3605306	HMDB0036298, HMDB36298	Cyclopassifloic acid E	0,725797073	0,167781788	0,398653187
551,4257115	HMDB0002268, HMDB0030576, HMDB0030607, HMDB0033844, HMDB0035138, HMDB0035139, HMDB0035836, HMDB0036869, HMDB0036910, HMDB0036917, HMDB0036921, HMDB0036926, HMDB0060443, HMDB02268, HMDB30576, HMDB30607, HMDB33844, HMDB35138, HMDB35139, HMDB35836, HMDB36869, HMDB36910, HMDB36917, HMDB36921, HMDB36926, HMDB60443	Alpha-Cryptoxanthin	1,115928285	0,036533338	0,149114436
553,9224193	HMDB0011104, HMDB11104	3-Phosphoadenylylselenate	0,076346601	0,81791266	0,913213622
563,2200969	HMDB0004051, HMDB04051	Aerobactin	0,184187306	0,070787468	0,2204524
563,3697193	HMDB0115489, HMDB0115615, HMDB0115691, HMDB0115698, HMDB0115704, HMDB0115783, HMDB0115794, HMDB0115800, HMDB0115814	PA(8:0/18:0)	0,243504864	0,257090368	0,514590464
567,1137794	HMDB0033006, HMDB0037087, HMDB33006, HMDB37087	Neobignonoside	-0,049882764	0,564680147	0,762230786
567,4616888	HMDB0071165, HMDB0071222, HMDB0071443, HMDB0071663, HMDB0071676, HMDB0071685, HMDB0071738, HMDB0071968, HMDB0072206, HMDB0072245, HMDB0072333, HMDB0072338, HMDB0072529, HMDB0072531, HMDB0072546, HMDB0096562, HMDB0096563, HMDB0096564, HMDB0096649, HMDB0096650, HMDB0096651, HMDB0100884, HMDB0100885, HMDB0100886, HMDB71165, HMDB71222, HMDB71443, HMDB71663, HMDB71676, HMDB71685, HMDB71738, HMDB71968, HMDB72206, HMDB72245, HMDB72333, HMDB72338, HMDB72529, HMDB72531, HMDB72546, HMDB96562, HMDB96563, HMDB96564, HMDB96649, HMDB96650, HMDB96651	TG(10:0/13:0/8:0)	1,3607016	0,026981582	0,122541351
571,2910387	HMDB0035987, HMDB35987	Ganoderic acid H	0,107929441	0,575789226	0,762286527
573,4520132	HMDB0007077, HMDB0007329, HMDB0056004, HMDB07077, HMDB07329, HMDB56004	DG(15:0/18:4(6Z,9Z,12Z	0,950380863	0,017181613	0,090255219
575,4670917	HMDB0007075, HMDB0007076, HMDB0007271, HMDB0007300, HMDB0055996, HMDB0056003, HMDB0056142, HMDB07075, HMDB07076, HMDB07271, HMDB07300, HMDB55996, HMDB56003, HMDB56142	DG(15:0/18:3(6Z,9Z,12Z	1,019478305	0,003414678	0,027317428
577,3698907	HMDB0036911, HMDB36911	Anhydroamarouciaxanth B	0,539951475	0,036294899	0,149114436
579,3030875	HMDB0030459, HMDB30459	Hordatine B	0,282052984	0,203418547	0,446844947
583,2543778	HMDB0000054, HMDB0000488, HMDB00054, HMDB00488	Bilirubin	-0,153721703	0,365269328	0,620886655
584,2035791	HMDB0014614, HMDB14614	Montelukast	-0,186130315	0,39506895	0,641527233
587,3387126	HMDB0036842, HMDB36842	Hydratopyrrhoxanthinol	0,040746307	0,976584506	0,990211267
589,3026258	HMDB0004158, HMDB0004160, HMDB04158, HMDB04160	D-Urobilinogen	0,432794923	0,432235053	0,67185199
591,1340712	HMDB0038847, HMDB0041129, HMDB0041719, HMDB0127121, HMDB0127122, HMDB0127170, HMDB0127171, HMDB0127234, HMDB0127235, HMDB0127275, HMDB0127276, HMDB0127295, HMDB0127296, HMDB38847, HMDB41129, HMDB41719	Apigenin 7-[rhamnosyl-(1->2)-galacturonide]	0,041703787	0,769433652	0,883342587
591,3907568	HMDB0039054, HMDB39054	Tuberoside	0,787369069	0,000392801	0,005615123

m/z	HMDB ID alternatives	Suggested annotation	*Comparison [vegan] - [omnivore]* Fold Change (log2)	p-value	p-value (BH adj.)
593,1446377	HMDB0033297, HMDB33297	Kuwanon Z	0,121914399	0,556921206	0,757470764
595,493904	HMDB0031089, HMDB0071223, HMDB0071277, HMDB0071326, HMDB0071400, HMDB0071439, HMDB0071456, HMDB0071508, HMDB0071616, HMDB0071821, HMDB0071842, HMDB0071845, HMDB0071858, HMDB0071864, HMDB0071893, HMDB0071925, HMDB0071928, HMDB0071972, HMDB0072006, HMDB0072048, HMDB0072121, HMDB0072166, HMDB0072345, HMDB0072420, HMDB0072489, HMDB0072569, HMDB0072572, HMDB0072650, HMDB0072686, HMDB0072725, HMDB0072811, HMDB0094827, HMDB0094828, HMDB0094829, HMDB0094830, HMDB0094831, HMDB0094832, HMDB0095052, HMDB0095053, HMDB0095054, HMDB0095055, HMDB0095056, HMDB0095057, HMDB0096565, HMDB0096566, HMDB0096567, HMDB0096568, HMDB0096569, HMDB0096570, HMDB0096652, HMDB0096653, HMDB0096654, HMDB0096739, HMDB0096740, HMDB0096741, HMDB0096742, HMDB0096743, HMDB0096744, HMDB0100887, HMDB0100888, HMDB0100889, HMDB0100974, HMDB0100975, HMDB0100976, HMDB0105194, HMDB0105195, HMDB0105196, HMDB31089, HMDB71223, HMDB71277, HMDB71326, HMDB71400, HMDB71439, HMDB71456, HMDB71508, HMDB71616, HMDB71821, HMDB71842, HMDB71845, HMDB71858, HMDB71864, HMDB71893, HMDB71925, HMDB71928, HMDB71972, HMDB72006, HMDB72048, HMDB72121, HMDB72166, HMDB72345, HMDB72420, HMDB72489, HMDB72569, HMDB72572, HMDB72650, HMDB72686, HMDB72725, HMDB72811, HMDB94827, HMDB94828, HMDB94829, HMDB94830, HMDB94831, HMDB94832, HMDB95052, HMDB95053, HMDB95054, HMDB95055, HMDB95056, HMDB95057, HMDB96565, HMDB96566, HMDB96567, HMDB96568, HMDB96569, HMDB96570, HMDB96652, HMDB96653, HMDB96654, HMDB96739, HMDB96740, HMDB96741, HMDB96742, HMDB96743, HMDB96744	Glycerol triundecanoate	1,269313034	0,013378706	0,074083072
599,4674612	HMDB0007085, HMDB0007561, HMDB0056006, HMDB07085, HMDB07561, HMDB56006	DG(15:0/20:5(5Z,8Z,11Z	0,617252431	0,057197259	0,196244799
607,1068749	HMDB0037582, HMDB37582	Prunin 6'''-O-gallate	0,053437754	0,543626924	0,754845029
607,1068749	HMDB0036336, HMDB36336	Prodelphinidin A1	0,053437754	0,543626924	0,754845029
611,1244463	HMDB0062695, HMDB62695	CMP-N-acetyl-beta-neuraminate(2-)	0,000237304	0,751758137	0,872880287
611,1244463	HMDB0132733, HMDB0132734, HMDB0132735, HMDB0132736	6-[4-(3-[3,4-dihydroxy-5-(hydroxymethyl)oxolan-2-yl]oxy-5,7-dihydroxy-4-oxo-3,4-dihydro-2H-1-benzopyran-2-yl)-2-hydroxyphenoxy]-3,4,5-trihydroxyoxane-2-carboxylic acid	0,000237304	0,751758137	0,872880287
613,1208228	HMDB0039192, HMDB0039194, HMDB39192, HMDB39194	2-Cinnamoyl-1,6-digalloyl-beta-D-glucopyranose	0,113259275	0,582367069	0,762836399
615,1348441	HMDB0029903, HMDB29903	3,5-Dicaffeoyl-4-succinoylquinic acid	0,198558725	0,398591663	0,643651723
619,1654411	HMDB0029908, HMDB29908	Camellianin A	0,081045515	0,09561594	0,271586644
619,2894036	HMDB0061690, HMDB0062722, HMDB61690, HMDB62722	1-Arachidonoylglyceropho	0,103807635	0,549695925	0,756072946
620,5967051	HMDB0004952, HMDB0011766, HMDB04952, HMDB11766	C22 Cer	-0,6103745	0,000103556	0,001689068
621,4351177	HMDB0039544, HMDB0041429, HMDB39544, HMDB41429	(20R)-Ginsenoside Rh2	0,837412689	0,010619762	0,063085924
632,1733014	HMDB0035420, HMDB35420	Pyranocyanin A	0,177768498	0,781008461	0,889038232
639,1022505	HMDB0127345	2-[5,7-dihydroxy-2-(4-hydroxyphenyl)-4-oxo-4H-chromen-6-yl]-6-methyl-5-oxo-3-[(3,4,5-trihydroxy-6-methyloxan-2-yl)oxy]oxan-4-yloxidanesulfonic acid	0,063623816	0,364846402	0,620886655
640,2928591	HMDB0013058, HMDB0013062, HMDB0013063, HMDB0060154, HMDB13058, HMDB13062, HMDB13063, HMDB60154	S-(9-deoxy-delta9,12-PGD2)-glutathione	0,080412753	0,397219117	0,642625361
642,3057335	HMDB0013056, HMDB0013057, HMDB0013060, HMDB0013061, HMDB0060155, HMDB0062478, HMDB13056, HMDB13057, HMDB13060, HMDB13061, HMDB60155, HMDB62478	S-(11-OH-9-deoxy-delta9,12-PGD2)-glutathione	0,081189689	0,190026305	0,428172966
644,2403999	HMDB0014429, HMDB14429	Acarbose	0,100498774	0,363995566	0,620886655
644,5001425	HMDB0010701, HMDB10701	C18 CerP	-0,366504481	0,00061494	0,007885705
648,6281538	HMDB0000831, HMDB0004956, HMDB0011769, HMDB00831, HMDB04956, HMDB11769	C24 Cer	-0,505086008	2,60457E-05	0,000630884
649,1971706	HMDB0034682, HMDB34682	cis-Ferulic acid [arabinosyl-(1->3)-[glucosyl-(1->6)]-glucosyl] ester	0,076315612	0,512358178	0,730874126

m/z	HMDB ID alternatives	Suggested annotation	*Comparison [vegan] - [omnivore]* Fold Change (log2)	p-value	p-value (BH adj.)
649,21183	HMDB0030627, HMDB0034281, HMDB30627, HMDB34281	5-Hydroxy-7,3”,4”-trimethoxy-8-methylisoflavone 5-neohesperidoside	0,109346091	0,578165149	0,762373662
650,6457845	HMDB0011768, HMDB11768	C24DH Cer	-0,479256009	3,68435E-06	0,000114741
654,6050673	HMDB0035469, HMDB35469	N-(2R-Hydroxydocosanoyl)-2S-amino-1,3S,4R-octadecanetriol	-0,510958925	0,000113938	0,001806429
663,3899173	HMDB0031837, HMDB31837	3-O-trans-Feruloyleuscaphic acid	0,269740945	0,067811904	0,214705473
664,6610724	HMDB0011770, HMDB11770	Cer(d18:0/25:0)	-0,111555471	0,781076747	0,889038232
671,4640511	HMDB0007860, HMDB0114900, HMDB0114925, HMDB0114949, HMDB0115092, HMDB0115504, HMDB0115532, HMDB07860	PA(16:0/18:2(9Z,12Z))	-0,158500761	0,000715007	0,008906941
673,0922488	HMDB0060465, HMDB60465	cyclic GMP-AMP	0,029080945	0,245198342	0,499562978
673,4789745	HMDB0007858, HMDB0007859, HMDB0031130, HMDB0114877, HMDB0114899, HMDB0114924, HMDB0115062, HMDB0115091, HMDB07858, HMDB07859, HMDB31130	PA(16:0/18:1(11Z))	-0,264597594	0,00184486	0,018491012
673,5782828	HMDB0007204, HMDB0007233, HMDB0007261, HMDB0007289, HMDB0007318, HMDB0007371, HMDB0007372, HMDB0007399, HMDB0007427, HMDB0007455, HMDB0007484, HMDB0007597, HMDB0007598, HMDB0007625, HMDB0007652, HMDB0007653, HMDB0056070, HMDB0056076, HMDB0056098, HMDB0056105, HMDB0056188, HMDB0056207, HMDB0056222, HMDB07204, HMDB07233, HMDB07261, HMDB07289, HMDB07318, HMDB07371, HMDB07372, HMDB07399, HMDB07427, HMDB07455, HMDB07484, HMDB07597, HMDB07598, HMDB07625, HMDB07652, HMDB07653, HMDB56070, HMDB56076, HMDB56098, HMDB56105, HMDB56188, HMDB56207, HMDB56222	DG(18:1(11Z)/22:2(13Z,	-0,891403656	3,48767E-09	5,06875E-07
673,5951703	HMDB0006736, HMDB0010373, HMDB06736, HMDB10373	CE(20:3(8Z,11Z,14Z))	-0,947786384	1,48133E-09	2,9748E-07
675,1099948	HMDB0001312, HMDB01312	Diadenosine diphosphate	0,042311466	0,880357802	0,950089113
679,2826539	HMDB0038449, HMDB38449	Vomifoliol 9-[glucosyl-(1->4)-xylosyl-(1->6)-glucoside]	0,162038381	0,420392715	0,660009181
687,4019902	HMDB0115038	PA(18:4(6Z,9Z,12Z,15Z	0,037128217	0,001040847	0,011787251
690,5055952	HMDB0007868, HMDB0007932, HMDB0008826, HMDB0008923, HMDB0008986, HMDB0029174, HMDB0112964, HMDB0113041, HMDB0113841, HMDB0113845, HMDB0113940, HMDB05317, HMDB05325, HMDB07868, HMDB07932, HMDB08826, HMDB08923, HMDB08986, HMDB29174	PC(14:0/15:0)	-0,083663305	0,298976029	0,559457291
691,432188	HMDB0031093, HMDB0114792, HMDB0114807, HMDB0114808, HMDB0114863, HMDB0114957, HMDB0114982, HMDB0114983, HMDB0115009, HMDB0115010, HMDB0115035, HMDB0115202, HMDB0115350, HMDB0115376, HMDB0115401, HMDB31093	9,12,15-Octadecatrienoic acid 1-[(phosphonoxy)methyl]-1,2-ethanediyl ester	0,144825121	0,43918136	0,677816187
692,2001742	HMDB0031996, HMDB31996	Licorice glycoside E	0,081572154	0,629532186	0,797895444
693,1106213	HMDB0033274, HMDB33274	Guavin B	0,069046263	0,546112454	0,756072946
695,4651113	HMDB0031092, HMDB0114790, HMDB0114841, HMDB0114860, HMDB0114880, HMDB0114903, HMDB0114904, HMDB0114928, HMDB0114929, HMDB0114954, HMDB0114979, HMDB0114980, HMDB0115006, HMDB0115007, HMDB0115032, HMDB0115122, HMDB0115147, HMDB0115174, HMDB0115323, HMDB0115513, HMDB0115565, HMDB0116701, HMDB31092	1,2-Di-(9Z,12Z-octadecadienoyl)-sn-glycero-3-phosphate	0,025520513	0,107320058	0,295428876
704,5226728	HMDB0007869, HMDB0007934, HMDB0007965, HMDB0008892, HMDB0008988, HMDB0010567, HMDB0112959, HMDB0113084, HMDB0113865, HMDB0113942, HMDB07869, HMDB07934, HMDB07965, HMDB08892, HMDB08988, HMDB10567	PC(14:0/16:0)	-0,095090384	0,917739393	0,96534228
714,5086806	HMDB0008835, HMDB0008867, HMDB0008928, HMDB0008959, HMDB0008960, HMDB0009023, HMDB0009056, HMDB0009088, HMDB0009251, HMDB0009283, HMDB0113020, HMDB0113166, HMDB0113847, HMDB0113848, HMDB0113849, HMDB0113879, HMDB0114012, HMDB0114039, HMDB0114065, HMDB05322, HMDB08835, HMDB08867, HMDB08928, HMDB08959, HMDB08960, HMDB09023, HMDB09056, HMDB09088, HMDB09251, HMDB09283	PE(14:0/20:2(11Z,14Z))	-0,454527607	0,002105242	0,019693506
716,5234136	HMDB0007936, HMDB0008000, HMDB0008834, HMDB0008866, HMDB0008926, HMDB0008927, HMDB0008958, HMDB0008990, HMDB0009022, HMDB0009055, HMDB0009218, HMDB0009250, HMDB0112953, HMDB0112954, HMDB0113112, HMDB0113139, HMDB0113850, HMDB0113851, HMDB0113852, HMDB0113853, HMDB0113965, HMDB0113986, HMDB0114011, HMDB0114038, HMDB05320, HMDB05344, HMDB07936, HMDB08000, HMDB08834, HMDB08866, HMDB08926, HMDB08927, HMDB08958, HMDB08990, HMDB09022, HMDB09055, HMDB09218, HMDB09250	PC(15:0/16:1(9Z))	-0,355601565	0,011194729	0,06507869
719,4641199	HMDB0114849, HMDB0114868, HMDB0114869, HMDB0114911, HMDB0114936, HMDB0114961, HMDB0114962, HMDB0114987, HMDB0115014, HMDB0115127, HMDB0115128, HMDB0115152, HMDB0115179, HMDB0115203, HMDB0115204, HMDB0115353, HMDB0115379, HMDB0115404, HMDB0115523, HMDB0115525, HMDB0115526, HMDB0115542, HMDB0115569, HMDB0115570	PA(16:0/22:6(4Z,7Z,10Z	-0,130510996	0,011169757	0,06507869
719,4882277	HMDB0010571, HMDB0010585, HMDB10571, HMDB10585	PG(16:0/16:1(9Z))	-0,203882471	0,036140432	0,149114436
720,2516602	HMDB0032859, HMDB32859	Aquifoliunine EII	-0,04259577	0,698072768	0,847798683

m/z	HMDB ID alternatives	Suggested annotation	Comparison [vegan] - [omnivore] Fold Change (log2)	p-value	p-value (BH adj.)
721,4818174	HMDB0114847, HMDB0114848, HMDB0114867, HMDB0114886, HMDB0114909, HMDB0114910, HMDB0114934, HMDB0114935, HMDB0114960, HMDB0115040, HMDB0115102, HMDB0115126, HMDB0115150, HMDB0115151, HMDB0115177, HMDB0115178, HMDB0115327, HMDB0115352, HMDB0115378, HMDB0115495, HMDB0115521, HMDB0115522, HMDB0115524, HMDB0115540, HMDB0115541, HMDB0116704	PA(16:0/22:5(4Z,7Z,10Z	-0,430189127	0,000266174	0,003933957
722,5116733	HMDB0009149, HMDB0009150, HMDB0009182, HMDB0009183, HMDB0009214, HMDB0009411, HMDB0009444, HMDB0011352, HMDB0011353, HMDB0011379, HMDB0011410, HMDB0011411, HMDB0011443, HMDB0011444, HMDB09149, HMDB09150, HMDB09182, HMDB09183, HMDB09214, HMDB09411, HMDB09444, HMDB11352, HMDB11353, HMDB11379, HMDB11410, HMDB11411, HMDB11443, HMDB11444	PE(18:3(6Z,9Z,12Z)/P-18:1(11Z))	-1,106099228	2,73582E-08	2,38564E-06
723,4241648	HMDB0039970, HMDB39970	Bis(4-methoxybenzoyl)-3a,29-dihydroxy-8-multifloren-7-one	-0,176332333	0,000604151	0,007862977
726,5418527	HMDB0009050, HMDB0009051, HMDB0009083, HMDB0009084, HMDB0009115, HMDB0009312, HMDB0011349, HMDB0011376, HMDB0011407, HMDB0011408, HMDB0011440, HMDB0011441, HMDB09050, HMDB09051, HMDB09083, HMDB09084, HMDB09115, HMDB09312, HMDB11349, HMDB11376, HMDB11407, HMDB11408, HMDB11440, HMDB11441	PE(18:1(11Z)/P-18:1(11Z))	-0,76263493	5,47256E-08	4,33825E-06
730,5475699	HMDB0033621, HMDB33621	Araliacerebroside	-0,375012917	0,048055545	0,174912962
731,5582902	HMDB0114793, HMDB0114843, HMDB0114881, HMDB0115054, HMDB0115067, HMDB0115230, HMDB0115243, HMDB0115428, HMDB0115640, HMDB0115648, HMDB0115656, HMDB0115672, HMDB0115702, HMDB0115721, HMDB0115730, HMDB0115738, HMDB0115747, HMDB0115758, HMDB0115764, HMDB0115798, HMDB0115825, HMDB0115837, HMDB0115854, HMDB0115866, HMDB0115875, HMDB0115883, HMDB0115895, HMDB0115912, HMDB0115923, HMDB0115940	PA(14:0/24:0)	-0,383717019	0,001468628	0,0156176
736,5279201	HMDB0008258, HMDB0011214, HMDB08258, HMDB11214	PC(18:4(6Z,9Z,12Z,15Z 16:0)	-0,635839537	2,22492E-07	1,21258E-05
742,5381866	HMDB0007940, HMDB0008132, HMDB0008843, HMDB0008875, HMDB0008934, HMDB0008966, HMDB0008994, HMDB0009025, HMDB0009026, HMDB0009058, HMDB0009059, HMDB0009090, HMDB0009254, HMDB0009286, HMDB0009515, HMDB0009547, HMDB0113025, HMDB0113339, HMDB0113856, HMDB0113857, HMDB0113858, HMDB0113884, HMDB0113943, HMDB0114015, HMDB0114042, HMDB0114068, HMDB0114211, HMDB0114239, HMDB05332, HMDB05343, HMDB05347, HMDB07940, HMDB08132, HMDB08843, HMDB08875, HMDB08934, HMDB08966, HMDB08994, HMDB09025, HMDB09026, HMDB09058, HMDB09059, HMDB09090, HMDB09254, HMDB09286, HMDB09515, HMDB09547	PC(15:0/18:2(9Z,12Z))	-0,205400586	0,023621217	0,112555746
746,5109073	HMDB0005780, HMDB0009479, HMDB0009480, HMDB0009708, HMDB0011420, HMDB0011453, HMDB05780, HMDB09479, HMDB09480, HMDB09708, HMDB11361, HMDB11420, HMDB11453	PE(O-16:1(1Z)/22:6(4Z,7Z,10Z	-0,999468033	1,55453E-06	6,06903E-05
752,4525639	HMDB0012350, HMDB0012430, HMDB0112299, HMDB0112312, HMDB0112359, HMDB0112489, HMDB0112655, HMDB0112679, HMDB12350, HMDB12430	PS(14:1(9Z)/20:4(5Z,8Z,	-0,316476544	0,000580107	0,007782359
754,4651928	HMDB0012339, HMDB0012349, HMDB0012370, HMDB0012410, HMDB0012419, HMDB0012429, HMDB0112298, HMDB0112311, HMDB0112344, HMDB0112358, HMDB0112447, HMDB0112488, HMDB0112594, HMDB0112654, HMDB12339, HMDB12349, HMDB12370, HMDB12410, HMDB12419, HMDB12429	PS(14:0/20:4(5Z,8Z,11Z	-0,051622128	0,181767944	0,417740595
756,5539501	HMDB0007880, HMDB0007912, HMDB0007973, HMDB0008004, HMDB0008005, HMDB0008068, HMDB0008101, HMDB0008133, HMDB0008296, HMDB0008328, HMDB0008909, HMDB0009549, HMDB0010565, HMDB0112966, HMDB0112967, HMDB0112968, HMDB0112969, HMDB0112992, HMDB0113010, HMDB0113047, HMDB0113089, HMDB0113169, HMDB0113312, HMDB0113340, HMDB0113536, HMDB0113563, HMDB0113925, HMDB0114241, HMDB07880, HMDB07912, HMDB07973, HMDB08004, HMDB08005, HMDB08068, HMDB08101, HMDB08133, HMDB08296, HMDB08328, HMDB08909, HMDB09549, HMDB10565	PC(14:0/20:2(11Z,14Z))	-0,268983483	7,52559E-05	0,001396237
762,5065213	HMDB0008946, HMDB0008977, HMDB0008978, HMDB0009038, HMDB0009071, HMDB0009102, HMDB0009103, HMDB0009133, HMDB0009134, HMDB0009166, HMDB0009167, HMDB0009198, HMDB0009294, HMDB0009325, HMDB0009326, HMDB0009358, HMDB0009359, HMDB0009390, HMDB0009423, HMDB0009454, HMDB0009455, HMDB0009617, HMDB0009650, HMDB0009682, HMDB0113036, HMDB0113675, HMDB0113896, HMDB0113913, HMDB0113914, HMDB0113975, HMDB0114076, HMDB0114103, HMDB0114104, HMDB0114132, HMDB0114133, HMDB0114160, HMDB0114385, HMDB0114524, HMDB0114550, HMDB0114576, HMDB05324, HMDB08946, HMDB08977, HMDB08978, HMDB09038, HMDB09071, HMDB09102, HMDB09103, HMDB09133, HMDB09134, HMDB09166, HMDB09167, HMDB09198, HMDB09294, HMDB09325, HMDB09326, HMDB09358, HMDB09359, HMDB09390, HMDB09423, HMDB09454, HMDB09455, HMDB09617, HMDB09650, HMDB09682	PE(16:0/22:6(4Z,7Z,10Z	-0,670417194	9,63069E-05	0,001646659
763,7183397	HMDB0007615, HMDB0007811, HMDB0042090, HMDB0042931, HMDB0056088, HMDB0094403, HMDB0094404, HMDB0094405, HMDB0094406, HMDB0094407, HMDB0094408, HMDB0094514, HMDB0094515, HMDB0094516, HMDB0094517, HMDB0094574, HMDB0094575, HMDB0094576, HMDB0094577, HMDB0094628, HMDB0094629, HMDB0094630, HMDB0094631, HMDB0094632, HMDB0094633, HMDB07615, HMDB07811, HMDB42090, HMDB42931, HMDB56088, HMDB94403, HMDB94404, HMDB94405, HMDB94406, HMDB94407, HMDB94408, HMDB94514, HMDB94515, HMDB94516, HMDB94517, HMDB94574, HMDB94575, HMDB94576, HMDB94577, HMDB94628, HMDB94629, HMDB94630, HMDB94631, HMDB94632, HMDB94633	DG(22:0/24:0/0:0)	-0,08943238	0,124521031	0,32804332

			Comparison [vegan] - [omnivore]		
**m/z**	**HMDB ID alternatives**	**Suggested annotation**	**Fold Change (log2)**	**p-value**	**p-value (BH adj.)**
766,5369853	HMDB0007949, HMDB0007950, HMDB0008428, HMDB0008461, HMDB0008943, HMDB0008969, HMDB0009003, HMDB0009004, HMDB0009034, HMDB0009035, HMDB0009067, HMDB0009068, HMDB0009099, HMDB0009131, HMDB0009164, HMDB0009196, HMDB0009228, HMDB0009259, HMDB0009260, HMDB0009291, HMDB0009322, HMDB0009323, HMDB0009355, HMDB0009356, HMDB0009387, HMDB0009420, HMDB0009583, HMDB0113034, HMDB0113594, HMDB0113893, HMDB0113951, HMDB0113971, HMDB0113972, HMDB0113993, HMDB0114018, HMDB0114019, HMDB0114045, HMDB0114046, HMDB0114073, HMDB0114100, HMDB0114101, HMDB0114129, HMDB0114130, HMDB0114157, HMDB0114272, HMDB0114300, HMDB0114327, HMDB0114355, HMDB0114495, HMDB0116711, HMDB05333, HMDB07949, HMDB07950, HMDB08428, HMDB08461, HMDB08943, HMDB08969, HMDB09003, HMDB09004, HMDB09034, HMDB09035, HMDB09067, HMDB09068, HMDB09099, HMDB09131, HMDB09164, HMDB09196, HMDB09228, HMDB09259, HMDB09260, HMDB09291, HMDB09322, HMDB09323, HMDB09355, HMDB09356, HMDB09387, HMDB09420, HMDB09583	PC(15:0/20:4(5Z,8Z,11Z	-0,228547968	0,014930805	0,080368283
767,5589472	HMDB0115591	PA(22:1(13Z)/19:2(10Z,	-0,385534131	0,000891811	0,010593775
772,529517	HMDB0009710, HMDB0009711, HMDB0011427, HMDB0011460, HMDB09710, HMDB09711, HMDB11427, HMDB11460	PE(22:6(4Z,7Z,10Z,13Z, 18:1(11Z))	-0,50872065	8,12111E-05	0,001416321
774,5418828	HMDB0009644, HMDB0009645, HMDB0009677, HMDB0009678, HMDB0009709, HMDB0011394, HMDB0011425, HMDB0011426, HMDB0011458, HMDB0011459, HMDB09644, HMDB09645, HMDB09677, HMDB09678, HMDB09709, HMDB11394, HMDB11425, HMDB11426, HMDB11458, HMDB11459	PE(22:5(4Z,7Z,10Z,13Z, 18:1(11Z))	-1,02157926	5,12862E-09	6,38879E-07
774,5418828	HMDB0009314, HMDB0009315, HMDB0009346, HMDB0009379, HMDB0011383, HMDB0011384, HMDB0011415, HMDB0011448, HMDB09314, HMDB09315, HMDB09346, HMDB09379, HMDB11383, HMDB11384, HMDB11415, HMDB11448	PE(20:2(11Z,14Z)/P-18:1(11Z))	-1,02157926	5,12862E-09	6,38879E-07
776,5584948	HMDB0009611, HMDB0009612, HMDB0009643, HMDB0009676, HMDB0011392, HMDB0011393, HMDB0011424, HMDB0011457, HMDB09611, HMDB09612, HMDB09643, HMDB09676, HMDB11392, HMDB11393, HMDB11424, HMDB11457	PE(22:4(7Z,10Z,13Z,16Z 18:1(11Z))	-0,319783008	0,000498741	0,006903214
777,4072859	HMDB0039591, HMDB39591	Periandrin V	-0,107888635	0,001728617	0,017733576
780,4813522	HMDB0012372, HMDB0012403, HMDB0012413, HMDB0012432, HMDB0061503, HMDB0061506, HMDB0061519, HMDB0061546, HMDB0112304, HMDB0112305, HMDB0112316, HMDB0112348, HMDB0112362, HMDB0112427, HMDB0112451, HMDB0112491, HMDB0112492, HMDB0112658, HMDB0112682, HMDB0112789, HMDB0112815, HMDB0112839, HMDB0113816, HMDB0116751, HMDB0116752, HMDB12372, HMDB12403, HMDB12413, HMDB12432, HMDB61503, HMDB61506, HMDB61519, HMDB61546	PS(16:1(9Z)/20:4(5Z,8Z	-0,341513821	3,99607E-05	0,00082966
781,5732329	HMDB0114972, HMDB0114998, HMDB0115025, HMDB0115111, HMDB0115136, HMDB0115255, HMDB0115309, HMDB0115437, HMDB0115438, HMDB0115463, HMDB0115552, HMDB0115578, HMDB0115590, HMDB0115592	PA(18:2(9Z,12Z)/24:1(1	-0,338173464	2,89995E-05	0,000665462
782,4963086	HMDB0012361, HMDB0012371, HMDB0012392, HMDB0012402, HMDB0012412, HMDB0012421, HMDB0012431, HMDB0112303, HMDB0112361, HMDB0112374, HMDB0112390, HMDB0112391, HMDB0112410, HMDB0112449, HMDB0112450, HMDB0112468, HMDB0112490, HMDB0112597, HMDB0112657, HMDB0112788, HMDB0116750, HMDB12361, HMDB12371, HMDB12392, HMDB12402, HMDB12412, HMDB12421, HMDB12431	PS(16:0/20:4(5Z,8Z,11Z	-0,316341345	7,85033E-05	0,001416321
784,5856493	HMDB0000593, HMDB0007888, HMDB0007920, HMDB0007979, HMDB0008011, HMDB0008039, HMDB0008070, HMDB0008071, HMDB0008103, HMDB0008135, HMDB0008299, HMDB0008331, HMDB0008559, HMDB0008590, HMDB00593, HMDB0062690, HMDB0112977, HMDB0113054, HMDB0113076, HMDB0113095, HMDB0113121, HMDB0113148, HMDB0113176, HMDB0113287, HMDB0113314, HMDB0113315, HMDB0113342, HMDB0113539, HMDB0113566, HMDB0113729, HMDB0113933, HMDB0114468, HMDB07888, HMDB07920, HMDB07979, HMDB08011, HMDB08039, HMDB08070, HMDB08071, HMDB08103, HMDB08104, HMDB08135, HMDB08299, HMDB08331, HMDB08559, HMDB08590, HMDB62690	PC(18:1(9Z)/18:1(9Z))	-0,201296174	3,89621E-05	0,00082966
787,6223269	HMDB0114894, HMDB0115082, HMDB0115234, HMDB0115253, HMDB0115433, HMDB0115644, HMDB0115649, HMDB0115660, HMDB0115676, HMDB0115725, HMDB0115731, HMDB0115742, HMDB0115751, HMDB0115760, HMDB0115768, HMDB0115841, HMDB0115858, HMDB0115870, HMDB0115876, HMDB0115887, HMDB0115899, HMDB0115916, HMDB0115927, HMDB0115944	PA(18:0/24:0)	-0,152655367	5,52577E-06	0,000160616
800,678473	HMDB0011695, HMDB11695	SM(d17:1/24:0)	-0,101201336	0,007148601	0,047584578
803,7653822	HMDB0036744, HMDB36744	alpha-Amyrin cerotate	-0,04256969	0,002040038	0,019693506
805,4873937	HMDB0009780, HMDB0009798, HMDB0009802, HMDB09780, HMDB09798, HMDB09802	PI(16:0/16:2(9Z,12Z))	-0,200323771	0,000134495	0,002094283
807,5019	HMDB0009779, HMDB0009797, HMDB09779, HMDB09797	PI(16:0/16:1(9Z))	-0,560795854	9,58522E-06	0,000245833
809,467985	HMDB0038350, HMDB38350	Goyaglycoside g	-0,348809899	2,0602E-07	1,19766E-05
809,6055961	HMDB0115142, HMDB0115289, HMDB0115315, HMDB0115442, HMDB0115559, HMDB0115585, HMDB0115603, HMDB0115604	PA(20:3(5Z,8Z,11Z)/24:	-0,475852283	2,20979E-08	2,14104E-06
810,6828145	HMDB0000140, HMDB0004978, HMDB00140, HMDB04978	Glucosylceramide	-0,222247839	0,002110705	0,019693506
811,6464374	HMDB0032811, HMDB0116409, HMDB32811	Isofucosterol 3-O-[6-O-Hexadecanoyl-b-D-glucopyranoside]	-0,48022359	4,78939E-10	2,08818E-07
819,7835224	HMDB0042180, HMDB0042934, HMDB0043912, HMDB0044641, HMDB0044694, HMDB0094640, HMDB0094641, HMDB42180, HMDB42934, HMDB43912, HMDB44641, HMDB44694, HMDB94640, HMDB94641	TG(14:0/18:0/o-18:0)	-0,098442881	0,00315309	0,026185666
825,466441	HMDB0013477, HMDB0013490, HMDB0013491, HMDB0013518, HMDB0013533, HMDB0013547, HMDB13477, HMDB13490, HMDB13491, HMDB13518, HMDB13533, HMDB13547	PGP(16:0/18:2(9Z,12Z))	-0,175792041	0,00320233	0,026343694
826,4593149	HMDB0015418, HMDB15418	Josamycin	-0,111001637	0,053899962	0,190286507
833,7557909	HMDB0031940, HMDB31940	Arachisprenol 12	-0,090450287	0,001012007	0,011611452

m/z	HMDB ID alternatives	Suggested annotation	*Comparison [vegan] - [omnivore]* Fold Change (log2)	p-value	p-value (BH adj.)
839,6775449	HMDB0032812, HMDB0034089, HMDB32812, HMDB34089	Isofucosterol 3-O-[6-O-Octadecanoyl-b-D-glucopyranoside]	-0,404563458	6,35559E-06	0,00017319
842,2111694	HMDB0125095	5-[(6-[(2-carboxyacetyl)oxy]methy 3,4,5-trihydroxyoxan-2-yl)oxy]-3-[4,5-dihydroxy-6-(hydroxymethyl)-3-[3,4,5-trihydroxy-6-(hydroxymethyl)oxan-2-yl]oxyoxan-2-yl]oxy-7-hydroxy-2-(4-hydroxyphenyl)-1??-chromen-1-ylium	0,005897955	0,249542997	0,503707162
850,1278871	HMDB0001484, HMDB01484, HMDB11665	Acetoacetyl-CoA	0,040131104	0,448840341	0,683051968
850,4864794	HMDB0061557, HMDB0061560, HMDB0061585, HMDB0061593, HMDB0061596, HMDB0061597, HMDB0061601, HMDB0061609, HMDB0061624, HMDB0061628, HMDB61557, HMDB61560, HMDB61585, HMDB61593, HMDB61596, HMDB61597, HMDB61601, HMDB61609, HMDB61624, HMDB61628	PS(DiMe(11,3)/MonoMe	-0,162775997	0,132666229	0,345328215
856,680629	HMDB0009245, HMDB0009277, HMDB0009502, HMDB0009534, HMDB0009725, HMDB0009757, HMDB0114010, HMDB0114036, HMDB0114063, HMDB0114202, HMDB0114230, HMDB0114423, HMDB0114449, HMDB0114610, HMDB0114611, HMDB0114636, HMDB09245, HMDB09277, HMDB09502, HMDB09534, HMDB09725, HMDB09757	PE(20:0/24:1(15Z))	-0,066762016	0,00262028	0,02284884
858,52977	HMDB0112617, HMDB0112618, HMDB0112633, HMDB0112649, HMDB0112674, HMDB0112805, HMDB0112806, HMDB0112829, HMDB0112853, HMDB0112870, HMDB0116765, HMDB0116769, HMDB0116784, HMDB0116789	PS(20:3(5Z,8Z,11Z)/22::	-0,227162715	0,008211368	0,052677272
867,4716155	HMDB0062485, HMDB62485	Lc3Cer	-0,248871216	0,000746773	0,009171629
867,7432441	HMDB0043038, HMDB0043065, HMDB0043071, HMDB0043099, HMDB0043232, HMDB0043238, HMDB0043261, HMDB0043267, HMDB0043294, HMDB0043301, HMDB0043316, HMDB0043317, HMDB0043411, HMDB0043434, HMDB0043467, HMDB0043490, HMDB0043491, HMDB0043513, HMDB0043570, HMDB0043637, HMDB0043659, HMDB0043687, HMDB0043876, HMDB0044711, HMDB0044717, HMDB0045523, HMDB0049096, HMDB0049102, HMDB0049723, HMDB0049729, HMDB0050324, HMDB0050331, HMDB0052413, HMDB43038, HMDB43065, HMDB43071, HMDB43099, HMDB43232, HMDB43238, HMDB43261, HMDB43267, HMDB43294, HMDB43301, HMDB43316, HMDB43317, HMDB43411, HMDB43434, HMDB43467, HMDB43490, HMDB43491, HMDB43513, HMDB43570, HMDB43637, HMDB43659, HMDB43687, HMDB43876, HMDB44711, HMDB44717, HMDB45523, HMDB49096, HMDB49102, HMDB49723, HMDB49729, HMDB50324, HMDB50331, HMDB52413	TG(15:0/16:0/22:4(7Z,1(	-0,204692558	0,000104598	0,001689068
870,6972781	HMDB0008059, HMDB0008091, HMDB0008124, HMDB0008283, HMDB0008315, HMDB0008538, HMDB0008569, HMDB0008761, HMDB0008762, HMDB0008793, HMDB0113307, HMDB0113335, HMDB0113529, HMDB0113555, HMDB0113714, HMDB0113740, HMDB08059, HMDB08091, HMDB08124, HMDB08283, HMDB08315, HMDB08538, HMDB08569, HMDB08761, HMDB08762, HMDB08793	PC(18:0/24:1(15Z))	-0,053107133	0,071424315	0,221644138
874,6559343	HMDB0112384, HMDB0112531, HMDB0112719, HMDB0112886	PS(18:0/24:0)	-0,084968714	0,200596831	0,445090169
878,517482	HMDB0061558, HMDB0061566, HMDB0061569, HMDB0061586, HMDB0061594, HMDB0061598, HMDB0061606, HMDB0061610, HMDB0061618, HMDB0061625, HMDB61558, HMDB61566, HMDB61569, HMDB61586, HMDB61594, HMDB61598, HMDB61606, HMDB61610, HMDB61618, HMDB61625	PS(DiMe(11,3)/MonoMe	-0,068574356	0,330784125	0,594134913
882,4287174	HMDB0116070, HMDB0116086, HMDB0116153, HMDB0116160, HMDB0116177, HMDB0116188, HMDB0116194, HMDB0116211	CDP-DG(a-13:0/i-14:0)	-0,075336076	0,334541562	0,594134913
896,6390584	HMDB0112592, HMDB0112619, HMDB0112634, HMDB0112756, HMDB0112780, HMDB0112896, HMDB0112897, HMDB0112923	PS(20:2(11Z,14Z)/24:1(	-0,032237127	0,225440185	0,473219052
906,5484507	HMDB0061559, HMDB0061567, HMDB0061575, HMDB0061578, HMDB0061595, HMDB0061599, HMDB0061607, HMDB0061615, HMDB0061619, HMDB0061626, HMDB61559, HMDB61567, HMDB61575, HMDB61578, HMDB61595, HMDB61599, HMDB61607, HMDB61615, HMDB61619, HMDB61626	PS(DiMe(11,3)/MonoMe	-0,022252855	0,569936857	0,762286527
908,7092395	HMDB0114494, HMDB0114653	PE-NMe2(22:2(13Z,16Z)/24	-0,034443632	0,024355889	0,114801811
909,4494612	HMDB0034817, HMDB34817	Betavulgaroside X	-0,130511936	0,138745521	0,357947026
909,5497733	HMDB0009821, HMDB0009861, HMDB0009865, HMDB0009884, HMDB0009885, HMDB0009891, HMDB0009892, HMDB0009910, HMDB0009911, HMDB0009920, HMDB09821, HMDB09861, HMDB09865, HMDB09884, HMDB09885, HMDB09891, HMDB09892, HMDB09910, HMDB09911, HMDB09920	PI(18:0/22:6(4Z,7Z,10Z,	-0,428933229	0,000260914	0,003922713
913,7647031	HMDB0053250, HMDB0053263, HMDB0054308, HMDB0054338, HMDB0055108, HMDB0055143, HMDB0055350, HMDB0055356, HMDB0055494, HMDB0055524, HMDB0055534, HMDB0055670, HMDB0055682, HMDB0055764, HMDB0055788, HMDB0055878, HMDB0055926, HMDB0055940, HMDB53250, HMDB53263, HMDB54308, HMDB54338, HMDB55108, HMDB55143, HMDB55350, HMDB55356, HMDB55494, HMDB55524, HMDB55534, HMDB55670, HMDB55682, HMDB55764, HMDB55788, HMDB55878, HMDB55926, HMDB55940	TG(18:3(6Z,9Z,12Z)/22: 18:0)	-0,029757265	0,662659823	0,821962114
915,4601295	HMDB0031139, HMDB0033353, HMDB0039007, HMDB31139, HMDB33353, HMDB39007	Chinenoside V	-0,098427449	0,207848665	0,453738986

m/z	HMDB ID alternatives	Suggested annotation	Comparison [vegan] - [omnivore] Fold Change (log2)	p-value	p-value (BH adj.)
925,8247105	HMDB0043116, HMDB0043122, HMDB0043149, HMDB0043156, HMDB0043298, HMDB0043312, HMDB0043353, HMDB0043380, HMDB0043408, HMDB0043429, HMDB0043465, HMDB0043486, HMDB0043550, HMDB0043632, HMDB0046288, HMDB0046294, HMDB0047039, HMDB0047046, HMDB0050328, HMDB0051429, HMDB0051934, HMDB43116, HMDB43122, HMDB43149, HMDB43156, HMDB43298, HMDB43312, HMDB43353, HMDB43380, HMDB43408, HMDB43429, HMDB43465, HMDB43486, HMDB43550, HMDB43632, HMDB46288, HMDB46294, HMDB47039, HMDB47046, HMDB50328, HMDB51429, HMDB51934	TG(15:0/22:0/20:3(5Z,8Z	-0,082513179	0,020518914	0,101087532
931,9081625	HMDB0044024, HMDB0044645, HMDB0044829, HMDB0045453, HMDB0045606, HMDB0046231, HMDB0046355, HMDB0047076, HMDB44024, HMDB44645, HMDB44829, HMDB45453, HMDB45606, HMDB46231, HMDB46355, HMDB47076	TG(16:0/24:0/o-18:0)	-0,224627365	3,97934E-05	0,00082966
933,4677023	HMDB0031364, HMDB31364	Ampeloside Bs1	-0,051276437	0,73128965	0,866419259
934,4591869	HMDB0116045, HMDB0116047, HMDB0116053, HMDB0116169	CDP-DG(a-13:0/18:2(9Z,11Z))	0,024712933	0,294619013	0,554876413
937,8254029	HMDB0005401, HMDB0005470, HMDB0042232, HMDB0042260, HMDB0042266, HMDB0042435, HMDB0042493, HMDB0042499, HMDB0042615, HMDB0042637, HMDB0042681, HMDB0042696, HMDB0042817, HMDB0043960, HMDB0043986, HMDB0043992, HMDB0044019, HMDB0044176, HMDB0044203, HMDB0044209, HMDB0044235, HMDB0044242, HMDB0044289, HMDB0044323, HMDB0044344, HMDB0044364, HMDB0044404, HMDB0044419, HMDB0044485, HMDB0044505, HMDB0044532, HMDB0044767, HMDB0044798, HMDB0044824, HMDB0044974, HMDB0044980, HMDB0045000, HMDB0045032, HMDB0045039, HMDB0045090, HMDB0045110, HMDB0045141, HMDB0045162, HMDB0045182, HMDB0045220, HMDB0045235, HMDB0045299, HMDB0045318, HMDB0045344, HMDB0045546, HMDB0045570, HMDB0045576, HMDB0045601, HMDB0045719, HMDB0045725, HMDB0045745, HMDB0045751, HMDB0045775, HMDB0045782, HMDB0045794, HMDB0045795, HMDB0045880, HMDB0045900, HMDB0045930, HMDB0045950, HMDB0045951, HMDB0046082, HMDB0046101, HMDB0046272, HMDB0046320, HMDB0046326, HMDB0046350, HMDB0046463, HMDB0046469, HMDB0046492, HMDB0046499, HMDB0046517, HMDB0046524, HMDB0046559, HMDB0046641, HMDB0046660, HMDB0046661, HMDB0046709, HMDB0046835, HMDB0046836, HMDB0047018, HMDB0047024, HMDB0047071, HMDB0047179, HMDB0047185, HMDB0047207, HMDB0047214, HMDB0047294, HMDB0047391, HMDB0047438, HMDB0047559, HMDB0047867, HMDB0047873, HMDB0048036, HMDB0048056, HMDB0048122, HMDB0048190, HMDB0048527, HMDB0048533, HMDB0048553, HMDB0048560, HMDB0048667, HMDB0048708, HMDB0048728, HMDB0048749, HMDB0048792, HMDB0048856, HMDB0049159, HMDB0049165, HMDB0049184, HMDB0049191, HMDB0049272, HMDB0049293, HMDB0049311, HMDB0049351, HMDB0049391, HMDB0049431, HMDB0049492, HMDB0049493, HMDB0049783, HMDB0049789, HMDB0049807, HMDB0049814, HMDB0049891, HMDB0049911, HMDB0049928, HMDB0049966	TG(18:0/20:0/20:4(5Z,8Z	-0,159290045	0,030916838	0,135509021
943,5049451	HMDB0037929, HMDB37929	Tragopogonsaponin M	-0,014251406	0,307948947	0,56847381
962,4915341	HMDB0116048, HMDB0116055, HMDB0116080, HMDB0116205	CDP-DG(18:2(9Z,11Z)/a-15:0)	-0,029020978	0,950220484	0,979271001
967,4156436	HMDB0039400, HMDB39400	Basellasaponin B	-0,024483725	0,889251385	0,954337611
969,4498815	HMDB0010032, HMDB10032	PIP2(16:0/16:0)	-0,099409436	0,621039249	0,791734247
973,5422167	HMDB0116802, HMDB0116803, HMDB0116804, HMDB0116833	CL(8:0/8:0/8:0/13:0)	-0,052766496	0,18798231	0,4246647
976,6273891	HMDB0061572, HMDB61572	PS(DiMe(13,5)/DiMe(13	-0,017396634	0,393576722	0,641527233
979,5473851	HMDB0040783, HMDB40783	Vinaginsenoside R13	-0,124984818	0,019973108	0,100095114
981,7928027	HMDB0045260, HMDB0045281, HMDB0045286, HMDB0045416, HMDB0045421, HMDB0045442, HMDB0045995, HMDB0046071, HMDB0046151, HMDB0046171, HMDB0046176, HMDB0046201, HMDB0046220, HMDB0046226, HMDB0046879, HMDB0046927, HMDB0046975, HMDB0049526, HMDB0049531, HMDB0049546, HMDB0049651, HMDB0050133, HMDB0050138, HMDB0050152, HMDB0050252, HMDB0050545, HMDB0050659, HMDB0050672, HMDB0050677, HMDB0050714, HMDB0050726, HMDB0050732, HMDB0050786, HMDB0050791, HMDB0050804, HMDB0050821, HMDB0050827, HMDB0050833, HMDB0050839, HMDB0051083, HMDB0051094, HMDB0051185, HMDB0051190, HMDB0051202, HMDB0051243, HMDB0051247, HMDB0051253, HMDB0051270, HMDB0051345, HMDB0051360, HMDB0051695, HMDB0051744, HMDB0051793, HMDB0051814, HMDB0051825, HMDB0051830, HMDB0051846, HMDB0051856, HMDB0051862, HMDB0051878, HMDB0051887, HMDB0051894, HMDB0052313, HMDB0052343, HMDB0052709, HMDB0052717, HMDB0052821, HMDB0053039, HMDB0053131, HMDB0053136, HMDB0053157, HMDB0053227, HMDB0053425, HMDB0053430, HMDB0053496, HMDB0053503, HMDB0053508, HMDB0053515, HMDB0053539, HMDB0053545, HMDB0053551, HMDB0053593, HMDB0053616, HMDB0053628, HMDB0053795, HMDB0053800, HMDB0053861, HMDB0053866, HMDB0053872, HMDB0053901, HMDB0053905, HMDB0053967, HMDB0054065, HMDB0054136, HMDB0054141, HMDB0054162, HMDB0054201, HMDB0054222, HMDB0054232, HMDB0054238, HMDB0054288, HMDB0054467, HMDB0054499, HMDB0054503, HMDB0054508, HMDB0054527, HMDB0054536, HMDB0054555, HMDB0054564, HMDB0054573, HMDB0054578, HMDB0054582, HMDB0054596, HMDB0054614, HMDB0054674, HMDB0054722, HMDB0054727, HMDB0054731, HMDB0054736, HMDB0054744, HMDB0054775, HMDB0054797, HMDB0054823, HMDB0054933, HMDB0054938, HMDB0054945, HMDB0055000, HMDB0055015, HMDB0055070, HMDB0055244, HMDB0055292, HMDB0055391, HMDB0055438, HMDB0055443, HMDB0055559, HMDB0055593, HMDB0055602, HMDB0055636, HMDB0055702, HMDB0055705, HMDB0055813	TG(18:0/22:4(7Z,10Z,13	0,05770758	0,768040301	0,883342587
987,5569428	HMDB0116805, HMDB0116806, HMDB0116834, HMDB0116835, HMDB0116866, HMDB0117427	CL(8:0/8:0/8:0/14:0)	-0,003319228	0,275508598	0,541088957

m/z	HMDB ID alternatives	Suggested annotation	Comparison [vegan] - [omnivore]<br>Fold Change (log2)	p-value	p-value (BH adj.)
990,5228949	HMDB0116049, HMDB0116057, HMDB0116098, HMDB0116241	CDP-DG(18:2(9Z,11Z)/a-17:0)	-0,031545302	0,860016717	0,933916037
997,4820151	HMDB0010035, HMDB0010059, HMDB10035, HMDB10059	PIP2(16:0/18:0)	-0,042614826	0,384571785	0,636331303

*p-value is calculated for the comparison between omnivores and vegans using Student's t-test.

Appendix Table S7: Nutrient intakes in proportion to energy intake

Nutrients	Omnivore<br>*N = 24*	Vegetarian<br>*N = 10*	Vegan<br>*N = 6*	p-value*<br>*Omnivore vs. vegan*
Linoleic acid - g/MJ	0·876 [0·529 - 1·42]	1·75 [0·861 - 2·32]	2·04 [1·45 - 2·37]	<0·001
Alpha-linolenic acid - g/MJ	0·255 [0·111 - 0·41]	0·499 [0·137 - 0·944]	0·523 [0·493 - 0·689]	<0·001
EPA - mg/MJ	7·73 [0·0248 - 56·1]	0 [0 - 28·7]	0 [0 - 0]	<0·001
DHA - mg/MJ	19·2 [2·56 - 169]	2·58 [0 - 87·7]	0 [0 - 0]	<0·001
Cholesterol - g/MJ	25·4 [13·2 - 41·7]	8·22 [0·171 - 67·3]	0·152 [0·0953 - 0·283]	<0·001
Fiber - g/MJ	2·69 [1·69 - 4·3]	5·07 [2·6 - 5·95]	4·89 [3·49 - 6·18]	0·02
Thiamine (B1) - mg/MJ	0·15 [0·106 - 0·177]	0·188 [0·116 - 0·536]	0·239 [0·193 - 0·54]	<0·001
Riboflavin (B2) - mg/MJ	0·29 [0·169 - 0·46]	0·318 [0·144 - 0·49]	0·296 [0·168 - 0·483]	1
Niacin (B3) - mg/MJ	3·16 [2·28 - 4·45]	2·79 [2·25 - 5·23]	3·01 [2·31 - 5·6]	0·79
Pyridoxine (B6) - mg/MJ	0·206 [0·132 - 0·343]	0·2 [0·172 - 0·243]	0·17 [0·117 - 0·285]	0·16
Folate (B9) - ug/MJ	28·3 [20·8 - 45·8]	52·6 [31 - 83·4]	62·9 [39·7 - 104]	0·002
Cobalamine (B12) - ug/MJ	0·621 [0·322 - 1·4]	0·55 [0·123 - 1·1]	0·53 [0·191 - 0·892]	0·31
Vitamin C - mg/MJ	15·3 [3·53 - 28·8]	12·4 [7·22 - 26·9]	9 [3·68 - 20·1]	0·16
Vitamin A** - ug/MJ	87·1 [61·7 - 177]	100 [54·6 - 112]	76·4 [49·4 - 97]	0·58
Vitamin D - ug/MJ	1·45 [0·867 - 2·31]	1·5 [0·648 - 2·45]	1·34 [0·831 - 1·94]	0·73
Vitamin E - mg/MJ	1·05 [0·667 - 2·41]	1·56 [1·43 - 1·93]	1·7 [1·57 - 2·23]	0·005
Vitamin K - ug/MJ	9·71 [4·98 - 16·4]	15·6 [3·37 - 30·5]	13·5 [12·2 - 17]	0·03
Sodium - mg/MJ	293 [253 - 427]	276 [227 - 390]	290 [246 - 334]	0·31
Calcium - mg/MJ	171 [103 - 267]	188 [70·8 - 211]	140 [106 - 201]	0·58
Potassium - mg/MJ	489 [351 - 606]	402 [269 - 555]	411 [347 - 455]	0·11
Magnesium - mg/MJ	45·9 [35·5 - 64]	60·9 [43·4 - 67·7]	59 [49 - 63·5]	0·03
Iron - mg/MJ	1·38 [1·12 - 2·35]	2·2 [1·58 - 2·52]	2·11 [1·85 - 2·34]	0·006
Iodine - ug/MJ	30·8 [17·3 - 46·4]	15·6 [7·89 - 21·7]	12·7 [10 - 16·1]	<0·001
Phosphorus - mg/MJ	208 [149 - 254]	178 [147 - 201]	146 [121 - 160]	0·009
Zinc - mg/MJ	1·45 [1·11 - 1·92]	1·47 [1·04 - 2·52]	1·45 [0·991 - 2·46]	0·66

*p-value is calculated for the comparison between omnivores and vegans using age- and sex-adjusted permutation test.
**Vitamin A is presented as retinol activity equivalents (RAE).

Appendix Table S8: Exact p-values of Figure 4

Metabolite	p-value*
Alpha-linolenic acid (ALA)	0.14
Docosahexaenic acid (DHA)	**0.00089**
Carnitine 18:3	0.12
Carnitine 18:4	**0.00012**
Carnitine 20:4	0.16
Lysophosphatidylcholine 16:0	0.07
Lysophosphatidylcholine 18:1	**0.039**
Lysophosphatidylethanolamine 16:0	0.79
Lysophosphatidylethanolamine 18:1	0.71

*by age- and sex-adjusted permutation tests with Benjamini-Hochberg correction for multiple comparisons.

References

S1. Nissinen K, Korkalo L, Vepsäläinen H. Accuracy in the estimation of children's food portion sizes against a food picture book by parents and early educators. *J Nutr Sci.* 2018;**7**:e35.

S2. Nissinen K, Sillanpää H, Korkalo L, Roos E, Erkkola M. Annoskuvakirja: lasten ruokamäärien arvioinnin avuksi. Helsinki: Helsingin yliopisto : Seinäjoen ammattikorkeakoulu : Samfundet Folkhälsan, 2015.

S3. Korkalo L, Nissinen K, Skaffari E et al. The Contribution of Preschool Meals to the Diet of Finnish Preschoolers. *Nutrients*, 2019;**11(7)**:1531.

S4. Vásquez-Caicedo AL, Bell S, Hartmann B. D2.2.9 Report on collection of rules on use of recipe calculation procedures including the use of yield and retention factors for imputing nutrient values for composite foods. *European Food Information Resource Network* 2008. Available online: `http://toolbox.foodcomp.info/References/RecipeCalculation/EuroFIR%20recipe%20calculation%20procedures%20including%20yield%20and%20retention%20factors.pdf`

S5. Engle-Stone R, Haskell MJ, Ndjebayi AO et al. Plasma retinol-binding protein predicts plasma retinol concentration in both infected and uninfected Cameroonian women and children. *J Nutr* 2011;**141(12)**:2233–41.

S6. Talsma EF, Verhoef H, Brouwer ID, Mburu-de Wagt AS, Hulshof PJM, Melse-Boonstra A. Proxy markers of serum retinol concentration, used alone and in combination, to assess population vitamin A status in Kenyan children: a cross-sectional study. *BMC Med* 2015;**13**:30.

S7. Thas O, De Neve J, Clement L, Ottoy J-P. Probabilistic index models. *Royal Statistical Society*. 2012;**74(4)**:623–71.

S8. De Schryver M, De Neve J. A tutorial on probabilistic index models: Regression models for the effect size P(Y1 < Y2). *Psychol Methods*. 2019 Aug;**24(4)**:403–18.

S9. Davison AC, Hinkley DV. Nonparametric permutation tests. In: Davison AC, Hinkley DV. Cambridge: Cambridge University Press; 1997.

S10. Subramanian A, Tamayo P, Mootha VK, Mukherjee S, Ebert BL et al. Gene set enrichment analysis: a knowledge-based approach for interpreting genome-wide expression profiles. *Proc Natl Acad Sci U S A*. 2005 Oct 25;**102(43)**:15545–50.

S11. Xiang X, Han Y, Neuvonen M, Laitila J, Neuvonen PJ, Niemi M. High performance liquid chromatography-tandem mass spectrometry for the determination of bile acid concentrations in human plasma. *J Chromatogr B Analyt Technol Biomed Life Sci.* 2010 Jan 1;**878(1)**:51–60.

S12. Erhardt JG, Estes JE, Pfeiffer CM, Biesalski HK, Craft NE. Combined Measurement of Ferritin, Soluble Transferrin Receptor, Retinol Binding Protein, and C-Reactive Protein by an Inexpensive, Sensitive, and Simple Sandwich Enzyme-Linked Immunosorbent Assay Technique. *J Nutr.* 2004 Nov;**134(11)**:3127–32.

S13. Miettinen TA, Tilvis RS, Kesaniemi YA. Serum cholestanol and plant sterol levels in relation to cholesterol metabolism in middle-aged men. *Metabolism* 1989;**38**:136-40.

S14. Miettinen TA, Tilvis RS, Kesaniemi YA. Serum plant sterols and cholesterol precursors reflect cholesterol absorption and synthesis in volunteers of a randomly selected male population. *Am J Epidemiol* 1990; **131**: 20-31.

S15. Cook JD, Flowers CH, Skikne BS. The quantitative assessment of body iron. *Blood.* 2003 May 1;**101(9)**:3359–64.

S16. *HUSLAB is an accredited clinical laboratory of Helsinki University Hospital. Their methods and contact information for each method can be found online from HUSLAB manual with reference number for each method (listed in Table S1): `https://huslab.fi/ohjekirja/[REFERENCENUMBERHERE].html`

S17. **Yhtyneet Medix Laboratoriot webpage has contact information about iodide method, reference number for method used in this study was 1984 (U-I): `https://www.yml.fi/tuotekuvaus_show.php?tuotenro=831`
