# Supplementary material for: Vegan diet in young children remodels metabolism and challenges the statuses of essential nutrients
Source: EMBO Mol Med. 2021 Jan 20;13(2):e13492. doi: 10.15252/emmm.202013492 (PMC7863396; doi:10.15252/emmm.202013492)
Supplement: Supplementary file 2 — Expanded View Figures PDF [file EMMM-13-e13492-s002.pdf]

# Expanded View Figures

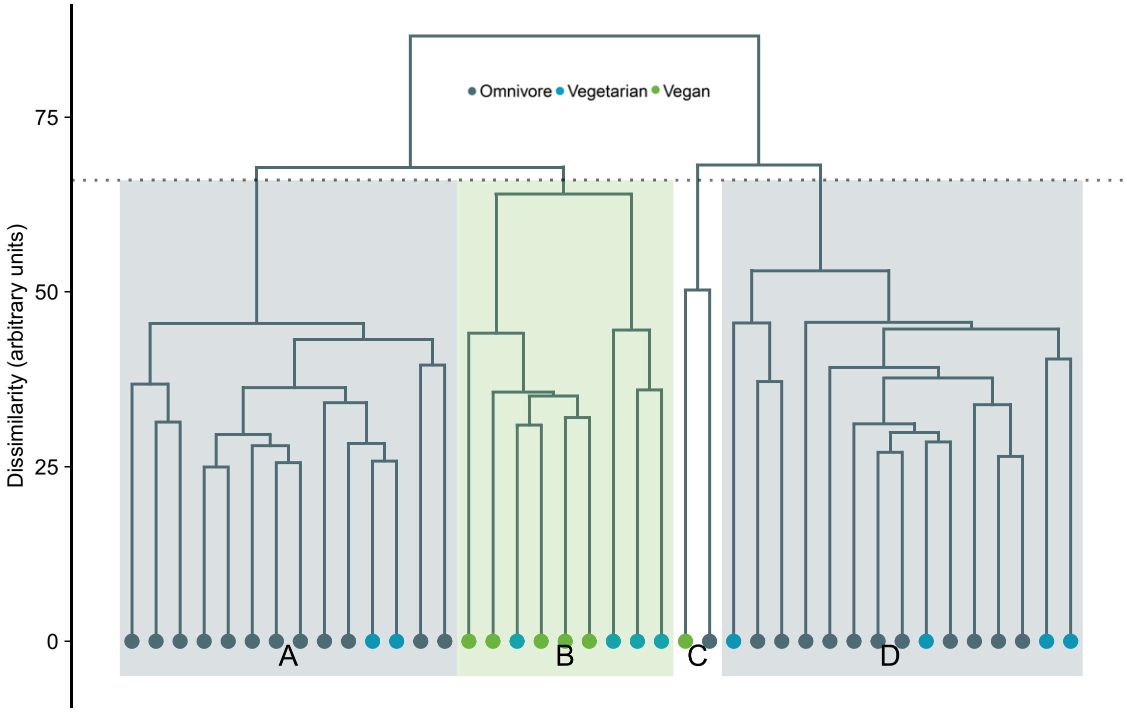


**Figure EV1. Hierarchical clustering of participants based on untargeted biomarkers in blood.**
Hierarchical clustering of participants based on the detected 872 metabolites in untargeted metabolomics from serum. Hierarchical clustering was performed using Ward's method and Euclidean distance as the measure of dissimilarity. *n* = 24 omnivores, 10 vegetarians, six vegans.

Source data are available online for this figure.

© 2021 The Authors
